# Phosphine-Catalyzed Regio- and Stereoselective Umpolung Addition of Amides to Alkynoates: Access to Complex α,β-Dehydroamino Acid Derivatives

**DOI:** 10.1021/acs.orglett.5c04858

**Published:** 2026-01-29

**Authors:** Nicklas W. Buchbinder, Kyle L. Dunnavant, Andrew D. Bage, Owen N. Beck, Kaamia R. Harris, Reilly K. Gwinn, Webster L. Santos

**Affiliations:** Department of Chemistry, 1757 Virginia Tech, 900 West Campus Drive, Blacksburg, Virginia 24061, United States

## Abstract

Accessing complex α,β-dehydroamino acids remains challenging due to the instability of the enamine product during N-terminal deprotection. We report a mild, organocatalytic method for the installation of primary amides on the α-carbon of alkynoates that avoids N-terminal deprotection. The PBu_3_ catalyst is key to umpolung reactivity and affords α,β-dehydroamino acids in good yield with excellent (*Z*)-selectivity. The utility of this reaction was demonstrated in the synthesis of two natural products: a 2,5-diketopiperazine and scutianene M.

## Graphical abstract

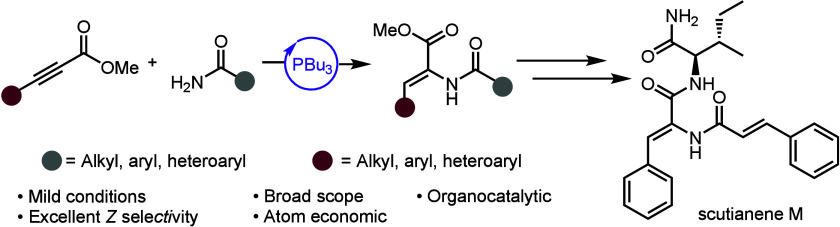

α,β-Dehydroamino acids are noncanonical amino acids that contain unsaturation between the α- and β-side chain carbons. The site of unsaturation restricts flexibility, decreases bond length, and increases bond angles of the amino acid side chain when compared to proteogenic amino acids. Therefore, α,β-dehydroamino acids have been utilized in synthetic chemistry,^1–3^ drug discovery (Scheme 1a),^4–6^ biochemistry,^7–9^ and material science.^10,11^ Many natural products isolated from bacteria, fungi and plants feature α,β-dehydroamino acid moieties (Scheme 1a).^12,13^

**Scheme 1:**
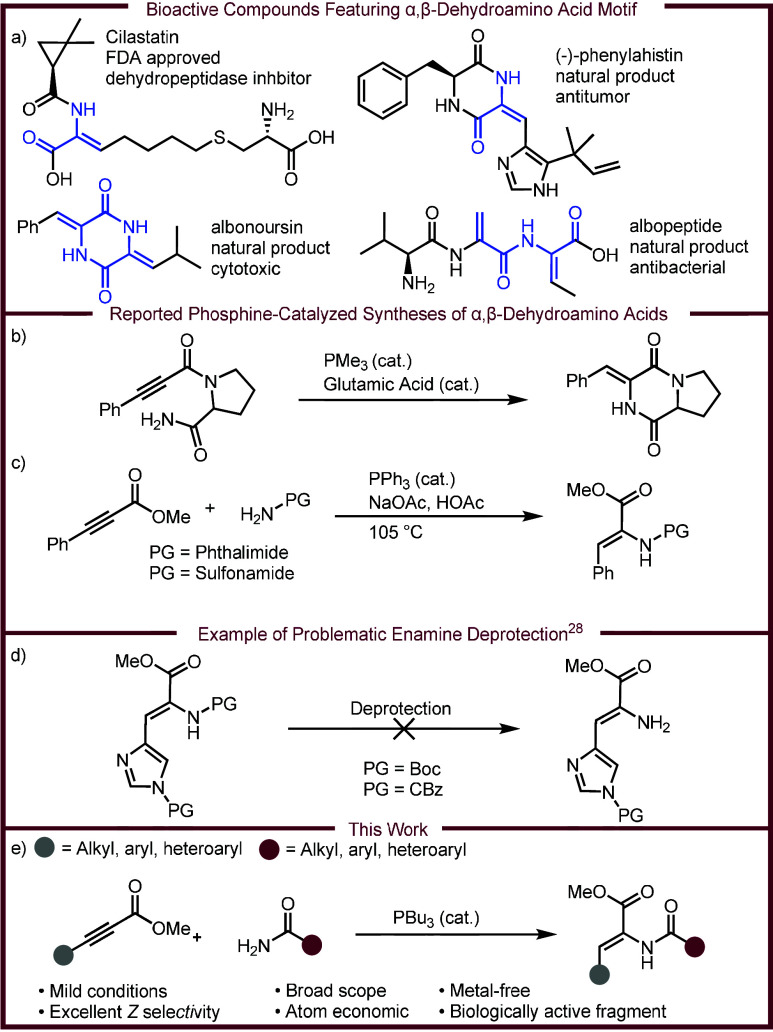
Motivation for Development of the Described Reaction

Considering the broad applications of α,β-dehydroamino acids, the synthesis of these molecules has been heavily explored. Early methodologies such as the Erlenmeyer–Plöchl reaction use excess reagents and elevated temperatures.^14^ Specialized protecting groups have been studied for improving the aldol-type condensations, but require additional steps for protection and deprotection.^15^ Stoichiometric reactions involving the elimination of a halide or oxygen based leaving group have been developed, but lack catalytic efficiency.^16–18^ The Horner–Wadsworth–Emmons (HWE) reaction is commonly used to access α,β-dehydroamino acid derivatives, but special fragments, stoichiometric reagents, and multistep synthesis is generally required.^19,20^ Transition-metal catalysis (Cu and Pd) has also been employed to access α,β-dehydroamino acid derivatives.^21–23^ More recently, Zhang developed a phosphine-catalyzed intramolecular cyclization of primary amides and employed it toward the synthesis of spirotryprostatins (Scheme 1b).^24^ Seminal work from Trost reported a triarylphosphine-catalyzed addition of phthalimides and sulfonamides to alkynoates resulting in N-protected α,β-dehydroamino acids; however, the use of elevated temperature, buffered conditions and use of phthalimides/sulfonamides limits broad application (Scheme 1c).^25^ Since Trost’s seminal work, the phosphine-catalyzed nucleophilic α-addition of alkynoates has been studied including substrates such as pyridones,^26^ phosphite,^27^ sulfur,^28^ and others;^29^ however, these are limited to terminal acetylenes, low yields and *E*/*Z* selectivity.

Many research efforts have focused on the synthesis of N-protected α,β-dehydroamino acids; however, this approach has limited application toward the synthesis of complex α,β-dehydroamino acids due to instability of the deprotected enamine.^30^ Accessing complex dehydroamino acids by deprotection and elongation of the N-terminus has been a long-standing issue in organic synthesis. For example, during the total synthesis of isoroquefortine C, deprotection of the enamine failed and ultimately a different synthetic route was required (Scheme 1d).^31^ Despite the limitations of N-protected α,β-dehydroamino acids, many of the synthetic methods that result in α,β-dehydroamino acids have focused on substrates that are protected on the N-terminus.

Conversely, we envisioned a method that avoids N-terminus deprotection. Instead, we envision coupling complex amides (including amino acid derivatives) to alkynoates directly through an intermolecular phosphine-catalyzed umpolung addition (Scheme 1e).

Our investigations on the phosphine-catalyzed hydroamidation of alkynoates began by treating our model substrates methyl-3-phenylpropiolate **1a** and benzamide **2a** with catalytic tri-*n*-butyl phosphine (PBu_3_) in toluene at 40 °C for 1 h. The α,β-dehydroamino acid derivative (*Z*)-**3a** was formed in a 62% yield with >95:5 *Z*/*E* selectivity (Table 1, entry 1). When P­(NEt_2_)_3_, PCy_3_ or PPh_3_ were used instead of PBu_3_ lower efficiency was observed (entries 2–4). Decreasing the temperature to 25 °C resulted in a loss of yield; however, increasing the temperature to 70 °C afforded (*Z*)-**3a** in a 68% yield without deterioration of selectivity (entries 6–7). A survey of reaction solvents revealed that toluene was optimal for both selectivity and yield (entries 8–10). The optimal conditions were found when the reaction time was extended to 16 h (in toluene at 40 °C), which afforded (*Z*)-**3a** in >95% yield (entry 11). Using Trost’s optimal conditions only a 3% yield of (*Z*)-**3a** was detected (entry 12).

**Table 1:**
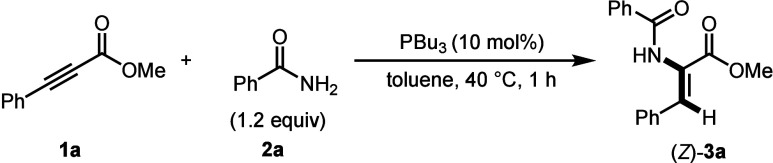
Reaction Optimization

Entry	Variation from above conditions	Yield	*Z*/*E*
1	None	62%	>95:5
2	P(NEt_2_)_3_ instead of PBu_3_	3%	>95:5
3	PCy_3_ instead of PBu_3_	8%	94:6
4	PPh_3_ instead of PBu_3_	0%	-
5	dppe instead of PBu_3_	5%	22:78
6	25 °C instead of 40 °C	44%	92:8
7	70 °C instead of 40 °C	68%	>95:5
8	^b^MeCN instead of toluene	33%	93:6
9	^b^hexanes instead of toluene	45%	96:4
10	^b^DCE instead of toluene	36%	90:10
11	16 h instead of 1 h	>95%	>95:5
12	PPh_3_, NaOAc/HOAc (1:1), 105 °C	3%	>95:5

a 0.125 mmol **1**, 0.0125 mmol PBu_3_, 0.15 mmol **2**, 0.25 M in toluene. Yield determined using ^1^H NMR with mesitylene as the internal standard. All reactions performed in duplicate and averaged.

b 70 °C.

With the optimal conditions in hand, our attention turned to determining the scope and limitations of the reaction. Our model substrate **3a** was isolated in a 70% yield (Scheme 2). When Cinnamamide was used, α,β-dehydroamino acid derivative **3b** was isolated in a 57% yield. Alkyl amides afforded α,β-dehydroamino acid derivatives **3c** and **3d** in low to modest yields (37% and 21%, respectively). A Naproxen derived amide afforded **3e** in a 25% yield. Trifluoroacetamide resulted in a 57% yield of compound **3f.** Heterocyclic amides reacted smoothly, resulting in α,β-dehydroamino acid derivatives **3g** and **3h** in 91% and 78% yields, respectively. Phthalimide and sulfonamide were also found to be competent substrates for this reaction (**3i** and **3j**).

**Scheme 2:**
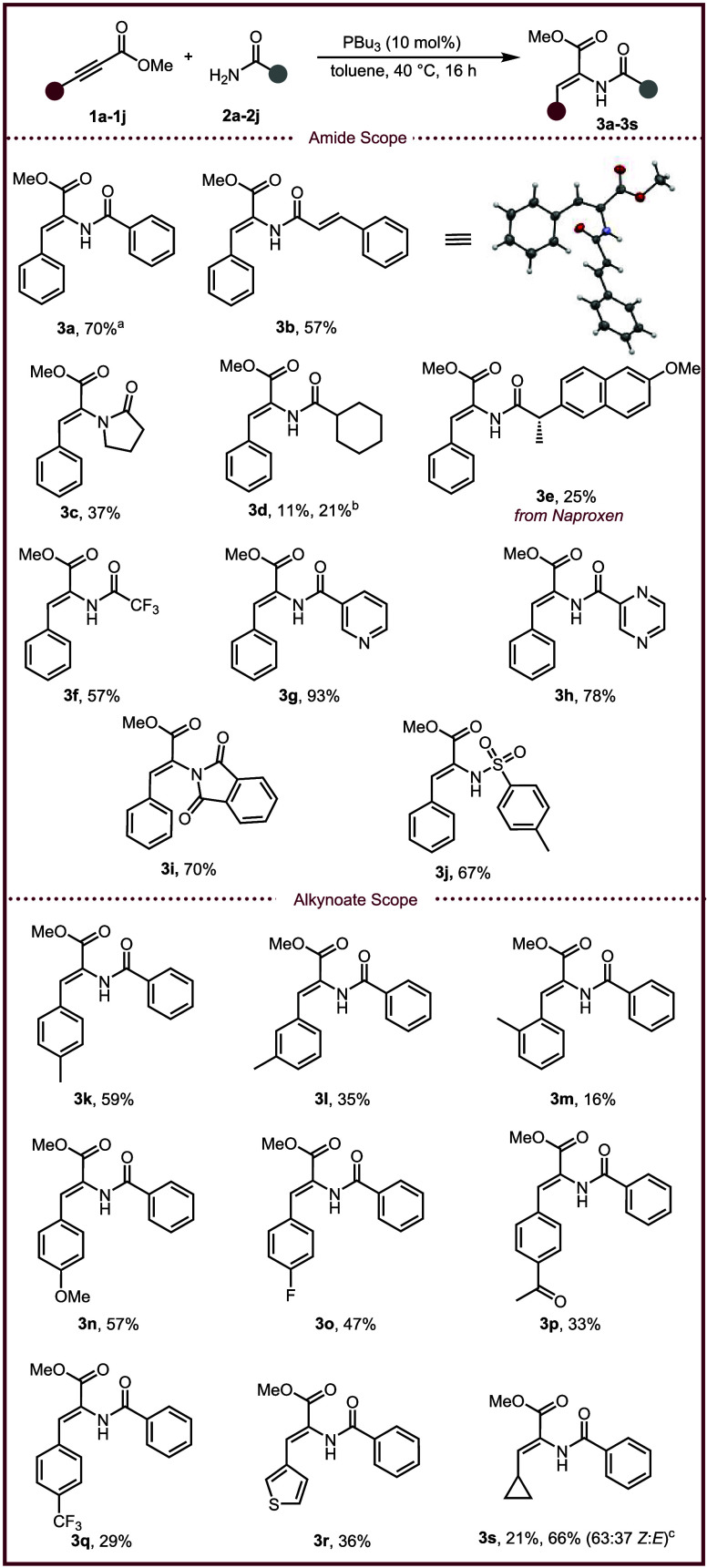
Substrate Scope^a^

a 70 °C.
b 20 mol % PBu_3_.
c 20 mol % K_2_CO_3_.
d Yields refer to isolated yields. >99:1 *Z*/*E* selectivity unless otherwise noted, based on isolated yield of each isomer. 0.25 mmol **1**, 0.025 mmol PBu_3_, 0.30 mmol **2**, 0.25 M in toluene.

To evaluate functional group tolerance on the alkynoate, a series of substrates were synthesized. First, *p*-, *m*- and *o*-tolylalkynoates were found to afford α,β-dehydroamino acid derivatives **3k**–**3m** in low to modest yield with *para*-substitution performing the best. A methoxy derivative, **3n**, was synthesized in a 57% yield. Electron deficient alkynoates resulted in low to modest yields of α,β-dehydroamino acid derivatives **3o**–**3r** (29–47%). When a cyclopropyl substrate **1j** (product **3s**) was used, low reactivity was observed (21% yield); however, introduction of a substoichiometric amount of base (K_2_CO_3_) improved the yield to 66% but with diminished selectivity (63:37, *Z*/*E*).

Next, a series of biologically relevant substrates were tested (Scheme 3a). First, an intermediate used in the synthesis of Atevirdine,^32^ an FDA-approved non-nucleoside reverse transcriptase inhibitor, **3t** was synthesized in 68% yield with modest selectivity (83:17, *Z*/*E*). Various protected amino amides were also evaluated to afford α,β-dehydroamino acid containing dipeptides. The reaction displayed good tolerance for *tert*-butyl carbamates (Boc) as the N-terminus protecting group, with compounds **3u**–**3y** being isolated in good yield (51–66%). Notably, the yield of **3u** improved to 58% when the reaction was performed on a 3.0 mmol scale. A glycine derived Boc-protected amino amide resulted in a 16% yield of compound **3y** using the optimal conditions; however, increasing the temperature to 90 °C resulted in a 43% yield of the desired α,β-dehydroamino acid. When 9-fluorenylmethyl carbamates (Fmoc) were used as the N-terminus protecting group, low yield was achieved (**3z**, **3aa**), presumably due to phosphine-catalyzed deprotection (14–19%). Methyl-protected tyrosine dehydroamino acid derivatives **3ab** and **3ac** were afforded in good yields (54–74%). A triphenylmethyl-protected (trityl) dehydro-histidine substrate **3ad** was formed in 74% yield revealing that the reaction tolerates trityl protecting groups. Boc-protected dehydro-tryptophan was also synthesized using this method with substrate **3ae** being isolated in an 88% yield with 87:13 *Z*/*E* selectivity. To evaluate whether the reaction epimerized the optically pure amino acid fragments, **3s** was Boc-deprotected and converted into Mosher’s amide (**S6**). Characterization of **S6** indicated the presence of a single diastereomer and suggest that that epimerization does not occur under the reaction conditions (see Supporting Information for details)

**Scheme 3:**
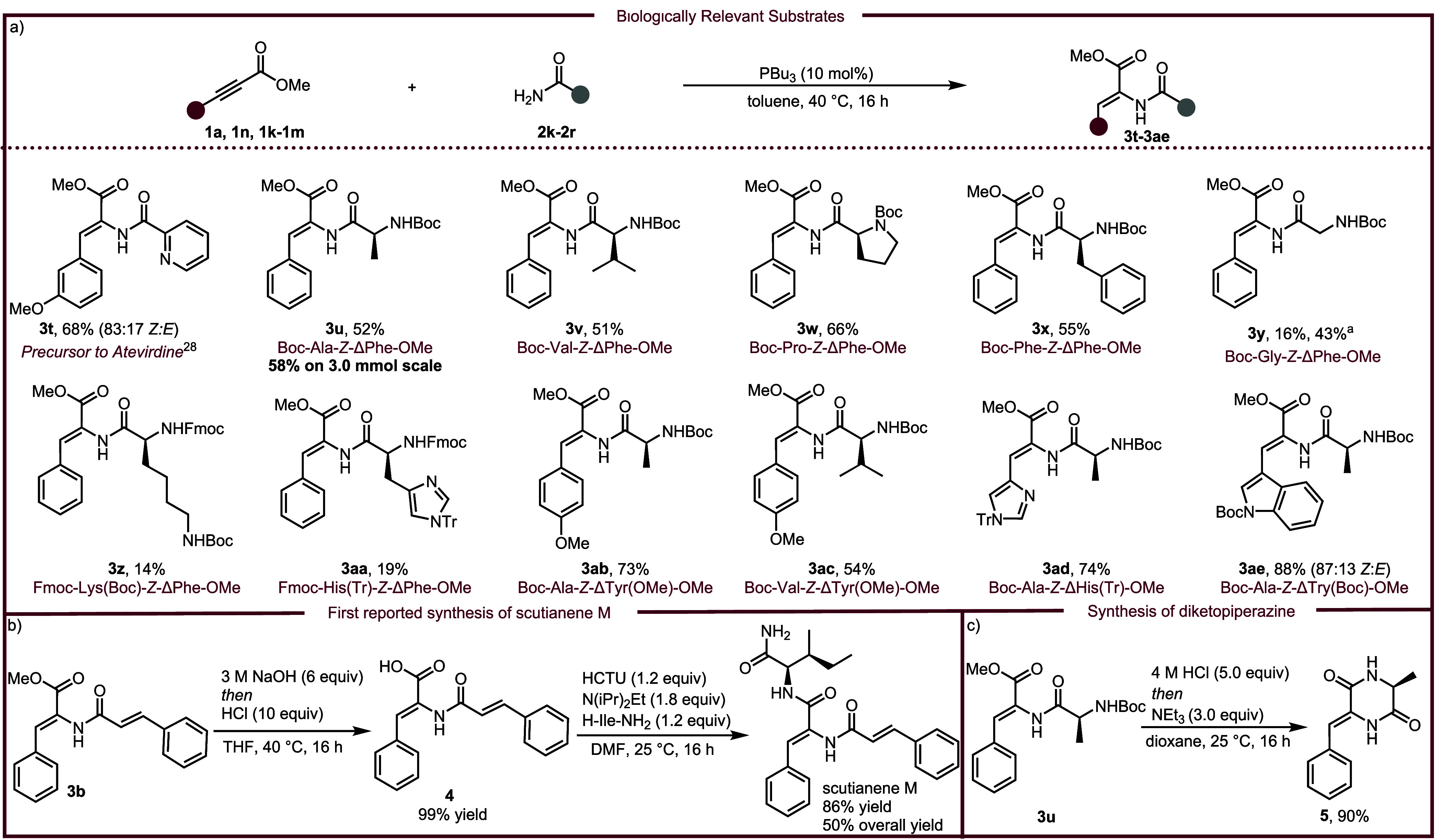
Wide Array of Biologically Relevant Substrates and Applications^a^

a 90 °C.
b Yields refer to isolated yields. >99:1 *Z*/*E* selectivity unless otherwise noted, based on isolated yield of each isomer. 0.25 mmol **1**, 0.025 mmol PBu_3_, 0.30 mmol **2**, 0.25 M in toluene.

To demonstrate the utility of this transformation, the α,β-dehydroamino acids were chemically modified. First, α,β-dehydroamino acid **3b** was hydrolyzed under basic conditions to afford compound **4** in a near quantitative yield without isomerization of the double bond geometry (Scheme 3b). Compound **4** was then coupled to isoleucinamide (H-Ile-NH_2_) to afford scutianene M in an overall yield of 50% from commercially available starting material. To our knowledge, this is the first reported synthesis of scutianene M, an alkaloid natural product that exhibits a minimum inhibitory concentration (MIC) of 12.5 μg/mL against *Enterococcus spp*.^33^ Other structural motifs commonly found in natural products include 2,5-diketopiperazines.^34^ Utilizing our standard reaction conditions, we were able to access 2,5-diketopiperazine **5** by treating α,β-dehydroamino acid **3u** with 4 M HCl in dioxane followed by neutralization with triethylamine (Scheme 3c). Compound **5** is both a natural product (isolated from *Nocardiopsis alba* SCSIO 03039) and an intermediate in the synthesis of other natural products (puniceloid D).^35,36^ Importantly, the synthesis of **5** was previously achieved in a 3% yield over 5 steps; however, using our methodology, compound **5** was furnished in a 52% yield over 3 steps.

Based on literature precedence, a plausible mechanism is proposed in Scheme 4.^37–39^ First, tri-*n*-butylphosphine attacks alkynoate **1**, resulting in the zwitterionic phosphonium-allenolate **6**. Amide **2** undergoes deprotonation and subsequent nucleophilic addition with intermediate **6** to form ylide intermediate **7**. Proton transfer quenches the ylide furnishing the zwitterion intermediate **8**, which collapses the catalyst to generate the (*Z*)-product. While the origin of *Z*-selectivity is unknown, initial mechanistic studies provide a clue. When (*E*)-**3a** was subjected to the reaction conditions, crude NMR analysis revealed that the (*E*)-isomer isomerized into a mixture of (*E*)- and (*Z*)-isomers. Conversely, when (*Z*)-**3a** was subjected to the same experiment, no isomerization occurred (See Supporting Information for details). These experiments suggest that a mixture of (*E*)- and (*Z*)-isomers may be generated during the catalytic cycle, but the mixture is isomerized into the (*Z*)-isomer.

**Scheme 4:**
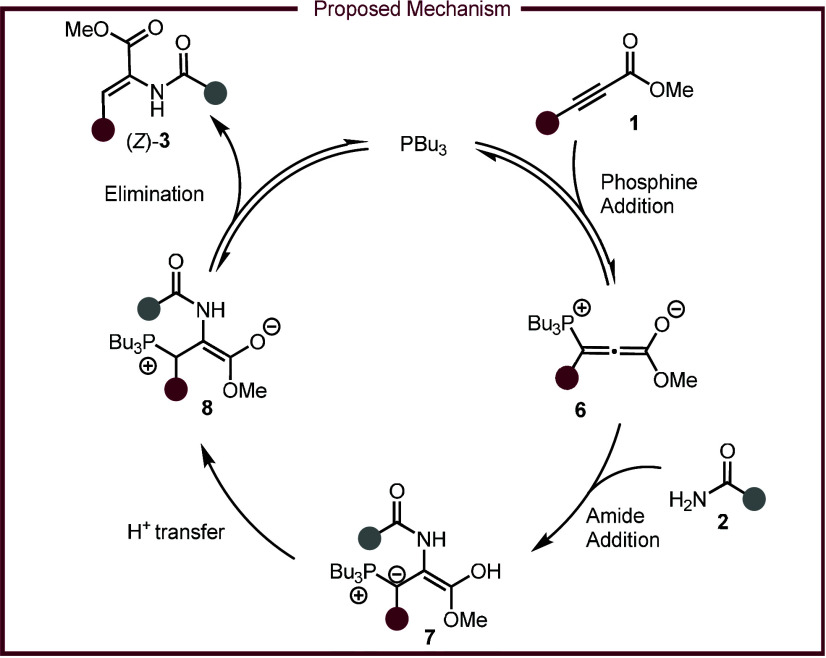
Proposed Mechanism

In conclusion, a simple and mild method for the synthesis of α,β-dehydroamino acids is disclosed. Alkyl, aryl and heteroaryl substitution on either coupling partner is tolerated. The reaction is void of transition metals and the catalyst is an inexpensive standard commodity. The α,β-dehydroamino acid products can be utilized for the synthesis of scutianene natural products as well as a medicinal chemistry fragment.

## Supplementary Material



## Data Availability

The data underlying this study are available in the published article and its online Supporting Information.

## Notes

Synthetic procedures and characterization data, mechanistic study, NMR spectra, and crystallographic data (PDF)

## †

Chemistry Research Laboratory, Department of Chemistry, Twelve Mansfield Road, Oxford OX1 3TA, U.K
