# Supplementary material for: Phosphine-Catalyzed Regio- and Stereoselective Umpolung Addition of Amides to Alkynoates: Access to Complex α,β-Dehydroamino Acid Derivatives
Source: Org Lett. 2026 Jan 29;28(20):6159–64. doi: 10.1021/acs.orglett.5c04858 (PMC13200229; doi:10.1021/acs.orglett.5c04858)
Supplement: Supplementary file 1 [file ol5c04858_si_001.pdf]

Phosphine-Catalyzed Regio- and Stereoselective Umpolung Addition of Amides to Alkynoates: Access to Complex α,β-Dehydroamino Acid Derivatives

Nicklas W. Buchbinder, Kyle L. Dunnavant, Andrew D. Bage, Owen N. Beck, Kaamia Harris, Reilly K. Gwinn, Webster L. Santos*

Department of Chemistry, Virginia Tech, Blacksburg, Virginia 24061, United States
Email: santosw@vt.edu

Contents

1. General Information 3
2. General Procedures 4
   Synthesis of Alkynoates 4
   Synthesis of **S1l** 5
   Synthesis of **1m** 6
   Synthesis of Amides 8
   Synthesis of α,β-dehydroamino acids 8
   3.0 mmol Scale Synthesis of Substrate **3s** 9
3. **Table S1**: References for Starting Material Compounds 10
4. **Table S2**: Full Optimization Table 15
5. Characterization for α,β-dehydroamino acids 17
6. Mechanistic Study 30
   Photocatalyzed isomerization of α,β-dehydroamino acid **3a** 30
   Isomerization Study 31
7. Epimerization Study 33
8. Crystallographic Data 40
9. NMR Spectra 43
10. References 121

1. General Information

Materials

Chemicals were obtained from commercial sources (Sigma Aldrich, Fisher Scientific, Acros Organics, Ambeed Inc., Oakwood Chemical, Combi-Blocks) unless otherwise noted. Anhydrous THF (Fisher), toluene (Fisher), dichloromethane (Fisher) and acetonitrile (Fisher) were obtained from the Innovative Technology Pure SolvMD solvent purification system (drying agent: alumina; under argon atmosphere).

Methods

Reactions were performed using standard Schlenk techniques under a nitrogen atmosphere unless otherwise noted. All glassware was flame-dried before use. All reported temperatures are based upon external heating plate temperature. The room temperature was approximately 25 °C.

Column Chromatography

Flash column chromatography was performed on Teledyne ISCO CombiFlash systems, using SiliaFlash P60 40-63 μm, 60 Å. TLC analyses were performed using Silicycle aluminum backed silica gel F-254 plates.

NMR Spectroscopy

NMR spectroscopic experiments were performed using Agilent 400-MR 400 MHz, Agilent U4-DD2 400 Hz and Bruker Avance II 500 MHz spectrometers. Chemical shifts are reported in δ ppm, coupling constants, *J*, are reported in Hz (to 1 decimal place). ^{1}H NMR spectra are referenced to the residual protonated solvent signal ($CDCl_3$, 7.26 ppm; CD_3OD, 3.31 ppm; DMSO-d_6, 2.50 ppm; acetone-d_6, 2.05 ppm) and ^{13}C NMR spectra are referenced to the deuterated solvent signal ($CDCl_3$, 77.16 ppm; CD_3OD, 49.00 ppm; DMSO-d_6, 39.52 ppm; acetone-d_6; 29.84 ppm) and are ^{1}H decoupled. Signal multiplicities reported as singlet (s), doublet (d), apparent doublet (appd), triplet (t), quartet (q), multiplet (m).

Mass Spectrometry

Mass spectrometry was performed at VT-MSI HRMS facility. High resolution mass spectra (HRMS) were recorded on Agilent 6220 using flow injection analysis (FIA). LC flow is 0.1 ml/min with a composition of 25:75 water: acetonitrile with 0.1% formic acid. Run times were 1 minute.

2. General Procedures

Synthesis of Alkynoates

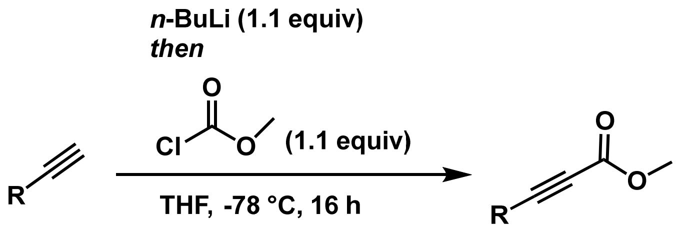

S1

1

Scheme S1

General procedure 1: A round bottom flask was charged with a stir bar, flame dried and put under an inert atmosphere using standard *Schlenk* technique. Dry THF (7.83 mL, 0.25 M) and terminal alkyne (1.96 mmol, 1.0 equiv) were added. The reaction flask was added to a dry ice/acetone bath. Then, *n*-BuLi (2.15 mmol, 1.1 equiv) was added dropwise, and the reaction was allowed to stir for 30 minutes. Next, methyl-chloroformate (2.15 mmol, 1.1 equiv) was added dropwise (add slowly to prevent ketone formation). The reaction was allowed to stir overnight and come to room temperature. Upon completion, the reaction was quenched with water and extracted thrice with ethyl acetate, dried over sodium sulfate and concentrated under vacuum. The desired alkynoates were purified using flash chromatography (0-20% EtOAc:Hex gradient).

Synthesis of **S1l**

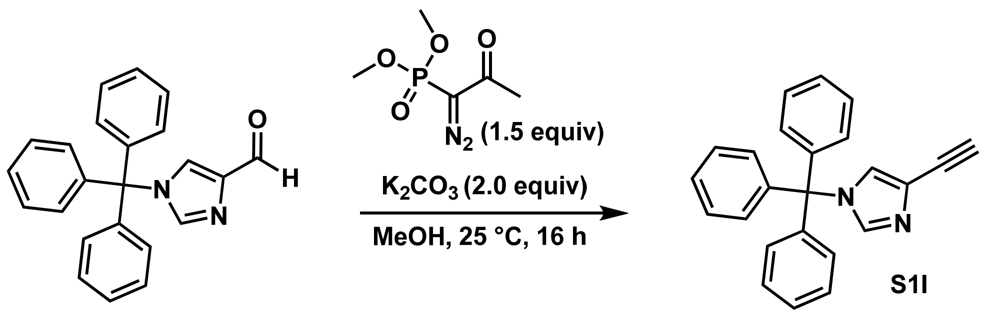

Scheme S2: Ohira-Bestmann reaction.

General procedure 2: 4-ethynyl-1-trityl-*1H*-imidazole (**S1l**) was synthesized using a modified literature procedure.[1] A round bottom flask was charged with a stir bar and flame dried. 1-trityl-*1H*-imidazole-4-carbaldehyde (501 mg, 1.48 mmol, 1.0 equiv) and potassium carbonate (409 mg, 2.96 mmol, 2.0 equiv) were added to the round bottom flask. The reaction vessel was sealed and put under an inert atmosphere using standard *Schlenk* technique. 10% Dimethyl (1-diazo-2-oxopropyl)phosphonate solution in MeCN (5.17 mL, 2.22 mmol, 1.5 equiv) and MeOH (6 mL, 0.25 M) were then added. The reaction was allowed to stir for 16 hours. Upon completion the reaction was quenched with brine and extracted with DCM thrice. The organic layers were combined and dried over sodium sulfate. Crude material was concentrated under vacuum to afford 4-ethynyl-1-trityl-*1H*-imidazole (**S1l**) (490 mg, 1.47 mmol, 99% yield) as a white solid. Characterization matched previous reports.[2]

Synthesis of **1m**

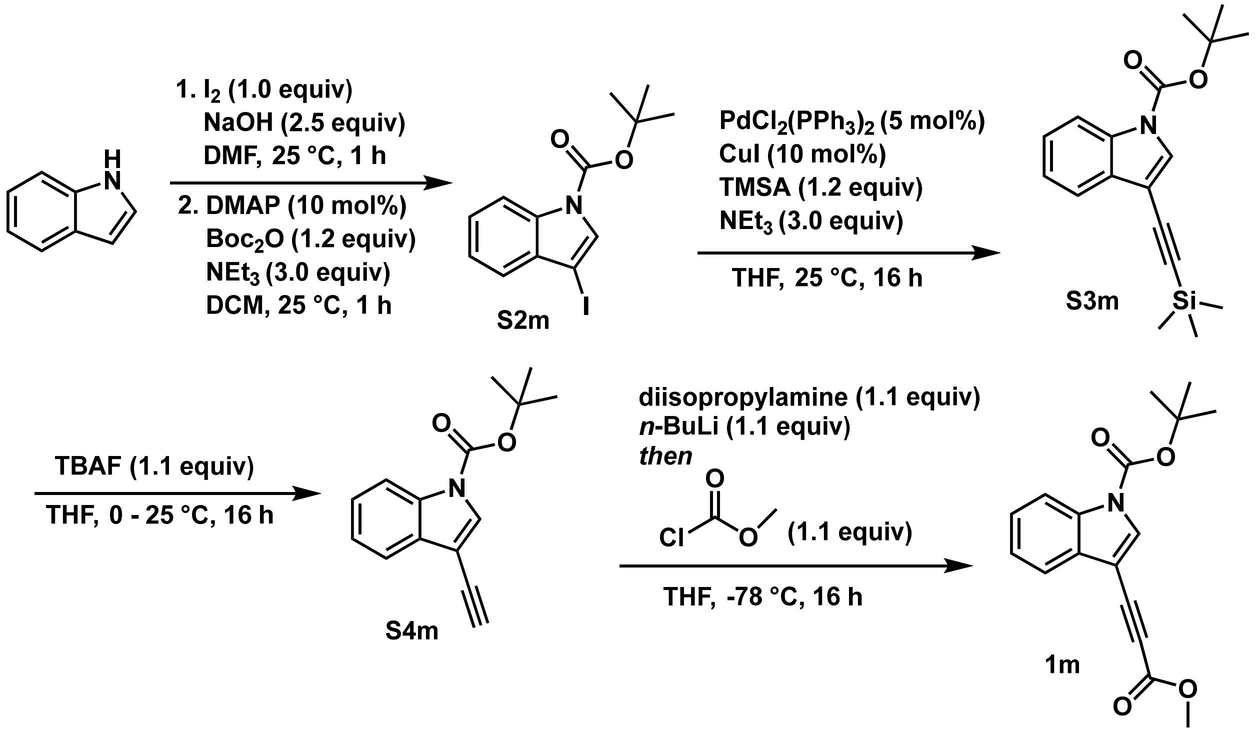

Scheme S3: Synthetic scheme for alkynoate **1m**.

General procedure 3a: *tert*-butyl 3-iodo-1*H*-indole-1-carboxylate (**S2m**) was synthesized using a modified literature procedure.[3] A round bottom flask was charged with a stir bar and flame dried. A solution of 1*H*-indole (500.0 mg, 4.27 mmol, 1.0 equiv) in DMF (15 mL, 28 M) was added to the round bottom. Then, NaOH (426.9 mg, 10.7 mmol, 2.5 equiv) was added and the solution was allowed to stir for 30 minutes. Finally, iodine (1.083 g, 4.27 mmol, 1.0 equiv,) was added and the reaction was allowed to stir for an additional hour. Upon consumption of the starting material via TLC the reaction was quenched with an aqueous solution of lithium bromide and extracted thrice with ethyl acetate. The organic layers were combined and dried over sodium sulfate. Crude material was concentrated under vacuum. To the crude material 4-dimethylaminopyridine (52.5 mg, 0.43 mmol, 10 mol%,), di-*tert*-butyl-dicarbonate (1.176 mL, 5.12 mmol, 1.2 equiv,), triethylamine (1.784 mL, 12.8 mmol, 3.0 equiv) and DCM (40 mL, 0.11 M) were added at 25 °C. The reaction was allowed to stir for 1 hour. The desired *tert*-butyl 3-iodo-1*H*-indole-1-carboxylate (**S2m**) was purified using flash chromatography (0-5% EtOAc:Hex gradient) as a colorless oil in 81% (1.183 g, 3.45 mmol) yield across two steps. Characterization matched previous reports.[3]

General procedure 3b: *tert*-butyl 3-((trimethylsilyl)ethynyl)-1*H*-indole-1-carboxylate (**S3m**) was synthesized using a modified literature procedure.[4] A round bottom flask was charged with a stir bar and flame dried. *tert*-butyl 3-iodo-1*H*-indole-1-carboxylate (**S2m**)

(350.0 mg, 1.02 mmol, 1.0 equiv), bis(triphenylphosphine)palladium dichloride (35.8 mg, 0.05 mmol, 5 mol%) and copper iodide (19.4 mg, 0.10 mmol, 10 mol%) were added next. The round bottom was sealed and put under an inert atmosphere using standard *Schlenk* technique. Finally, dry THF (5 mL, 0.2 M), triethylamine (426 μL, 3.06 mmol, 3.0 equiv) and trimethylsilyl acetylene (174 μL, 1.22 mmol, 1.2 equiv) were added. The reaction was allowed to stir for 16 hours. Upon completion the reaction was quenched with brine and extracted with DCM thrice. The organic layers were combined and dried over sodium sulfate. Crude material was concentrated under vacuum. *tert*-butyl 3-((trimethylsilyl)ethynyl)-1*H*-indole-1-carboxylate (**S3m**) was purified using flash chromatography (isocratic hexanes) as a brown oil in 82% (263 mg, 838.0 μmol) yield. Characterization matched previous reports.[4]

General procedure 3c: *tert*-butyl 3-ethynyl-1*H*-indole-1-carboxylate **(S4m)** was synthesized using a modified literature procedure.[5] A round bottom flask was charged with a stir bar and flame dried. The flask was charged with *tert*-butyl 3-((trimethylsilyl)ethynyl)-1*H*-indole-1-carboxylate (**S3m**) (149.4 mg, 477 μmol, 1.0 equiv), of dry THF (1.8 mL, 0.27 M) and added to an ice bath (0 °C). A 1 M solution of tetrabutylammonium fluoride (0.524 mL, 524 μmol, 1.1 equiv) was then added to the round bottom flask dropwise. The reaction was allowed to stir for 1 hour. Upon completion the reaction was quenched with brine and extracted with DCM thrice. The organic layers were combined and dried over sodium sulfate. Crude material was concentrated under vacuum. *tert*-butyl 3-ethynyl-1*H*-indole-1-carboxylate (**S4m**) was purified using flash chromatography (0-5% EtOAc:Hex gradient) as a yellow oil in 86% (91.1 mg, 378 μmol) yield. Characterization matched previous reports.[5]

General procedure 3d: *tert*-butyl 3-(3-methoxy-3-oxoprop-1-yn-1-yl)-1*H*-indole-1-carboxylate (**1m**) was synthesized using a modified general procedure 1. A round bottom flask was charged with a stir bar and flame dried. The round bottom was sealed and put under an inert atmosphere using standard *Schlenk* technique. The round bottom was added to a dry ice/acetone bath. Dry THF (3.0 mL), diisopropylamine (95.9 μL, 684 μmol, 1.1 equiv) and 2.5 M *n*-BuLi in hexanes (274 μL, 684 μmol, 1.1 equiv) were added. A 2.5 M solution of *tert*-butyl 3-ethynyl-1*H*-indole-1-carboxylate (**S4m**) in THF (60.0 μL, 622 μmol, 1.0 equiv) was added dropwise. After 1 hour, methyl-chloroformate (57.8 μL, 746 μmol, 1.2 equiv) was added dropwise (add slowly to prevent ketone formation). The reaction was allowed to stir for 16 hours. Upon completion the reaction was quenched with brine and extracted with DCM thrice. The organic layers were combined and dried over sodium sulfate. Crude material was concentrated under vacuum. *tert*-butyl 3-(3-methoxy-3-oxoprop-1-yn-1-yl)-1*H*-indole-1-carboxylate (**1m**) was purified using flash chromatography (0-10% EtOAc:Hex gradient) as a yellow oil in a 43% (80.1 mg, 622 μmol) yield. Characterization matched previous reports.[6]

Synthesis of Amides

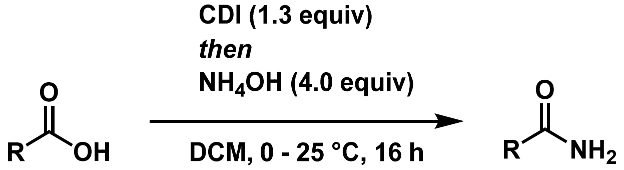

Scheme S4: Synthesis of Amide Starting Material.

General procedure 4: Modified from literature procedure.[7] A round bottom flask was charged with a stir bar and flame dried. Carboxylic acid (0.500 mmol, 1.0 equiv) and carbonyldiimidazole (0.650 mmol, 1.3 equiv) were added. The round bottom was sealed and put under an inert atmosphere using standard *Schlenk* technique. Dry DCM (5.0 mL, 0.25 M) was added, and the reaction flask was added to an ice bath. The reaction was allowed to stir until the evolution of CO_2 gas stopped (roughly 30 minutes). 25 wt% ammonium hydroxide (2.00 mmol, 4.0 equiv) was then added using a syringe and the reaction was allowed to stir at room temperature for 16 hours. Upon completion, the reaction was quenched with water and extracted thrice with DCM, dried over sodium sulfate and concentrated under vacuum. The desired amides were purified using flash chromatography (0-10% MeOH:DCM gradient).

Synthesis of α,β-dehydroamino acids

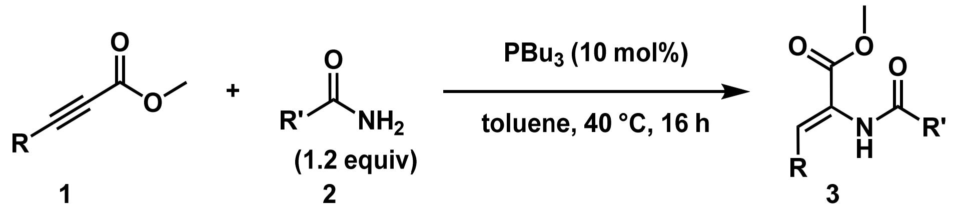

Scheme S5: Synthesis of α,β-dehydroamino acids.

General Procedure 5: To a flame dried 2-dram vial: a stir bar, alkynoate **1** (0.250 mmol, 1.0 equiv) and amide **2** (0.300 mmol, 1.2 equiv) were added. The vial was sealed with a septa lid and put under an inert atmosphere using standard *Schlenk* technique. Then, toluene (1 mL, 0.25 M) and tri-*n*-butylphosine (6.2 µL, 0.025 mmol, 10 mol%) were added. The vial was added to a preheated oil bath at 40 °C. The reaction was allowed to stir for 16 hours. The reaction was then opened and 2 mL of EtOAc was added. TLC analysis was conducted using 40% EtOAc:Hex. The α,β-dehydroamino acids generally have an Rf of 0.25 under these conditions. To the mixture, celite was added and volatiles were removed under vacuum. The α,β-dehydroamino acids **3** were purified using flash chromatography (0-60% EtOAc:Hex gradient). The *E*:*Z* isomers were separable if both isomers formed.

3.0 mmol Scale Synthesis of Substrate **3s**

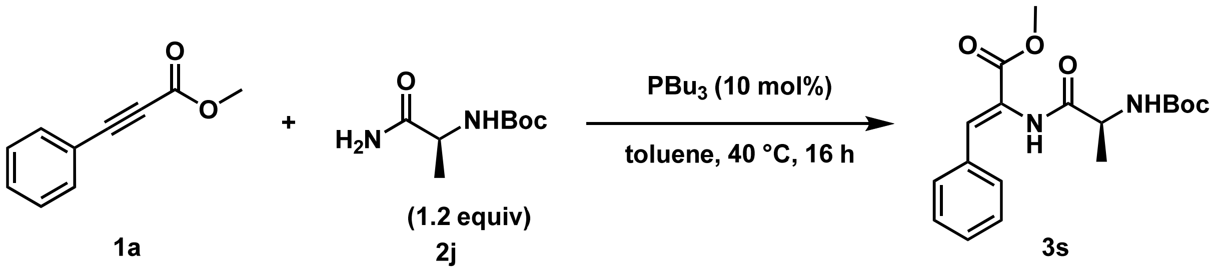

Scheme S6: 3.0 mmol scale synthesis of α,β-dehydroamino acid **3s**.

Procedure 6: To a flame dried 50 mL round bottom: a stir bar, alkynoate **1a** (481.0 mg, 3.00 mmol,1.0 equiv) and amide **2j** (678.0 mg, 3.60 mmol,1.2 equiv) were added. The round bottom was sealed with a septa lid and put under an inert atmosphere using standard *Schlenk* technique. Then, toluene (12 mL, 0.25 M) and tri-*n*-butylphosine (74 μL, 0.300 mmol, 10 mol%) were added. The round bottom was added to a preheated oil bath at 40 °C. The reaction was allowed to stir for 16 hours. The reaction was then opened and 10 mL of EtOAc was added. To the mixture, celite was added and volatiles were removed under vacuum. The α,β-dehydroamino acids **3s** was purified using flash chromatography (0-60% EtOAc:Hex gradient) as a white solid (606 mg, 1.74 mmol, 58%).

3. **Table S1**: References for Starting Material Compounds

Code	Structure	Reference
**1a**[8]	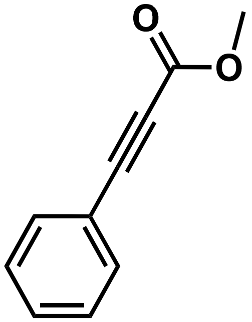	Fritzemeier, R. G.; Nekvinda, J.; Vogels, C. M.; Rosenblum, C. A.; Slebodnick, C.; Westcott, S. A.; Santos, W. L., *Angew. Chem. Int. Ed.* **2020,** *59* (34), 14358-14362.
**1b**[9]	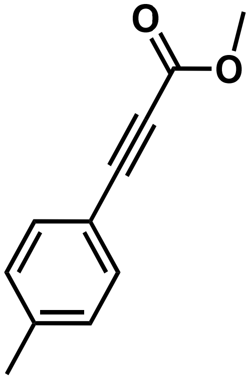	Fritzemeier, R.; Gates, A.; Guo, X.; Lin, Z.; Santos, W. L., *J. Org. Chem.* **2018,** *83* (17), 10436-10444.
**1c**[9]	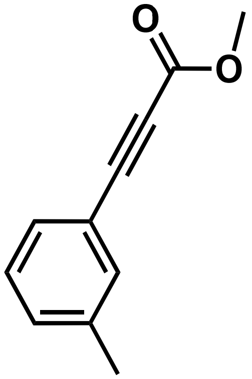	Fritzemeier, R.; Gates, A.; Guo, X.; Lin, Z.; Santos, W. L., *J. Org. Chem.* **2018,** *83* (17), 10436-10444.
**1d**[8]	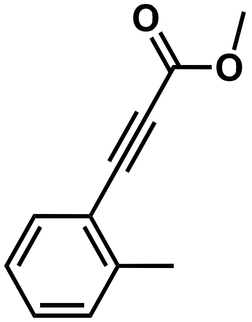	Fritzemeier, R. G.; Nekvinda, J.; Vogels, C. M.; Rosenblum, C. A.; Slebodnick, C.; Westcott, S. A.; Santos, W. L., *Angew. Chem. Int. Ed.* **2020,** *59* (34), 14358-14362.

**1e**[8]	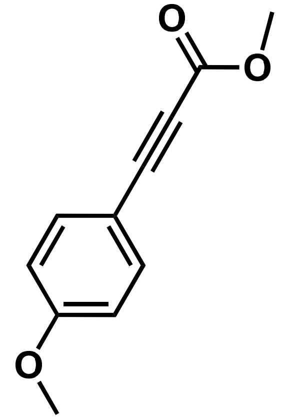	Fritzemeier, R. G.; Nekvinda, J.; Vogels, C. M.; Rosenblum, C. A.; Slebodnick, C.; Westcott, S. A.; Santos, W. L., *Angew. Chem. Int. Ed.* **2020,** *59* (34), 14358-14362.
**1f**[8]	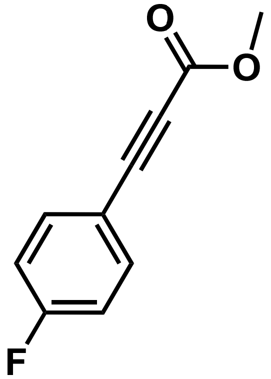	Fritzemeier, R. G.; Nekvinda, J.; Vogels, C. M.; Rosenblum, C. A.; Slebodnick, C.; Westcott, S. A.; Santos, W. L., *Angew. Chem. Int. Ed.* **2020,** *59* (34), 14358-14362..
**1g**[8]	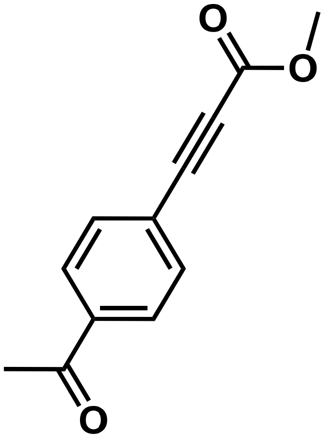	Fritzemeier, R. G.; Nekvinda, J.; Vogels, C. M.; Rosenblum, C. A.; Slebodnick, C.; Westcott, S. A.; Santos, W. L., *Angew. Chem. Int. Ed.* **2020,** *59* (34), 14358-14362..
**1h**[9]	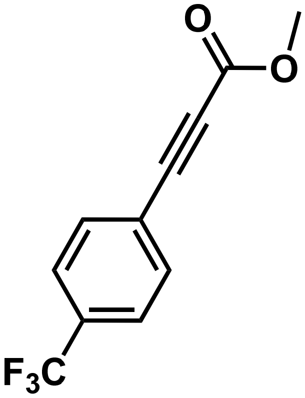	Fritzemeier, R.; Gates, A.; Guo, X.; Lin, Z.; Santos, W. L., *J. Org. Chem.* **2018,** *83* (17), 10436-10444.

**1i**[10]	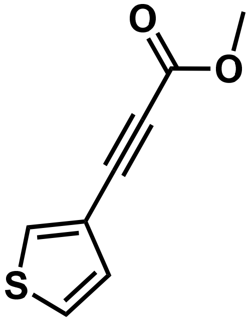	Fujimoto, H.; Kusano, M.; Kodama, T.; Tobisu, M. *J. Am. Chem. Soc.* **2021,** *143* (44), 18394-18399.
**1j**[8]	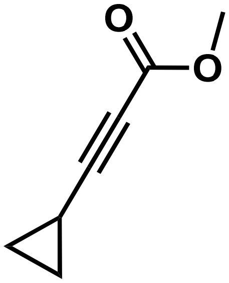	Fritzemeier, R. G.; Nekvinda, J.; Vogels, C. M.; Rosenblum, C. A.; Slebodnick, C.; Westcott, S. A.; Santos, W. L., *Angew. Chem. Int. Ed.* **2020,** *59* (34), 14358-14362.
**1k**[8]	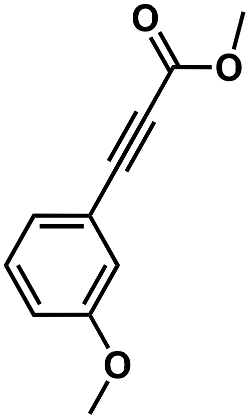	Fritzemeier, R. G.; Nekvinda, J.; Vogels, C. M.; Rosenblum, C. A.; Slebodnick, C.; Westcott, S. A.; Santos, W. L., *Angew. Chem. Int. Ed.* **2020,** *59* (34), 14358-14362.
**S1l**[2]	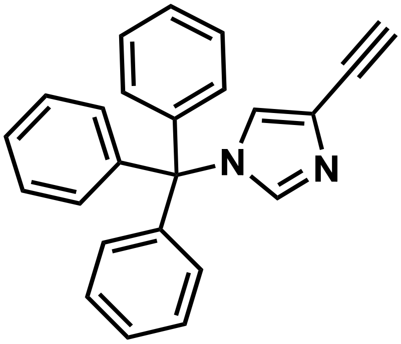	B. Dolensky and K. L. Kirk, *J. Fluor. Chem.* **2003**, *124*, 105-110.

**1l**[2]	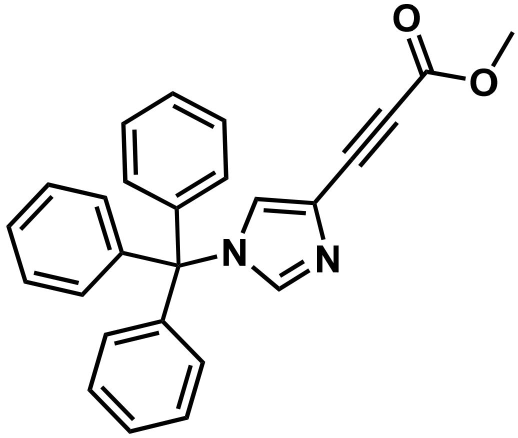	B. Dolensky and K. L. Kirk, *J. Fluor. Chem.* **2003**, *124*, 105-110.
**S1m**[11]	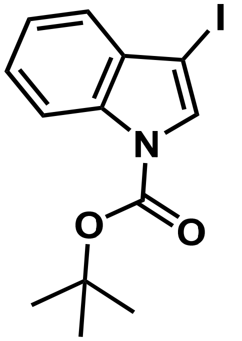	Mothes, C.; Lavielle, S.; Karoyan, P., *J. Org. Chem.* **2008,** *73* (17), 6706-6710.
**S2m**[4]	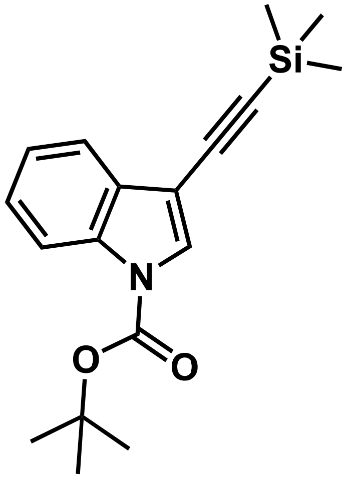	He, W.; Li, C.; Zhang, L., *J. Am. Chem. Soc.* **2011,** *133* (22), 8482-8485.
**S3m**[5]	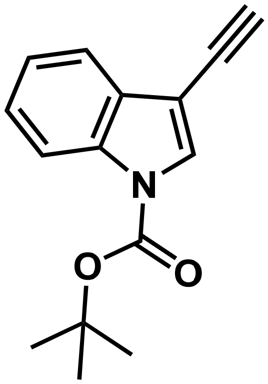	Wang, W.; Zhou, S.; Li, L.; He, Y.; Dong, X.; Gao, L.; Wang, Q.; Song, Z., *J. Am. Chem. Soc.* **2021,** *143* (29), 11141-11151.

**1m**[6]	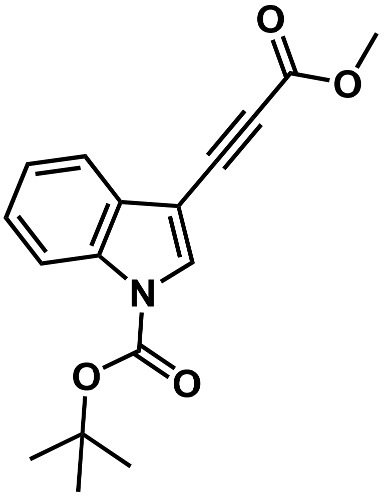	Michaelides, I. N.; Darses, B.; Dixon, D. J., *Org. Lett.* **2011,** *13* (4), 664-667.
**2e**[12]	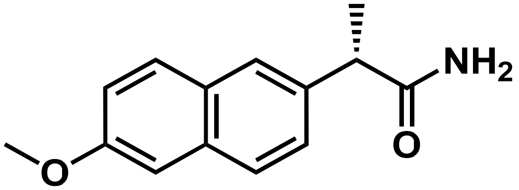	Patel, M. A.; Kapdi, A. R., Ambient- *Chem. Asian J.* **2023,** *18* (22), e202300672.
**2o**[13]	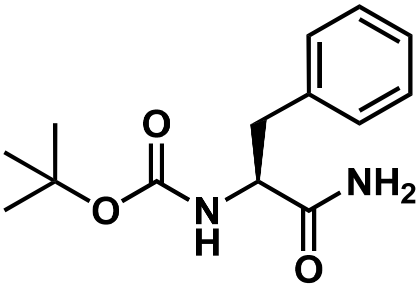	Inman, M.; Dexter, H. L.; Moody, C. J., *Org. Lett.* **2017,** *19* (13), 3454-3457.
**2p**[14]	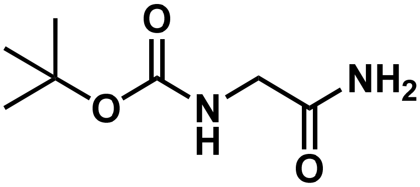	Schneider, T. L.; Walsh, C. T.; O'Connor, S. E., *J. Am. Chem. Soc.* **2002,** *124* (38), 11272-11273.
**2q**[15]	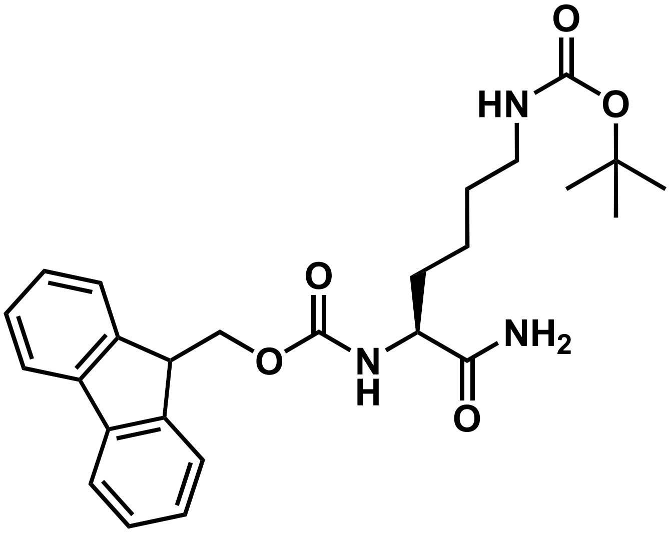	Singh, Y.; Sokolenko, N.; Kelso, M. J.; Gahan, L. R.; Abbenante, G.; Fairlie, D. P., *J. Am. Chem. Soc.* **2001,** *123* (2), 333-334.

**2r**[16]	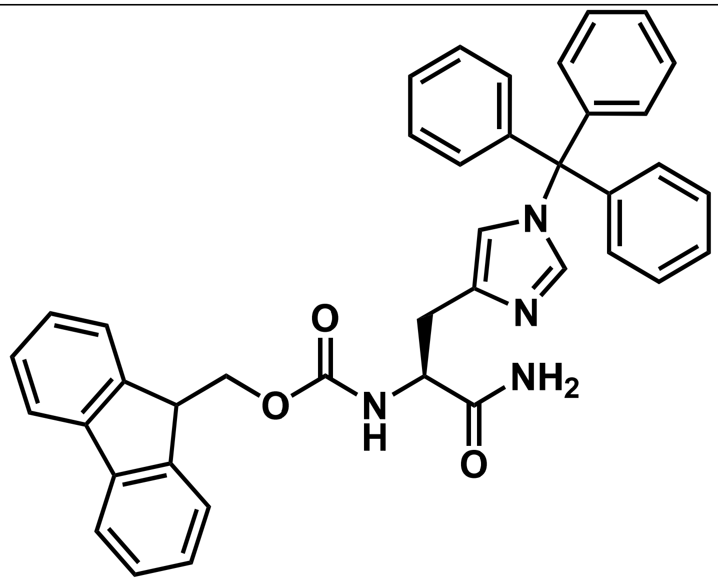	Maucort, C.; Vo, D. D.; Aouad, S.; Charrat, C.; Azoulay, S.; Di Giorgio, A.; Duca, M., *ACS Med. Chem. Lett.* **2021,** *12* (6), 899-906.
**2s**[17]	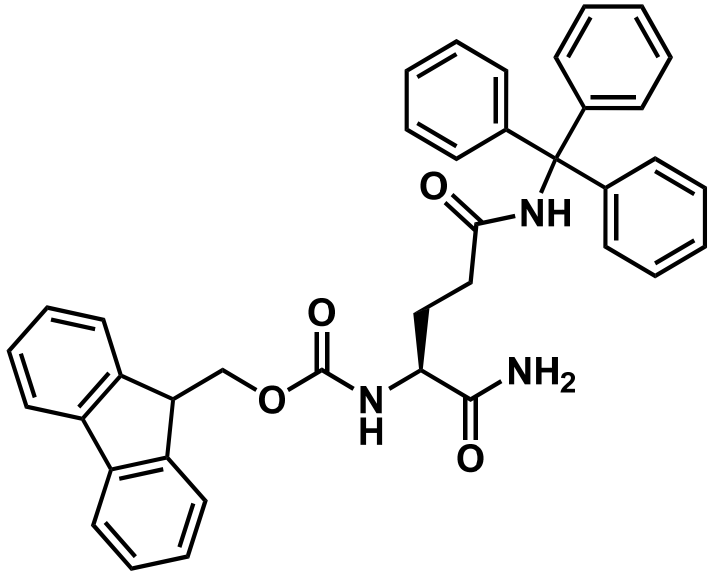	Ngo, C.; Fried, W.; Aliyari, S.; Feng, J.; Qin, C.; Zhang, S.; Yang, H.; Shanaa, J.; Feng, P.; Cheng, G., *J. Med. Chem.* **2023,** *66* (17), 12237-12248.

4. **Table S2**: Full Optimization Table

Entry	Catalyst	Catalyst loading	Temp	Solvent	Amide Loading	Rxn Conc.	Time	Yield	*Z:E*
1	PBu_3	10 mol%	40 °C	toluene	1.2 equiv	0.25 M	1 h	62%	>95:5
2	$P(NEt_2)_3$	10 mol%	40 °C	toluene	1.2 equiv	0.25 M	1 h	3%	>95:5
3	$P(Cy)_3$	10 mol%	40 °C	toluene	1.2 equiv	0.25 M	1 h	8%	94:6
4	$P(Ph)_3$	10 mol%	40 °C	toluene	1.2 equiv	0.25 M	1 h	0%	-

5	dppe	10 mol%	40 °C	toluene	1.2 equiv	0.25 M	1 h	5%	22:78
6	Xantphos	10 mol%	40 °C	toluene	1.2 equiv	0.25 M	1 h	0%	-
7	Triphos	10 mol%	40 °C	toluene	1.2 equiv	0.25 M	1 h	20%	24:76
8	PBu_3	5 mol%	40 °C	toluene	1.2 equiv	0.25 M	1 h	34%	88:12
9	PBu_3	10 mol%	0 °C	toluene	1.2 equiv	0.25 M	1 h	13%	74:26
10	PBu_3	10 mol%	25 °C	toluene	1.2 equiv	0.25 M	1 h	44%	92:8
11	PBu_3	10 mol%	70 °C	toluene	1.2 equiv	0.25 M	1 h	68%	>95:5
12	PBu_3	10 mol%	70 °C	MeCN	1.2 equiv	0.25 M	1 h	33%	93:6
13	PBu_3	10 mol%	70 °C	Hexanes	1.2 equiv	0.25 M	1 h	45%	>95:5
14	PBu_3	10 mol%	70 °C	DCE	1.2 equiv	0.25 M	1 h	36%	90:10
15	PBu_3	10 mol%	70 °C	EtOH	1.2 equiv	0.25 M	1 h	0%	-
16	PBu_3	10 mol%	70 °C	toluene	2.0 equiv	0.25 M	1 h	66%	>95:5
17	PBu_3	10 mol%	70 °C	toluene	1.2 equiv	0.125 M	1 h	39%	>95:5
18	PBu_3	10 mol%	70 °C	-	1.2 equiv	Neat	1 h	27%	94:6
19	PBu_3	10 mol%	70 °C	toluene	1.2 equiv	0.50 M	1 h	32%	>95:5
20	PBu_3	10 mol%	70 °C	toluene	1.2 equiv	0.25 M	1 h	75%	>95:5
21	PBu_3	10 mol%	70 °C	toluene	1.2 equiv	0.25 M	16 h	>95%	>95:5
22	PBu_3	10 mol%	40 °C	toluene	1.2 equiv	0.25 M	16 h	>95%	>95:5
23[a]	PPh_3	10 mol%	105 °C	toluene	1.0 equiv	0.25 M	16 h	3%	>95:5

Table S2: Average of two trials. Yield and *Z*:*E* selectivity determined by ^1H NMR with mesitylene as an internal standard. [a]50 mol% HOAc, 50 mol% NaOAc, single experiment instead of duplicate.

5. Characterization for α,β-dehydroamino acids

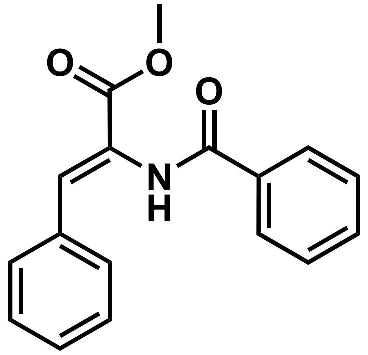

Modified from general procedure 5, methyl (*Z*)-2-benzamido-3-phenylacrylate (**3a**) was isolated using flash chromatography (0-60% EtOAc:Hex gradient) as a white solid (49.2 mg, 0.175 mmol, 70% yield) using 70 °C instead of 40 °C. **^{1}H NMR** (400 MHz, $CDCl_3$) δ 7.87 (d, *J* = 7.4 Hz, 2H), 7.80 (br s, 1H), 7.55 (t, *J* = 7.4 Hz, 1H), 7.52 - 7.48 (m, 5H), 7.33 (m, 3H), 3.85 (s, 3H). **^{13}C NMR** (101 MHz, $CDCl_3$) δ 166.0, 134.0, 133.7, 132.3, 132.0, 129.8, 129.6, 128.9, 128.7, 127.6, 124.4, 52.9. One carbon is missing due to overlapping. This data agrees with literature reports.[18]

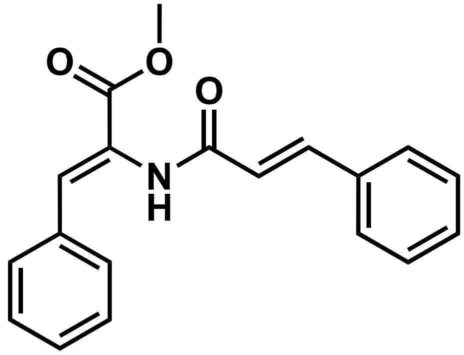

Following general procedure 5, methyl (*Z*)-2-cinnamamido-3-phenylacrylate (**3b**) was isolated using flash chromatography (0-60% EtOAc:Hex gradient) as a white solid (43.9 mg, 0.143 mmol, 57% yield). **^{1}H NMR** (600 MHz, $CDCl_3$) δ 7.69 (d, *J* = 15.6 Hz, 1H), 7.56 – 7.45 (m, 4H), 7.43 (br s, 1H), 7.42 – 7.28 (m, 6H), 6.56 (d, *J* = 15.6 Hz, 1H), 3.87 (s, 3H). **^{13}C NMR** (151 MHz, $CDCl_3$) δ 166.0, 164.3, 143.4, 134.6, 134.0, 131.8, 130.3, 129.9, 129.6, 129.0, 128.8, 128.2, 124.1, 119.6, 53.0. **HRMS** (ESI) m/z $[M+H]^+$ calcd for $C_{19}H_{18}NO_3^+$ 308.1281; Found 308.1279.

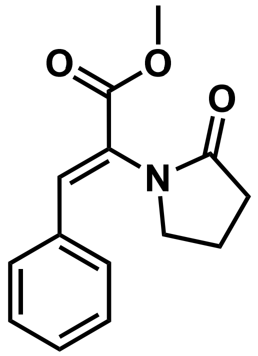

Following general procedure 5, methyl (*Z*)-2-(2-oxopyrrolidin-1-yl)-3-phenylacrylate (**3c**) was isolated using flash chromatography (0-60% EtOAc:Hex gradient) as a colorless solid (22.3 mg, 0.093 mmol, 37% yield). **^{1}H NMR** (500 MHz, $CDCl_3$) δ 7.68 (s, 2H), 7.53 – 7.45 (m, 2H), 7.40 – 7.35 (m, 3H), 3.84 (s, 3H), 3.48 (t, *J* = 7.0 Hz, 2H), 2.51 (t, *J* = 8.1 Hz, 2H), 2.16 (tt, *J* = 7.7 Hz, 7.4 Hz, 2H). **^{13}C NMR** (126 MHz, $CDCl_3$) δ 176.6, 164.8, 138.9, 133.0, 130.3, 129.7, 129.0, 127.0, 52.8, 48.1, 30.8, 19.5. This data agrees with literature reports.[19]

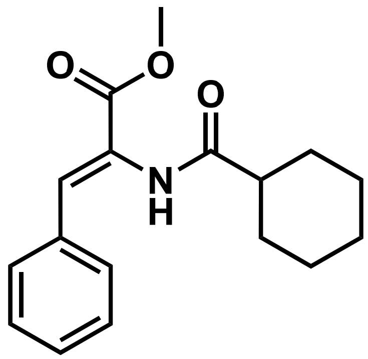

Modified from general procedure 5, methyl (*Z*)-2-(cyclohexanecarboxamido)-3-phenylacrylate (**3d**) was isolated using flash chromatography (0-60% EtOAc:Hex gradient) as a white solid (15.2 mg, 0.053 mmol, 21% yield) using 20 mol% PBu_3 instead of 10 mol% PBu_3. **^{1}H NMR** (500 MHz, $CDCl_3$) δ 7.45 – 7.42 (m, 2H), 7.38 – 7.29 (m, 4H), 7.01 (s, 1H), 3.84 (s, 3H), 2.27 (tt, *J* = 11.7, 3.6 Hz, 1H), 1.96 (d, *J* = 12.9 Hz, 2H), 1.82 (d, *J* = 12.3 Hz, 2H), 1.69 (d, *J* = 11.2 Hz, 2H), 1.50 (qd, *J* = 12.2, 3.2 Hz, 2H), 1.37 – 1.28 (m, 2H). **^{13}C NMR** (126 MHz, $CDCl_3$) δ 174.3, 166.0, 134.2, 131.6, 129.7, 129.4, 128.6, 124.4, 52.8, 45.7, 29.9, 29.4, 25.8, 25.7. This data agrees with literature reports.[20]

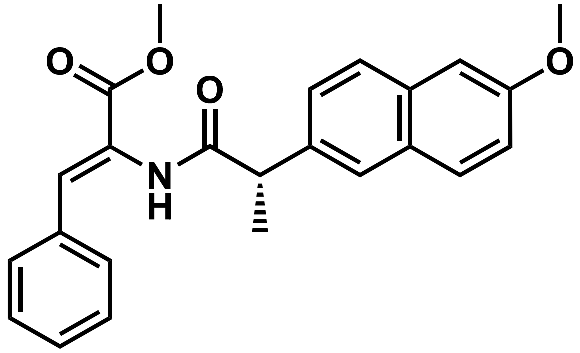

Following general procedure 5, methyl (*S,Z*)-2-(2-(6-methoxynaphthalen-2-yl)propanamido)-3-phenylacrylate (**3e**) was isolated using flash chromatography (0-60% EtOAc:Hex gradient) as a white solid (24.5 mg, 0.063 mmol, 25% yield). **^{1}H NMR** (600 MHz, $CDCl_3$) δ 7.79 (d, *J* = 8.4 Hz, 1H), 7.75 – 7.70 (m, 2H), 7.44 (d, *J* = 8.3 Hz, 1H), 7.22 – 7.11 (m, 4H), 7.05 (d, *J* = 7.6 Hz, 2H), 6.95 (t, *J* = 7.3 Hz, 3H), 3.94 (s, 3H), 3.87 (q, *J* = 7.0 Hz, 1H), 3.80 (s, 3H), 1.63 (d, *J* = 7.1 Hz, 3H). **^{13}C NMR** (151 MHz, $CDCl_3$) δ 172.3, 165.7, 157.9, 135.4, 134.0, 133.5, 131.2, 129.5, 129.4, 129.3, 129.1, 128.4, 127.9, 126.5, 126.4, 124.6, 119.5, 105.7, 55.5, 52.7, 47.2, 17.9. **HRMS** (ESI) m/z $[M+H]^+$ calcd for $C_{24}H_{23}NO_4^+$ 390.1700; Found 390.1694.

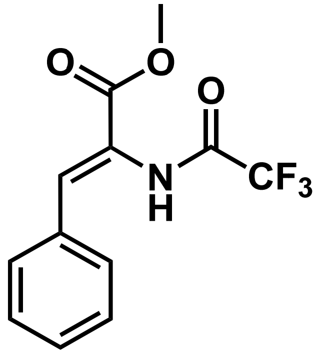

Following general procedure 5, methyl (*Z*)-3-phenyl-2-(2,2,2-trifluoroacetamido)acrylate (**3f**) was isolated using flash chromatography (0-60% EtOAc:Hex gradient) as a colorless solid (39.0 mg, 0.143 mmol, 57% yield). **^{1}H NMR** (400 MHz, $CDCl_3$) δ 7.76 (s, 1H), 7.63 (s, 1H), 7.44 – 7.37 (m, 5H), 3.89 (s, 3H). **^{13}C NMR** (126 MHz, $CDCl_3$) δ 164.5, 155.1 (q, *J* = 37.9 Hz), 135.7, 132.8, 130.5, 129.7, 129.0, 121.2, 115.7 (q, *J* = 288.3 Hz), 53.2. **^{19}F NMR** (376 MHz, $CDCl_3$) δ -75.7. **HRMS** (ESI) $[M+H]^+$ calcd for $C_{12}H_{11}F_3NO_3$ 274.0691; found 274.0668

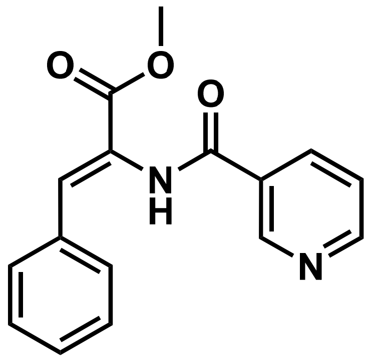

Following general procedure 5, methyl (*Z*)-2-(nicotinamido)-3-phenylacrylate (**3g**) was isolated using flash chromatography (0-60% EtOAc:Hex gradient) as a white solid (65.7 mg, 0.233 mmol, 93% yield). ^{1}H NMR (400 MHz, $CDCl_3$) δ 9.05 (s, 1H), 8.75 (dd, *J* = 4.9, 1.7 Hz, 1H), 8.20 – 8.14 (m, 1H), 7.92 (s, 1H), 7.52 (s, 1H), 7.48 (dd, *J* = 7.6, 2.1 Hz, 2H), 7.40 (dd, *J* = 8.0, 4.9 Hz, 1H), 7.37 – 7.28 (m, 3H), 3.87 (s, 3H). **^{13}C NMR** (126 MHz, CDCl3) δ 165.7, 164.1, 152.9, 148.4, 135.8, 133.7, 132.9, 129.9, 129.7, 129.5, 128.9, 123.9, 123.8, 53.0. HRMS (ESI) [M+H]$^+$calcd for $C_{16}H_{15}N_2O_3$ 283.1083; found 283.1080

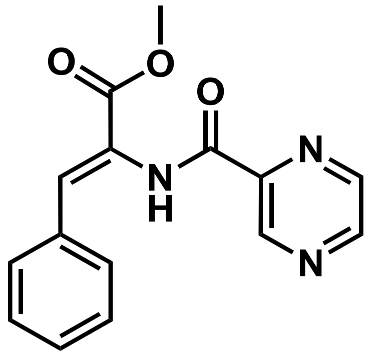

Following general procedure 5, methyl (*Z*)-3-phenyl-2-(pyrazine-2-carboxamido)acrylate (**3h**) was isolated using flash chromatography (0-60% EtOAc:Hex gradient) as a white solid (55.2 mg, 0.195 mmol, 78% yield). **^{1}H NMR** (400 MHz, $CDCl_3$) δ 9.44 – 9.38 (m, 2H), 8.79 (d, *J* = 2.4 Hz, 1H), 8.58 (dd, *J* = 2.3, 1.5 Hz, 1H), 7.53 – 7.47 (m, 3H), 7.37 – 7.30 (m, 3H), 3.87 (s, 3H). **^{13}C NMR** (101 MHz, $CDCl_3$) δ 165.4, 161.4, 147.8, 144.9, 143.9, 142.8, 133.7, 132.9, 129.8, 129.7, 128.8, 123.6, 52.9. **HRMS** (ESI) m/z [M+H]$^+$ calcd for $C_{15}H_{14}N_3O_3^+$ 284.1030; Found 284.1034.

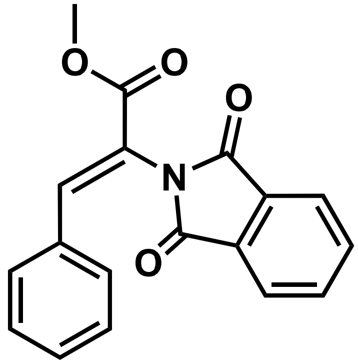

Synthesized according to general procedure 5, methyl (*Z*)-2-(1,3-dioxoisoindolin-2-yl)-3-phenylacrylate (**3i)** was isolated using flash chromatography (0-60% EtOAc:Hex gradient) as a white solid (53.4 mg, 0.174 mmol, 70% yield). **^{1}H NMR** (400 MHz, $CDCl_3$) δ 8.12 (s, 1H), 7.91 (dd, *J* = 5.4, 3.1 Hz, 2H), 7.78 (dd, *J* = 5.5, 3.2 Hz, 2H), 7.44 – 7.36 (m, 2H), 7.29 (q, *J* = 6.8 Hz, 3H), 3.82 (s, 3H). **^{13}C NMR** (151 MHz, $CDCl_3$) δ 166.9, 164.0, 143.1, 134.6, 132.4, 132.2, 130.7, 129.5, 129.0, 124.1, 120.1, 52.9. **HRMS** (ESI) m/z [M+H]$^+$ calcd for $C_{18}H_{14}NO_4^+$ 308.0917; found 308.0931.

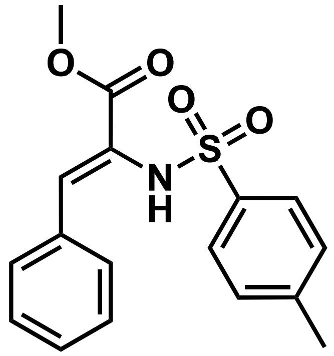

Synthesized according to general procedure 5, methyl (*Z*)-2-((4-methylphenyl)sulfonamido)-3-phenylacrylate (**3j)** was isolated using flash chromatography (0-40% EtOAc:Hex gradient) as a yellow solid (55.5 mg, 0.168 mmol, 67% yield). **^{1}H NMR** (600 MHz, $CDCl_3$) δ 7.89 – 7.76 (m, 2H), 7.66 (d, *J* = 8.1 Hz, 2H), 7.52 (s, 1H), 7.39 – 7.33 (m, 3H), 7.23 (d, *J* = 8.0 Hz, 2H), 6.19 (s, 1H), 3.53 (s, 3H), 2.39 (s, 3H). **^{13}C NMR** (151 MHz, $CDCl_3$) δ 165.5, 144.0, 137.8, 136.5, 132.9, 131.2, 130.5, 129.5, 128.6, 127.7, 122.9, 52.7, 21.7. **HRMS** (ESI) m/z $[M+H]^+$ calcd for $C_{17}H_{18}NO_4S^+$ 332.0951; found 332.0950.

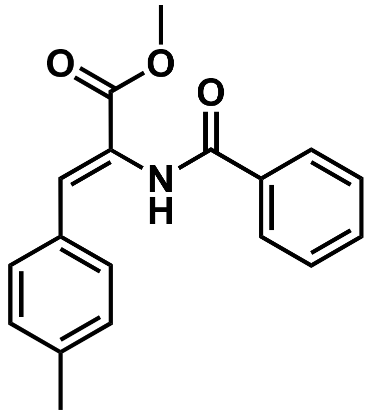

Following general procedure 5, methyl (*Z*)-2-benzamido-3-(*p*-tolyl)acrylate (**3k**) was isolated using flash chromatography (0-60% EtOAc:Hex gradient) as a white solid (43.7 mg, 0.148 mmol, 59% yield). **^{1}H NMR** (400 MHz, $CDCl_3$) δ 7.87 (d, *J* = 7.5 Hz, 2H), 7.81 (s, 1H), 7.59 – 7.51 (m, 1H), 7.49 – 7.43 (m, 3H), 7.40 (d, *J* = 8.0 Hz, 2H), 7.13 (d, *J* = 7.8 Hz, 2H), 3.84 (s, 3H), 2.32 (s, 3H). **^{13}C NMR** (101 MHz, $CDCl_3$) δ 166.1, 165.8, 140.0, 133.8, 132.4, 132.2, 131.1, 129.9, 129.5, 128.8, 127.6, 123.5, 52.8, 21.5. This data agrees with literature reports.[21]

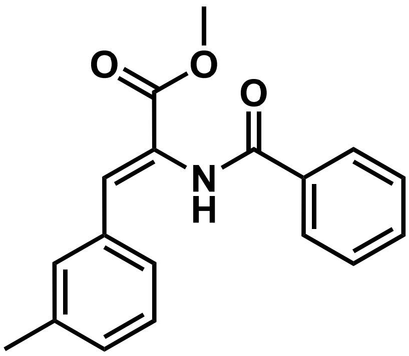

Following general procedure 5, methyl (*Z*)-2-benzamido-3-(*m*-tolyl)acrylate (**3l**) was isolated using flash chromatography (0-60% EtOAc:Hex gradient) as a white solid (26.0 mg, 0.088 mmol, 35% yield). **^{1}H NMR** (400 MHz, $CDCl_3$) δ 7.86 (d, *J* = 7.0 Hz, 2H), 7.73 (s, 1H), 7.55 (dd, *J* = 6.5, 1.3 Hz, 1H), 7.52 – 7.41 (m, 3H), 7.32 (d, *J* = 7.3 Hz, 2H), 7.22 (t, *J* = 7.6 Hz, 1H), 7.12 (d, *J* = 7.6 Hz, 1H), 3.85 (s, 3H), 2.29 (s, 3H). **^{13}C NMR** (101 MHz, $CDCl_3$) δ 166.0, 138.3, 133.9, 133.8, 132.3, 132.1, 130.7, 130.5, 128.9, 128.7, 127.6, 126.7, 124.3, 52.9, 21.5. One carbon is missing due to overlapping. **HRMS** (ESI) m/z $[M+H]^+$ calcd $C_{18}H_{17}NNaO_3^+$ 318.1101; Found 318.1106.

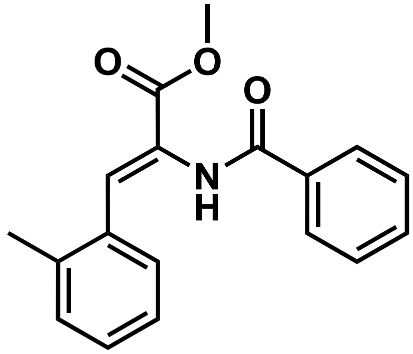

Following general procedure 5, methyl (*Z*)-2-benzamido-3-(*o*-tolyl)acrylate (**3m**) was isolated using flash chromatography (0-60% EtOAc:Hex gradient) as a white solid (11.8 mg, 0.040 mmol, 16% yield). ^{1}H NMR (400 MHz, CDCl3) δ 7.78 (d, *J* = 7.8 Hz, 2H), 7.59 (s, 1H), 7.56 – 7.50 (m, 1H), 7.48 (s, 1H), 7.46 – 7.38 (m, 3H), 7.25 – 7.17 (m, 2H), 7.14 – 7.08 (m, 1H), 3.89 (s, 3H), 2.38 (s, 3H). **^{13}C NMR** (126 MHz, $CDCl_3$) δ 165.8, 137.5, 133.7, 133.2, 132.3, 130.7, 129.2, 129.2, 128.8, 127.9, 127.6, 126.1, 125.9, 52.9, 20.2. One carbon is missing due to overlapping. This data agrees with literature reports.[21]

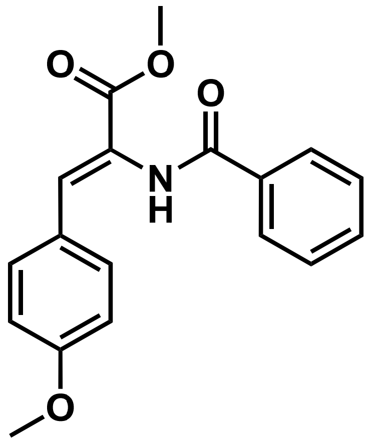

Following general procedure 5, methyl (*Z*)-2-benzamido-3-(4-methoxyphenyl)acrylate (**3n**) was isolated using flash chromatography (0-60% EtOAc:Hex gradient) as a white solid (44.5 mg, 0.143 mmol, 57% yield). **^{1}H NMR** (400 MHz, $CDCl_3$) δ 7.88 (d, *J* = 7.6 Hz, 2H), 7.81 (bs, 1H), 7.55 (t, *J* = 7.4 Hz, 1H), 7.52 – 7.39 (m, 5H), 6.83 (d, *J* = 8.8 Hz, 2H), 3.82 (s, 3H), 3.78 (s, 3H). **^{13}C NMR** (101 MHz, $CDCl_3$) δ 166.2, 165.5, 160.7, 133.8, 132.7, 132.2, 131.9, 128.8, 127.6, 126.5, 122.0, 114.2, 55.4, 52.7. This data agrees with literature reports.[21]

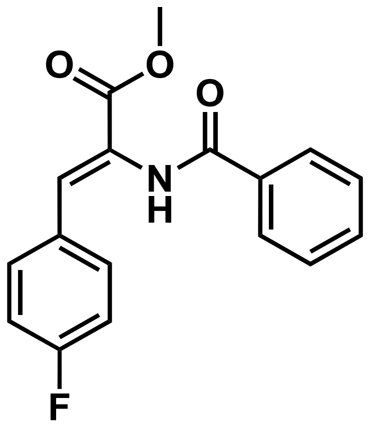

Following general procedure 5, methyl (*Z*)-2-benzamido-3-(4-fluorophenyl)acrylate (**3o**) was isolated using flash chromatography (0-60% EtOAc:Hex gradient) as a off-white solid (35.3 mg, 0.118 mmol, 47% yield). **^{1}H NMR** (400 MHz, $CDCl_3$) δ 7.87 (d, *J* = 7.8 Hz, 2H), 7.80 (s, 1H), 7.57 (t, *J* = 7.0 Hz, 1H), 7.53 – 7.44 (m, 5H), 7.01 (t, *J* = 8.6 Hz, 2H), 3.87 (s, 3H). **^{13}C NMR** (126 MHz, $CDCl_3$) δ 166.0, 165.6, 163.1 (d, *J* = 251.3 Hz), 133.7, 132.5, 131.9 (d, *J* = 8.4 Hz), 130.8, 130.3 (d, *J* = 3.4 Hz), 129.0, 127.6, 123.5, 115.9 (d, *J* = 21.7 Hz), 53.0.. **^{19}F NMR** (376 MHz, $CDCl_3$) δ -110.0. This data agrees with literature reports.[21]

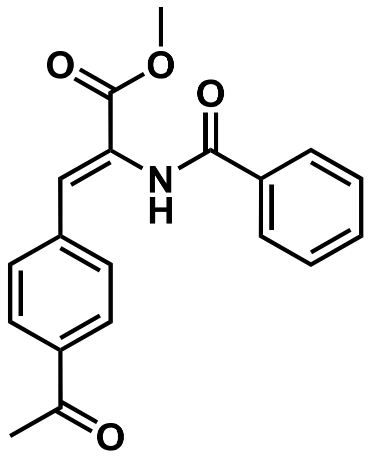

Following general procedure 5, methyl (*Z*)-3-(4-acetylphenyl)-2-benzamidoacrylate (**3p**) was isolated using flash chromatography (0-60% EtOAc:Hex gradient) as a off-white solid (26.8 mg, 0.083 mmol, 33% yield). **^{1}H NMR** (400 MHz, $CDCl_3$) δ 7.96 (s, 1H), 7.90 (d, *J* = 8.1 Hz, 2H), 7.86 (d, *J* = 7.7 Hz, 2H), 7.60 – 7.53 (m, 3H), 7.52 – 7.45 (m, 3H), 3.90 (s, 3H), 2.56 (s, 3H). **^{13}C NMR** (126 MHz, $CDCl_3$) δ 197.6, 165.8, 165.2, 139.1, 137.0, 133.5, 132.6, 129.7, 129.1, 129.0, 128.6, 127.6, 125.2, 53.2, 26.8. **HRMS** (ESI) m/z $[M+Na]^+$ calcd for $C_{19}H_{17}NNaO_4^+$ 346.1050; found 346.1050.

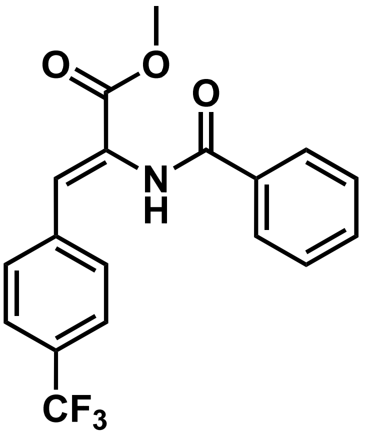

Following general procedure 5, methyl (*Z*)-2-benzamido-3-(4-(trifluoromethyl)phenyl)acrylate (**3q**) was isolated using flash chromatography (0-60% EtOAc:Hex gradient) as a yellow solid (25.5 mg, 0.073 mmol, 29% yield). **^{1}H NMR** (400 MHz, $CDCl_3$) δ 7.94 (s, 1H), 7.85 (d, *J* = 7.5 Hz, 2H), 7.63 – 7.54 (m, 5H), 7.53 – 7.43 (m, 3H), 3.90 (s, 3H). **^{13}C NMR** (126 MHz, $CDCl_3$) δ 165.7, 165.3, 137.9, 133.5, 132.6, 130.8 (q, *J* = 32.7 Hz), 129.8, 129.1, 129.0, 127.6, 125.5 (d, *J* = 3.8 Hz), 125.3, 122.9 (q, *J* = 272.2 Hz), 53.2. **^{19}F NMR** (376 MHz, $CDCl_3$) δ -62.9 (s, 3F). **HRMS** (ESI) m/z $[M+H]^+$ calcd for $C_{18}H_{15}F_3NO_3^+$ 350.0999; found 350.1010.

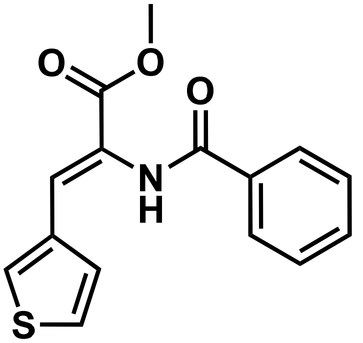

Following general procedure 5, methyl (*Z*)-2-benzamido-3-(thiophen-3-yl)acrylate (**3r**) was isolated using flash chromatography (0-60% EtOAc:Hex gradient) as a yellow solid (25.8 mg, 0.090 mmol, 36% yield). **^{1}H NMR** (400 MHz, $CDCl_3$) δ 7.90 (d, *J* = 7.6 Hz, 2H), 7.72 (s, 1H), 7.61 – 7.53 (m, 3H), 7.48 (t, *J* = 7.6 Hz, 2H), 7.28 – 7.25 (m, 2H), 3.83 (s, 3H). **^{13}C NMR** (126 MHz, $CDCl_3$) δ 166.1, 166.1, 135.3, 133.7, 132.4, 129.6, 128.9, 128.0, 127.7, 127.6, 126.3, 122.6, 52.8. **HRMS** (ESI) m/z $[M+H]^+$ calcd for $C_{15}H_{14}NO_3S^+$ 288.0689; found 288.0690.

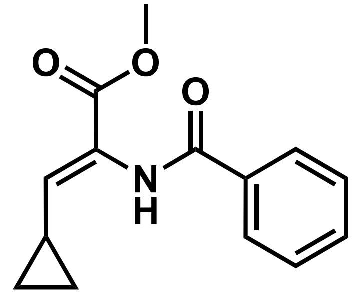

Modified from general procedure 5, methyl (*Z*)-2-benzamido-3-cyclopropylacrylate (**3s**) was isolated using flash chromatography (0-60% EtOAc:Hex gradient) as a colorless solid (40.4 mg, 0.165 mmol, 66% yield) using 20 mol% of K_2CO_3 as an additive. **^{1}H NMR** (500 MHz, $CDCl_3$) δ 7.89 (d, *J* = 7.4 Hz, 2H), 7.60 – 7.42 (m, 4H), 6.23 (d, *J* = 10.8 Hz, 1H), 3.77 (s, 3H), 1.75 – 1.54 (m, 1H), 1.06 (td, *J* = 6.8, 4.4 Hz, 2H), 0.73 (dt, *J* = 6.7, 4.4 Hz, 2H). **^{13}C NMR** (126 MHz, $CDCl_3$) δ 166.0, 165.2, 144.3, 134.3, 132.1, 128.8, 127.5, 52.5, 31.2, 12.7, 9.1. **HRMS** (ESI) m/z $[M+H]^+$ calcd for $C_{14}H_{16}NO_3$ 246.1125; found 246.1139.

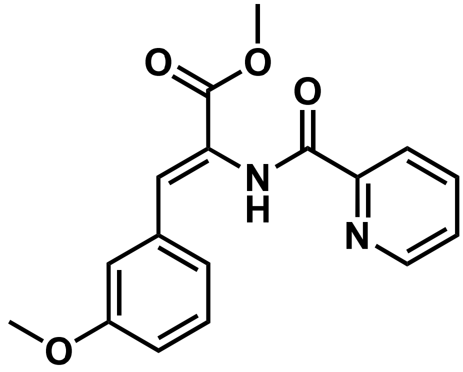

Following general procedure 5, methyl (*Z*)-3-(3-methoxyphenyl)-2-(picolinamido)acrylate (**3t**) was isolated using flash chromatography (0-60% EtOAc:Hex gradient) as a colorless oil (53.1 mg, 0.170 mmol, 68% yield). **^{1}H NMR** (400 MHz, $CDCl_3$) δ 9.74 (s, 1H), 8.63 (d, *J* = 4.6 Hz, 1H), 8.24 (d, *J* = 7.8 Hz, 1H), 7.90 (td, *J* = 7.7, 1.7 Hz, 1H), 7.50 (ddd, *J* = 7.6, 4.8, 1.1 Hz, 1H), 7.43 (s, 1H), 7.30 – 7.27 (m, 1H), 7.19 – 7.11 (m, 2H), 6.90 (dd, *J* = 7.9, 2.2 Hz, 1H), 3.90 (s, 3H), 3.72 (s, 3H). **^{13}C NMR** (126 MHz, $CDCl_3$) δ 165.6, 162.7, 159.6, 149.1, 148.4, 137.7, 135.0, 131.6, 129.7, 126.8, 124.6, 122.8, 122.6, 115.8, 114.4, 55.2, 52.8. This data agrees with literature reports.[22]

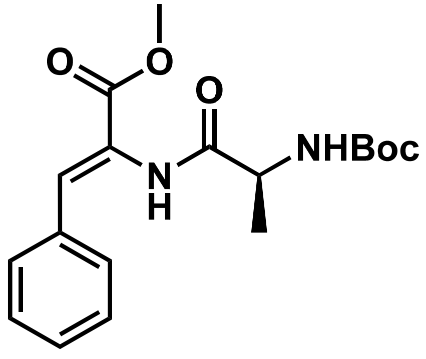

Following general procedure 5, methyl (S,*Z*)-2-(2-((*tert*-butoxycarbonyl)amino)propanamido)-3-phenylacrylate (**3u**) was isolated using flash chromatography (0-60% EtOAc:Hex gradient) as a white solid (46.3 mg, 0.133 mmol, 53% yield). **^{1}H NMR** (400 MHz, $CDCl_3$) δ 7.70 (s, 1H), 7.48 (d, *J* = 7.1 Hz, 2H), 7.43 (s, 1H), 7.39 – 7.29 (m, 3H), 4.97 (s, 1H), 4.36 – 4.24 (m, 1H), 3.84 (s, 3H), 1.46 (s, 9H), 1.42 (d, *J* = 7.1 Hz, 3H). **^{13}C NMR** (126 MHz, $CDCl_3$) δ 171.5, 165.6, 155.8, 133.7, 133.2, 129.9, 129.6, 128.7, 123.9, 80.4, 52.8, 50.5, 28.4, 17.9. This data agrees with literature reports.[23]

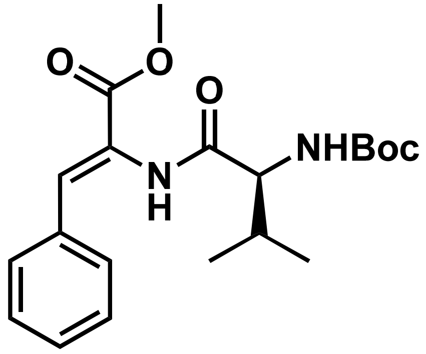

Following general procedure 5, methyl (*S*,*Z*)-2-(2-((*tert*-butoxycarbonyl)amino)-3-methylbutanamido)-3-phenylacrylate (**3v**) was isolated using flash chromatography (0-60% EtOAc:Hex gradient) as a white solid (48.2 mg, 0.128 mmol, 51% yield). **^{1}H NMR** (400 MHz, $CDCl_3$) δ 7.72 (s, 1H), 7.46 (d, *J* = 7.2 Hz, 2H), 7.37 (s, 1H), 7.32 (m, 3H), 5.08 (d, *J* = 8.9 Hz, 1H), 4.10 (t, *J* = 7.3 Hz, 1H), 3.81 (s, 3H), 2.23 (h, *J* = 6.7 Hz, 1H), 1.44 (s, 9H), 0.99 (dd, *J* = 22.1, 6.8 Hz, 6H). **^{13}C NMR** (101 MHz, $CDCl_3$) δ 170.8, 165.6, 156.2, 133.7, 132.8, 129.9, 129.6, 128.7, 124.1, 80.3, 60.3, 52.7, 30.6, 28.4, 19.4, 17.7. This data agrees with literature reports.[23]

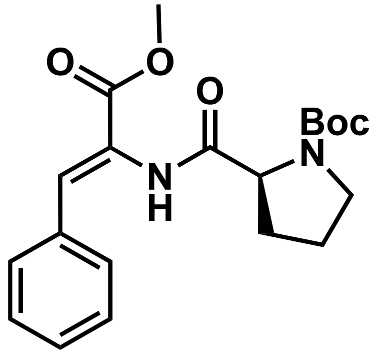

Following general procedure 5, *tert*-butyl (*S*,*Z*)-2-((3-methoxy-3-oxo-1-phenylprop-1-en-2-yl)carbamoyl)pyrrolidine-1-carboxylate (**3w**) was isolated using flash chromatography (0-60% EtOAc:Hex gradient) as a white solid (61.7 mg, 0.165 mmol, 66% yield). **^{1}H NMR** (400 MHz, CD_3OD) δ 7.70 (d, *J* = 6.0 Hz, 1H), 7.58 (s, 1H), 7.45 (d, *J* = 6.4 Hz, 1H), 7.38 (d, *J* = 7.3 Hz, 2H), 4.38 (t, *J* = 7.9 Hz, 1H), 3.80 (s, 3H), 3.53 (s, 1H), 3.46 – 3.35 (m, 1H), 2.35 – 2.18 (m, 1H), 2.17 – 2.09 (m, 1H), 2.04 – 1.84 (m, 2H), 1.47 (appd, *J* = 18.2 Hz, 9H). ^{13}C NMR (101 MHz, CD_3OD) δ 175.8*, 175.1*, 167.0,* 166.8,* 136.1,* 135.1,* 134.9,* 134.6,* 131.6,* 130.9,* 130.8,* 130.7,* 129.7, 126.6,* 126.1,* 81.7, 81.3, 47.9, 32.2,* 31.1,* 28.8,* 28.7,* 25.3,* 24.3.* **HRMS** (ESI) m/z $[M+H]^+$ calcd for $C_{20}H_{26}N_2NaO_5^+$ 397.1734; found 397.1734. *Note:* This compound exists as a mixture of rotamers. In $CDCl_3$ all ^{1}H NMR peaks are broadened and some ^{13}C NMR peaks are broadened. In CD_3OD two distinct rotamers are observed. "*" indicates a ^{13}C NMR peak that has an observed rotamer peak present in the spectra.

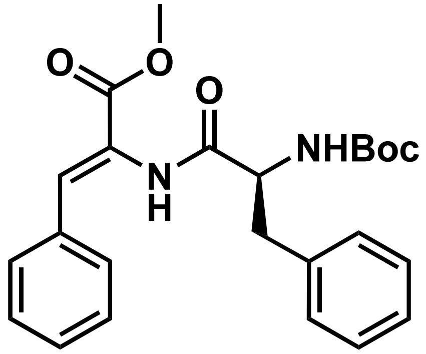

Following general procedure 5, methyl (*S*,*Z*)-2-(2-((*tert*-butoxycarbonyl)amino)-3-phenylpropanamido)-3-phenylacrylate (**3x**) was isolated using flash chromatography (0-60% EtOAc:Hex gradient) as a white solid (58.5 mg, 0.138 mmol, 55% yield). **^{1}H NMR** (500 MHz, $CDCl_3$) δ 7.60 (s, 1H), 7.37 (m, 3H), 7.34 – 7.29 (m, 5H), 7.27 (s, 1H), 7.24 (s, 1H), 4.93 (s, 1H), 4.50 (q, *J* = 7.7 Hz, 1H), 3.20 (dd, *J* = 14.0, 6.6 Hz, 1H), 3.08 (dd, *J* = 14.2, 7.2 Hz, 1H), 1.41 (s, 9H). **^{13}C NMR** (126 MHz, $CDCl_3$) δ 170.2, 165.5, 155.8, 136.5, 133.6, 132.7, 129.8, 129.6, 129.5, 128.9, 128.8, 127.2, 123.9, 80.7, 52.8, 37.5, 28.4. This data agrees with literature reports.[23]

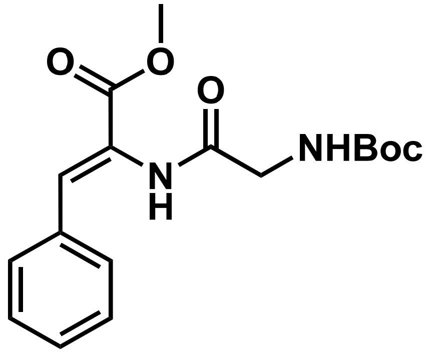

Modified from general procedure 5, methyl (*Z*)-2-(2-((*tert*-butoxycarbonyl)amino)acetamido)-3-phenylacrylate (**3y**) was isolated using flash chromatography (0-60% EtOAc:Hex gradient) as a colorless oil (36.1 mg, 0.108 mmol, 43% yield) using 90 °C instead of 40 °C. **^{1}H NMR** (400 MHz, $CDCl_3$) δ 7.77 (s, 1H), 7.53 – 7.28 (m, 6H), 5.28 (s, 1H), 3.92 (s, 2H), 3.83 (s, 3H), 1.44 (s, 9H). **^{13}C NMR** (151 MHz, $CDCl_3$) δ 168.6, 165.6, 156.3, 133.5, 133.3, 129.9, 129.8, 128.8, 123.6, 80.6, 52.9, 44.9, 28.4. ^{13}C NMR (151 MHz, $CDCl_3$) δ 168.6, 165.6, 156.3, 133.5, 133.3, 129.9, 129.8, 128.8, 123.6, 80.6, 52.9, 44.9, 28.4. This data agrees with literature reports.[24]

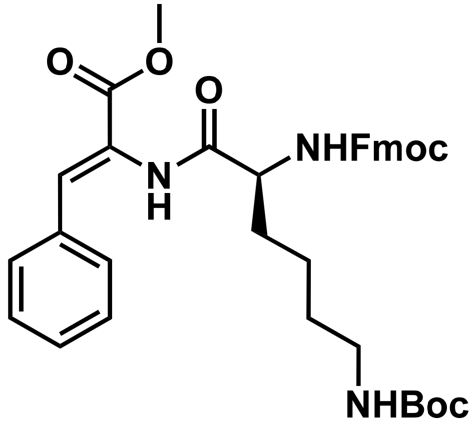

Following general procedure 5, methyl (*S*,*Z*)-2-(2-((((9H-fluoren-9-yl)methoxy)carbonyl)amino)-6-((*tert*-butoxycarbonyl)amino)hexanamido)-3-phenylacrylate (**3z**) was isolated using flash chromatography (20-100% EtOAc:Hex gradient) as a white solid (22.0 mg, 0.035 mmol, 14% yield). **^{1}H NMR** (400 MHz, CD_3OD) δ 7.79 (d, *J* = 7.6 Hz, 2H), 7.68 (d, *J* = 7.4 Hz, 2H), 7.61 (d, *J* = 6.7 Hz, 2H), 7.45 (s, 1H), 7.43 – 7.20 (m, 9H), 6.55 (bs, 1H), 4.48 – 4.37 (m, 2H), 4.23 (m, 2H), 3.79 (s, 3H), 3.06 (t, *J* = 5.8 Hz, 2H), 1.94 – 1.80 (m, 1H), 1.77 – 1.64 (m, 1H), 1.55 – 1.46 (m, 4H), 1.42 (s, 9H). **^{13}C NMR** (126 MHz, $CDCl_3$) δ 170.6, 165.4, 156.5, 156.3, 143.7, 143.7, 141.3, 133.4, 133.2, 129.7, 128.6, 127.8, 127.1, 125.1, 123.8, 120.0, 79.2, 67.2, 55.0, 52.7, 47.1, 39.7, 31.4, 29.6, 28.4, 22.2. **HRMS** (ESI) m/z $[M+H]^+$ calcd for $C_{36}H_{42}N_3O_7^+$ 628.3017; found 628.3039.

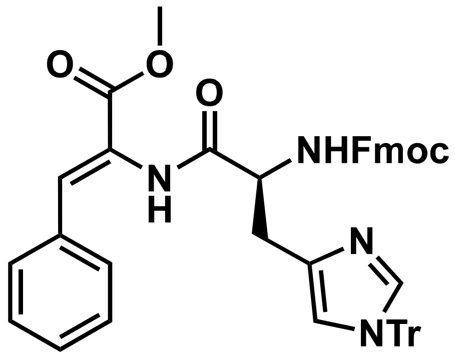

Following general procedure 5, methyl (*S*,*Z*)-2-(2-((((9H-fluoren-9-yl)methoxy)carbonyl)amino)-3-(1-trityl-*1H*-imidazol-4-yl)propanamido)-3-phenylacrylate (**3aa**) was isolated using flash chromatography (20-100% EtOAc:Hex gradient) as a white solid (37.0 mg, 0.0475 mmol, 19% yield). **^{1}H NMR** (400 MHz, MeOD) δ 7.78 (d, *J* = 7.3 Hz, 2H), 7.65 – 7.55 (m, 3H), 7.45 (s, 1H), 7.41 – 7.16 (m, 20H), 7.08 (dd, *J* = 6.5, 2.9 Hz, 6H), 6.85 (d, *J* = 4.6 Hz, 1H), 4.56 (dd, *J* = 9.1, 4.8 Hz, 1H), 4.31 (t, *J* = 7.4 Hz, 2H), 4.14 (t, *J* = 6.7 Hz, 1H), 3.71 (s, 3H), 3.13 (dd, *J* = 14.7, 4.6 Hz, 1H), 2.91 (dd, *J* = 14.4, 9.7 Hz, 1H). **^{13}C NMR** (126 MHz, MeOD) δ 173.9, 166.9, 158.4, 145.2, 143.6, 142.6, 139.4, 137.6, 135.6, 134.6, 131.3, 130.9, 130.8, 129.8, 129.3, 129.2, 128.8, 128.2, 126.3, 120.9, 76.9, 68.2, 56.6, 52.9, 37.6, 31.4. **HRMS** (ESI) m/z $[M+H]^+$ calcd for $C_{50}H_{43}N_4O_5^+$ 779.3228; found 779.3229.

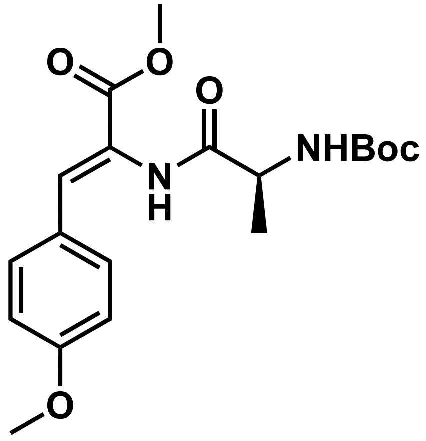

Following general procedure 5, methyl (*S*,*Z*)-2-(2-((*tert*-butoxycarbonyl)amino)propanamido)-3-(4-methoxyphenyl)acrylate (**3ab**) was isolated using flash chromatography (0-60% EtOAc:Hex gradient) as a white solid (69.2 mg, 0.183 mmol, 73% yield). **^{1}H NMR** (500 MHz, $CDCl_3$) δ 7.92 (s, 1H), 7.45 (d, *J* = 8.4 Hz, 2H), 7.39 (s, 1H), 6.83 (d, *J* = 8.6 Hz, 2H), 5.27 (d, *J* = 7.3 Hz, 1H), 4.43 – 4.33 (m, 1H), 3.77 (s, 3H), 3.76 (s, 3H), 1.51 – 1.33 (m, 12H). **^{13}C NMR** (126 MHz, $CDCl_3$) δ 171.8, 165.9, 160.7, 155.8, 134.0, 132.0, 126.1, 121.6, 114.1, 80.3, 55.3, 52.6, 50.4, 28.4, 18.1. **HRMS** (ESI) m/z $[M+H]^+$ calcd $C_{19}H_{27}N_2O_6^+$ for 379.1864; found 379.1857.

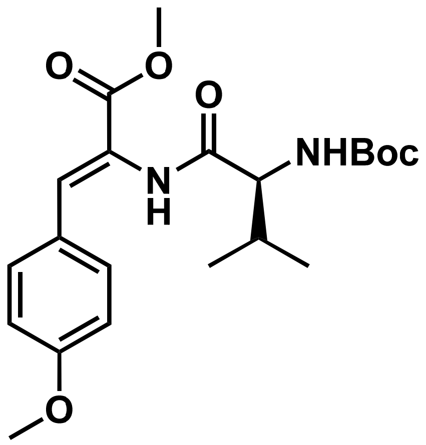

Following general procedure 5, methyl (*S*,*Z*)-2-(2-((*tert*-butoxycarbonyl)amino)-3-methylbutanamido)-3-(4-methoxyphenyl)acrylate (**3ac**) was isolated using flash chromatography (0-60% EtOAc:Hex gradient) as a white solid (54.8 mg, 0.135 mmol, 54% yield). **^{1}H NMR** (400 MHz, $CDCl_3$) δ 7.69 (s, 1H), 7.45 (d, *J* = 8.4 Hz, 2H), 7.38 (s, 1H), 6.83 (d, *J* = 8.8 Hz, 2H), 5.11 (d, *J* = 8.8 Hz, 1H), 4.18 – 4.10 (m, 1H), 3.79 – 3.78 (appd, 6H), 2.25 (h, *J* = 5.8 Hz, 1H), 1.44 (s, 9H), 1.01 (dd, *J* = 22.3, 6.8 Hz, 6H). **^{13}C NMR** (101 MHz, $CDCl_3$) δ 170.9, 165.8, 160.8, 156.2, 133.6, 132.0, 126.1, 121.8, 114.2, 80.3, 60.3, 55.4, 52.5, 30.7, 28.5, 19.5. **HRMS** (ESI) m/z $[M+H]^+$ calcd for $C_{21}H_{31}N_2O_6^+$ 407.2177; found 407.2180.

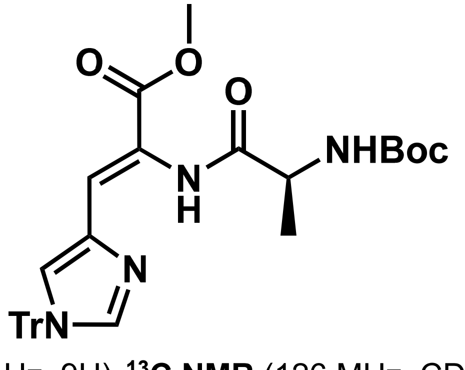

Following general procedure 5, methyl (*S*,*Z*)-2-(2-((*tert*-butoxycarbonyl)amino)propanamido)-3-(1-trityl-*1H*-imidazol-4-yl)acrylate (**3ad**) was isolated using flash chromatography (0-60% EtOAc:Hex gradient) as a white solid (107.4 mg, 0.185 mmol, 74% yield). **^{1}H NMR** (400 MHz, $CDCl_3$) δ 10.67 (s, 1H), 7.47 (s, 1H), 7.39 – 7.33 (m, 9H), 7.11 (dd, *J* = 6.3, 2.9 Hz, 6H), 6.91 (s, 1H), 6.49 (s, 1H), 5.30 (s, 1H), 4.40 (s, 1H), 3.80 (s, 3H), 1.45 (d, *J* = 6.9 Hz, 3H), 1.39 (d, *J* = 1.3 Hz, 9H). **^{13}C NMR** (126 MHz, $CDCl_3$) δ 171.3, 165.6, 155.3, 141.9, 139.0, 136.8, 129.8, 128.5, 128.4, 127.0, 123.4, 113.4, 79.6, 75.9, 52.4 50.4, 28.5, 19.5. **HRMS** (ESI) m/z $[M-Boc+2H]^+$ calcd for $C_{29}H_{29}N_4O_3^+$ 481.2234; found 481.2233.

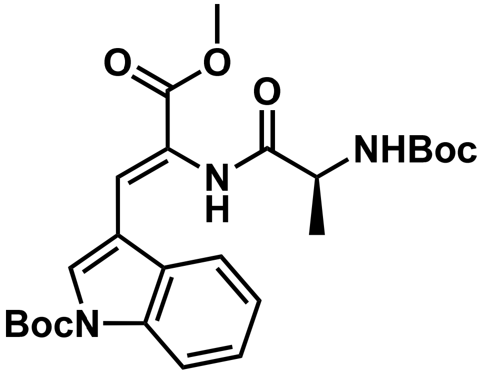

Following general procedure 5, *tert*-butyl (*S,Z*)-3-(2-(2-((*tert*-butoxycarbonyl)amino)propanamido)-3-methoxy-3-oxoprop-1-en-1-yl)-*1H*-indole-1-carboxylate (**3ae**) was isolated using flash chromatography (0-60% EtOAc:Hex gradient) as a colorless solid (107.2 mg, 0.220 mmol, 88% yield). **^{1}H NMR** (400 MHz, $CDCl_3$) δ 8.13 (d, *J* = 8.1 Hz, 1H), 7.90 (s, 1H), 7.85 (s, 1H), 7.73 (s, 1H), 4.38 (apt, *J* = 6.4 Hz, 1H), 7.38 – 7.27 (m, 2H), 5.08 (s, 1H), 4.38 (d, *J* = 6.5 Hz, 1H), 3.85 (s, 3H), 1.67 (s, 9H), 1.47 (d, *J* = 6.7 Hz, 3H), 1.44 (s, 9H). **^{13}C NMR** (101 MHz, $CDCl_3$) δ 171.3, 171.0, 165.5, 155.8, 149.4, 135.0, 129.6, 127.9, 125.2, 123.3, 122.8, 119.0, 115.5, 114.1, 84.7, 60.5, 52.7, 28.4, 28.3, 21.2, 14.3. **HRMS** (ESI) m/z $[M+H]^+$ calcd for $C_{25}H_{34}N_3O_7^+$ 488.2391; found 488.2391.

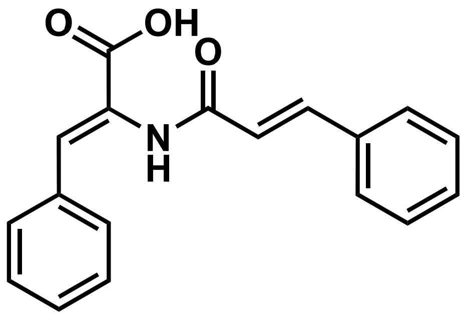

To a 0.25 M solution of **3b** (0.411 mmol, 126.0 mg, 1.0 equiv) in THF, powdered sodium hydroxide (2.47 mmol, 59.1 mg, 6.0 equiv) was added. One drop of water was also added to solubilize the sodium hydroxide. The reaction was allowed to stir at 40 °C, overnight. Then, 1 M aqueous HCl (4.11 mmol, 4.11 mL, 10.0 equiv) was added and a white precipitate formed. Compound **4** was extracted with ethyl acetate three times, dried over Na_2SO_4 and dried under vacuum. Upon removal of solvent a white solid remained (118 mg, 0.407 mmol, 99% yield). (*Z*)-2-cinnamamido-3-phenylacrylic acid (**4**) **^{1}H NMR** (400 MHz, DMSO-d_6) δ 12.71 (s, 1H), 9.74 (s, 1H), 7.62 (d, *J* = 7.0 Hz, 4H), 7.52 (d, *J* = 15.8 Hz, 1H), 7.41 (m, 6H), 7.28 (s, 1H), 6.86 (d, *J* = 15.8 Hz, 1H). **^{13}C NMR** (101 MHz, DMSO-d_6) δ 166.3, 164.5, 140.4, 134.6, 133.8, 131.1, 129.8, 129.7, 129.2, 129.0, 128.6, 127.8, 127.1, 121.1. **HRMS** (ESI) m/z $[M-H]^-$ calcd for $C_{18}H_{14}NO_3^-$ 292.0979; found 292.0978.

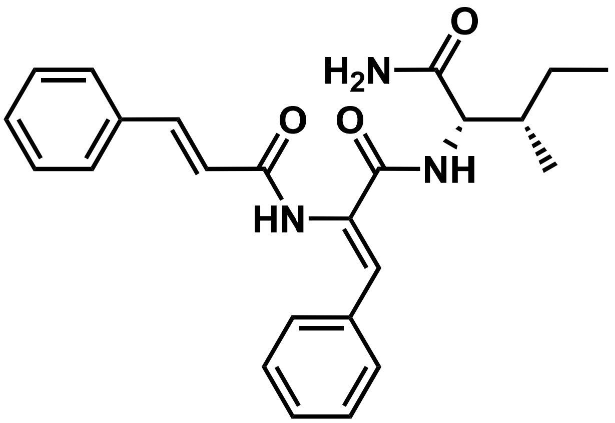

To a 0.25 M solution of compound **4** in DMF, O-(1H-6-Chlorobenzotriazole-1-yl)-1,1,3,3-tetramethyluronium hexafluorophosphate(HCTU) (0.150 mmol, 62.1 mg, 1.2 equiv) and *N*,*N*-diispropylethylamine (0.225 mmol, 39.2 µL, 1.8 equiv) were added. The mixture was allowed to stir at room temperature for 1 hour. Then, *L*-isoleucinamide (0.150 mmol, 19.5 mg, 1.2 equiv) was added and the reaction was allowed to stir overnight. Next, the reaction mixture was extracted with aqueous lithium bromide and ethyl acetate, three times each. The organic layer was dried over Na_2SO_4 and filtered. Celite was added and volatiles were removed under vacuum. The crude reaction was purified using flash chromatography with EtOAc:Hex (20-100%) gradient to afford Scutiamine M as a white solid (43.6 mg, 0.108 mmol, 86% yield). **^{1}H NMR** (400 MHz, DMSO-d_6) δ 9.85 (s, 1H), 7.71 (d, *J* = 8.6 Hz, 1H), 7.63 (d, *J* = 6.6 Hz, 2H), 7.57 (d, *J* = 7.7 Hz, 2H), 7.53 (d, *J* = 16.0 Hz, 1H), 7.48 – 7.29 (m, 7H), 7.12 (s, 1H), 7.01 (s, 1H), 6.90 (d, *J* = 15.8 Hz, 1H), 4.22 (dd, *J* = 8.5, 6.6 Hz, 1H), 1.88 – 1.78 (m, 1H), 1.50 – 1.41 (m, 1H), 1.18 – 1.03 (m, 1H), 0.88 (d, *J* = 6.8 Hz, 3H), 0.83 (t, *J* = 7.3 Hz, 3H). **^{13}C NMR** (126 MHz, DMSO) δ 173.0, 165.0, 164.9, 140.5, 134.6, 134.1, 130.1, 130.0, 129.5, 129.1, 128.7, 127.9, 127.6, 127.0, 121.0, 57.4, 36.6, 24.4, 15.7, 11.4. **HRMS** (ESI) m/z $[M+H]^+$ calcd for $C_{24}H_{28}N_3O_3^+$ 406.2125; found 406.2105. $[\alpha]^{27}_D$ -55 (*c* = 0.46, MeOH).

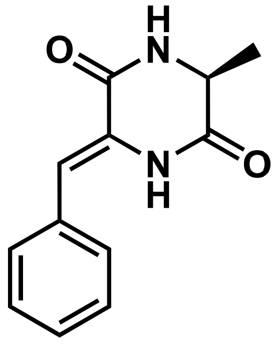

To a 0.25 M solution of **3u** in DCM, 4.0 M HCl in 1,3-dioxane (0.625 mmol, 208 µL, 5.0 equiv) was added and the reaction was allowed to stir at room temperature until TLC indicated consumption of **3s** (4 hours). Then, triethylamine (0.375 mmol, 52.3 µL, 3.0 equiv) was added and a white precipitate formed immediately. The reaction was allowed to stir overnight. Next, celite was added and volatiles were removed under vacuum. (S,Z)-3-benzylidene-6-methylpiperazine-2,5-dione (**5**) was isolated using flash chromatography with MeOH:DCM (10%) as the eluent, as a white solid (24.3 mg, 0.113 mmol, 90% yield). **^{1}H NMR** (600 MHz, DMSO-d_6) δ 9.93 (s, 1H), 8.48 (s, 1H), 7.49 (d, *J* = 7.7 Hz, 2H), 7.40 (t, *J* = 7.7 Hz, 2H), 7.30 (t, *J* = 7.3 Hz, 1H), 6.67 (s, 1H), 4.14 (qd, *J* = 7.1, 1.7 Hz, 1H), 1.34 (d, *J* = 6.9 Hz, 3H). **^{13}C NMR** (151 MHz, DMSO) δ 167.7, 160.4, 133.5, 129.3, 128.7, 128.0, 127.0, 114.1, 50.3, 19.3. **HRMS** (ESI) m/z $[M+H]^+$ calcd for $C_{12}H_{13}N_2O_2^+$ 217.0972; found 217.0980. $[\alpha]^{27}_D$ +4 (*c* = 1.9, DMSO).

6. Mechanistic Study

To evaluate whether the tri-*n*-butyl phosphine catalyst could isomerize the α,β-dehydroamino acid products both isomers were required. To gain access to the (*E*)-isomer, a photocatalyzed isomerization reaction was utilized.

Photocatalyzed isomerization of α,β-dehydroamino acid **3a**

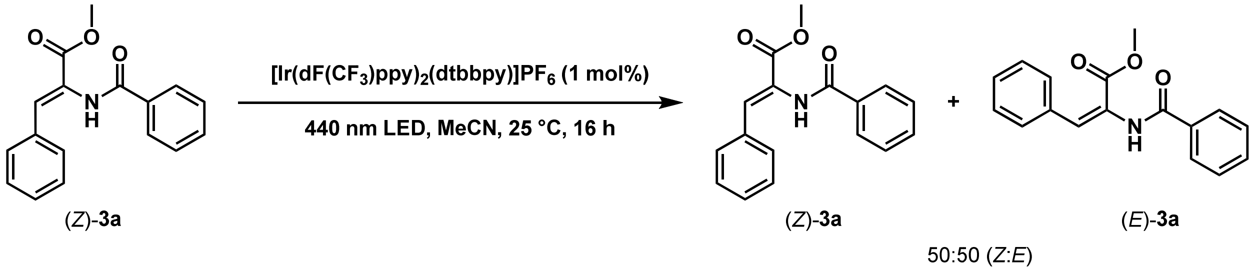

Scheme S7: Photocatalyzed isomerization of α,β-dehydroamino acid **3a.**

Procedure 7: To a flame dried 2 dram vial, a stir bar, [4,4′-*Bis*(1,1-dimethylethyl)-2,2′-bipyridine-*N*1,*N*1′]*bis*[3,5-difluoro-2-[5-(trifluoromethyl)-2-pyridinyl-*N*]phenyl-C]Iridium(III) hexafluorophosphate (3.4 mg, 0.003 mmol, 1 mol%) and (*Z*)-**3a** (93.1 mg, 331 mmol, 1.0 equiv) were added. The vial was put under inert atmosphere using standard *Schlenk* technique. Then dry MeCN (9.9 mL, 0.33 M) was added, and the vial was put in a Kessel photochemical reactor (PR160 Rig w/ Fan Kit) with a stir plate. The reactor includes four 440nm (max 45W) LED light fixtures (approximately 5 cm from reaction vial). The mixture was irradiated for 16 hours. After 16 hours, the reaction was diluted with 2 mL of EtOAc and TLC analysis was performed. (*Z*)-**3a** and (*E*)-**3a** were separated using flash chromatography (0-40% EtOAc:Hex gradient). (*Z*)-**3a** was obtained in a 23% yield (21.0 mg, 0.331 mmol) as a white solid. (*E*)-**3a** was obtained in a 23% yield (21.0 mg, 0.331 mmol) as a white solid.

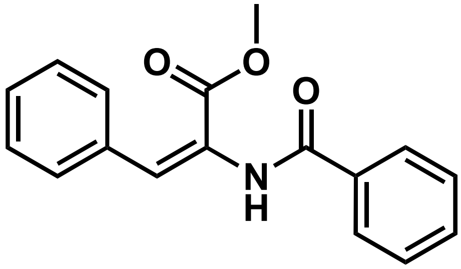

methyl (*E*)-2-benzamido-3-phenylacrylate ((*E*)-**3a**). **^{1}H NMR** (400 MHz, $CDCl_3$) δ 8.34 (s, 1H), 8.15 (s, 1H), 7.92 – 7.82 (m, 2H), 7.60 – 7.51 (m, 1H), 7.48 (dd, *J* = 8.2, 6.6 Hz, 2H), 7.31 (m, 5H), 3.68 (s, 3H). **^{13}C NMR** (126 MHz, CDCl3) δ 165.9, 165.6, 135.5, 134.4, 132.2, 129.0, 128.9, 128.0, 127.8, 127.2, 126.6, 126.0, 52.5. This data agrees with literature reports.[25]

Isomerization Study

Procedure 8: To a flame dried 2-dram vial: a stir bar and (*Z*)-**3a** (30.0 mg, 0.107 mmol, 1.0 equiv) were added. The vial was sealed with a septa lid and put under an inert atmosphere using standard *Schlenk* technique. Then, toluene (427 μL, 0.25 M) and tri-*n*-butylphosine (2.7 μL, 0.011 mmol,10 mol%) were added. The vial was added to a preheated oil bath at 40 °C. The reaction was allowed to stir for 16 hours. The reaction was then opened, and volatiles were removed under vacuum. Crude NMR was analysis was then conducted.

Procedure 9: To a flame dried 2-dram vial: a stir bar and (*E*)-**3a** (16.0 mg, 0.057 mmol, 1.0 equiv) were added. The vial was sealed with a septa lid and put under an inert atmosphere using standard *Schlenk* technique. Then, toluene (288 μL, 0.25 M) and tri-*n*-butylphosine (1.0 μL, 0.006 mmol, 10 mol%) were added. The vial was added to a preheated oil bath at 40 °C. The reaction was allowed to stir for 16 hours. The reaction was then opened, and volatiles were removed under vacuum. Crude NMR was analysis was then conducted.

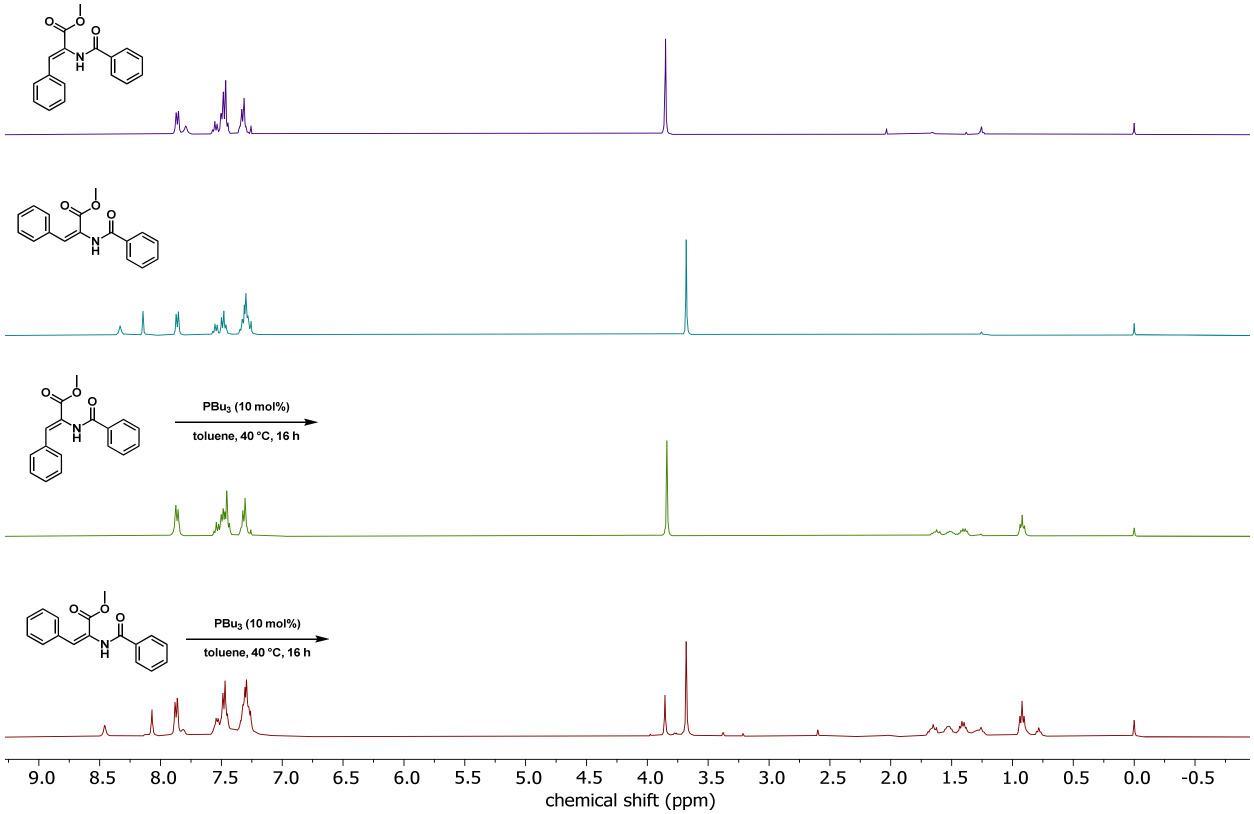

Figure S1: ^{1}H NMR spectra for mechanistic study ($CDCl_3$, 400 MHz).

Procedure 10: To consider whether the presence of benzamide affected the isomerization of (*E*)-**3a**, the sample containing a mixture of (*E*)- and (*Z*)-isomers was dried down and benzamide was added (5.22 mg, 0.043 mmol, 1.2 equiv) and the reaction was put under inert atmosphere using standard *Schlenk* technique. Then, toluene (288 μL, 0.25 M) and tri-*n*-butylphosine (1.0 μL, 0.006 mmol, 10 mol%) were added. The vial was added to a preheated oil bath at 40 °C. The vial was added to a preheated oil bath at 40 °C. After 3.5 hours, an aliquot of the reaction was removed via syringe and volatiles were removed under vacuum. Crude NMR was analysis was then conducted.

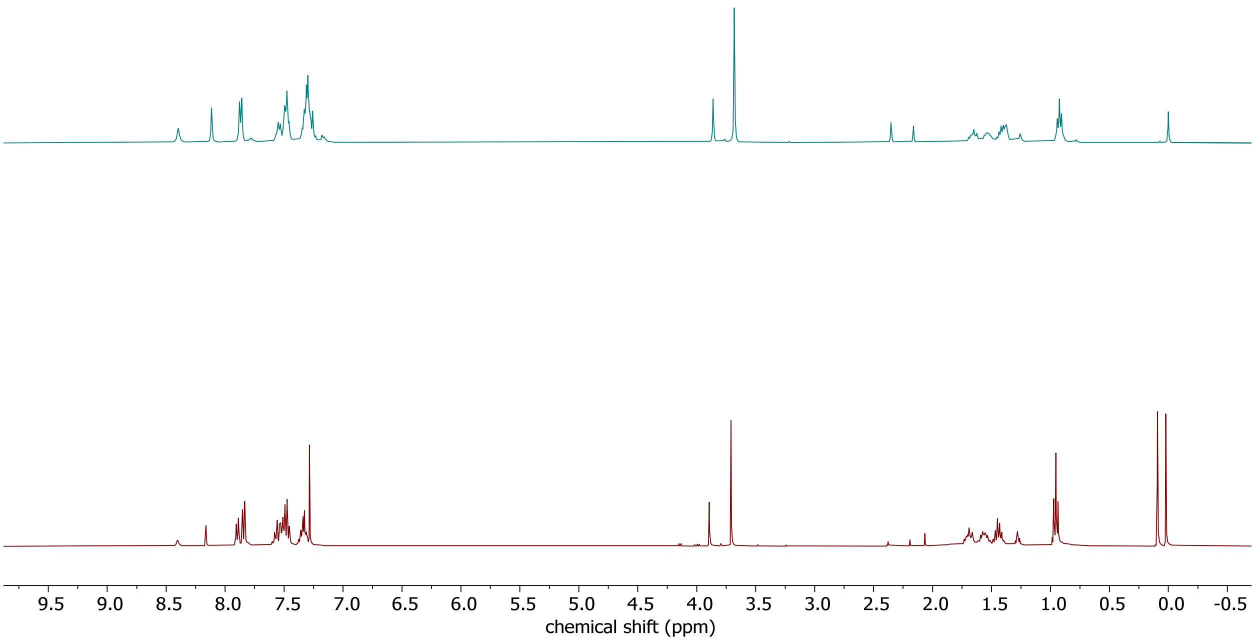

Figure S2: ^{1}H NMR spectra for mechanistic study including benzamide ($CDCl_3$, 400 MHz).

7. Epimerization Study

The goal of this study was to determine whether the amino amides were epimerized during the phosphine-catalyzed umpolung addition to alkynoates. Substrate **3u** was deprotected on the N-terminus (Boc-deprotection) and reacted with (*R*)-Mosher's acid chloride. If the amino amide was epimerized, diastereomers would be evident in the crude NMR.

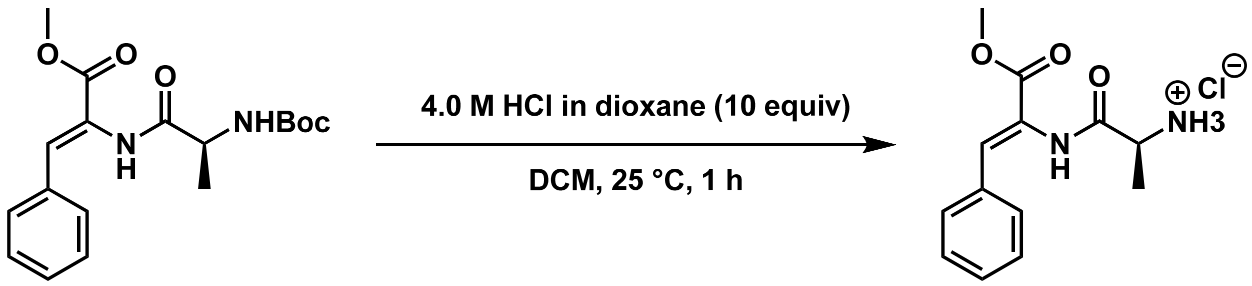

3u

S5

Scheme S8: Boc-deprotection of substrate **3u**.

Procedure 11: To a 6-dram vial: a stir bar and **3u** (75.0 mg, 0.215 mmol, 1.0 equiv) were added. Dry DCM (861 µL, 0.25 M) and 4.0 M HCl in dioxane (538 µL, 2.15 mmol, 10 equiv) were added. The reaction was allowed to stir for 1 hour. Upon completion of the reaction as indicated by TLC analysis, volatiles were removed under vacuum. Then **S5** was triturated with hexanes and dried under vacuum to afford **S5** as a colorless solid (50.0 mg, 0.215 mmol, quant. yield). **^{1}H NMR** (400 MHz, DMSO-d$_6$) δ 10.18 (s, 1H), 8.28 (s, 3H), 7.70 – 7.64 (m, 2H), 7.47 – 7.40 (m, 3H), 7.39 (s, 1H), 4.05 (m, 1H), 3.73 (s, 3H), 1.47 (d, *J* = 7.0 Hz, 3H). **^{13}C NMR** (126 MHz, DMSO-d$_6$) δ 169.6, 164.9, 133.3, 132.9, 130.1, 129.8, 128.7, 125.0, 52.4, 48.3, 16.7. **HRMS** (ESI) m/z [M-Cl]$^+$ calcd for $C_{13}H_{17}N_2O_3^+$ 249.1234; found 249.1243.

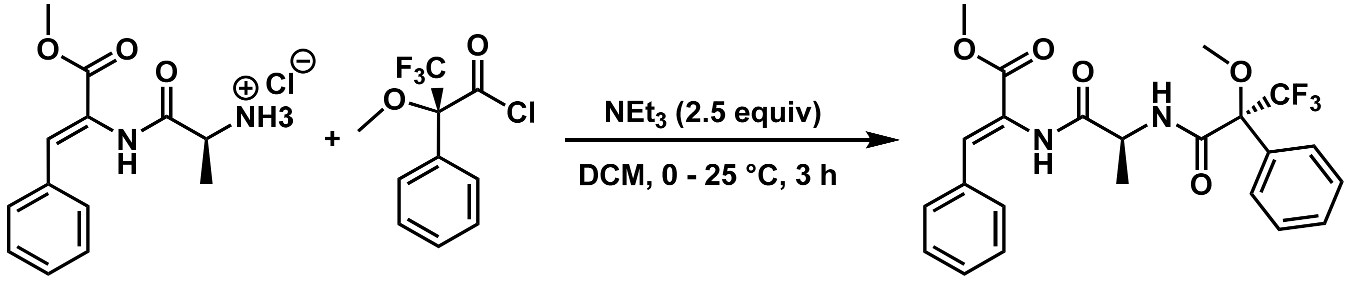

S5 **1.2 equiv** **S6**

Scheme S9: Synthesis of Mosher's amide from **S5**.

Procedure 12: To a flame dried 2-dram vial: a stir bar, (*R*)-3,3,3-trifluoro-2-methoxy-2-phenylpropanoyl chloride (45.7 mg, 0.181 mmol, 1.2 equiv) and **S5** (35.0 mg, 0.151 mmol, 1.0 equiv) were added and the vial was added to an ice-bath. Dry DCM (603 µL, 0.25 M) and triethylamine (53 µL, 0.377 mmol, 2.5 equiv) were then added. The reaction was allowed to stir for 3 hours. The reaction was aliquoted and dried under vacuum for crude NMR. After crude analysis, celite was added and volatiles were removed under vacuum. Then **S6** was isolated using flash chromatography using a 0-60% EtOAc:Hex gradient. (47.9 mg, 0.103 mmol, 68% yield). **^{1}H NMR** (400 MHz, $CDCl_3$) δ 8.00 (s, 1H), 7.54 – 7.42 (m, 6H), 7.42 – 7.27 (m, 6H), 4.79 (dt, *J* = 7.2, 6.9 Hz, 1H), 3.79 (s, 3H), 3.39 (s, 3H), 1.42 (d, *J* = 7.0 Hz, 3H). **^{13}C NMR** (151 MHz, $CDCl_3$) δ 170.4, 166.7, 165.4, 134.0, 133.5, 132.6, 129.8, 129.7, 129.7, 128.7, 128.6, 127.4, 123.7 (q, *J* = 290.2 Hz), 123.6, 83.9 (q, *J* = 26.2 Hz), 55.2, 52.7, 49.1, 18.0. **^{19}F NMR** (376 MHz, $CDCl_3$) δ -68.6 (s, 3F). **HRMS** (ESI) m/z $[M+H]^+$ calcd for $C_{23}H_{24}F_3N_2O_5^+$ 465.1632; found 465.1639.

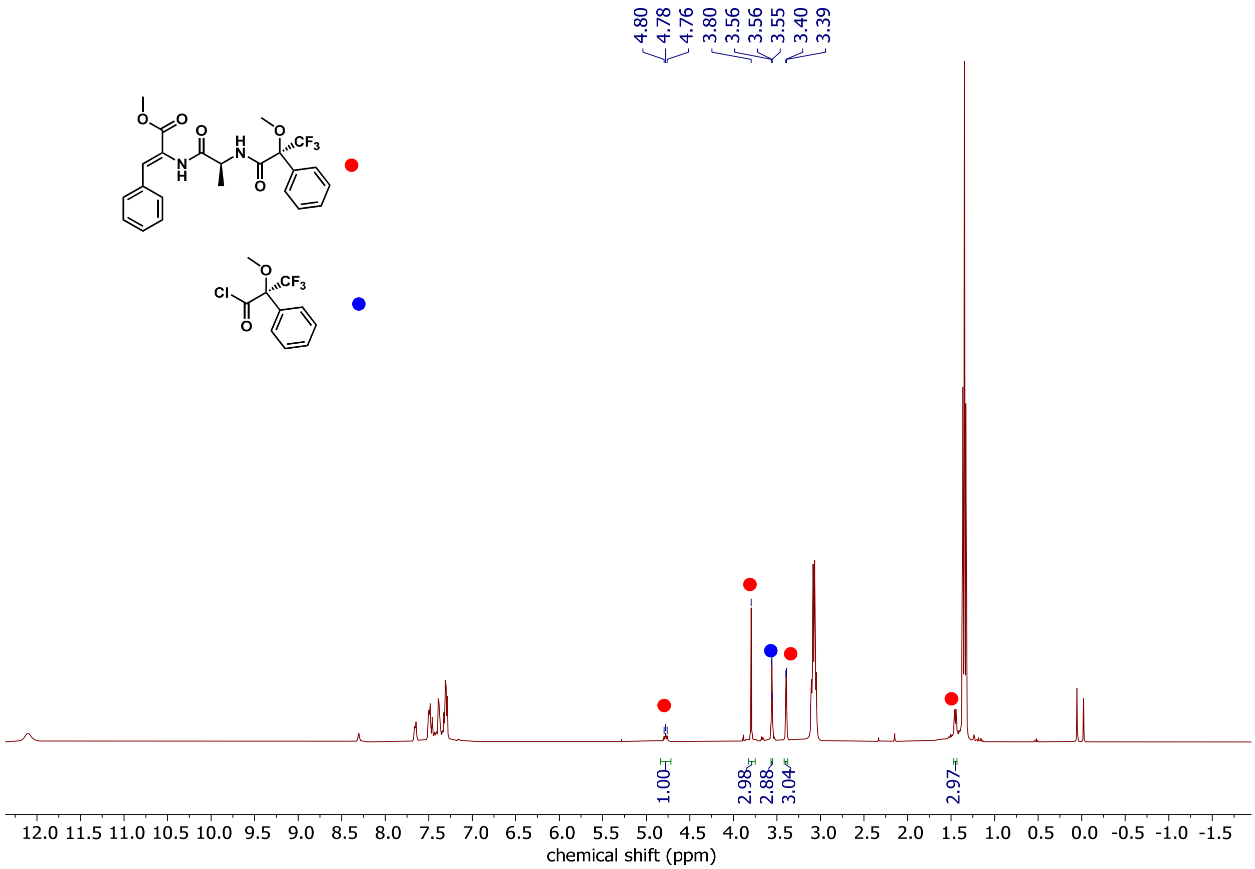

Figure S3: Crude ^{1}H NMR spectra for Mosher's amide synthesis ($CDCl_3$, 400 MHz).

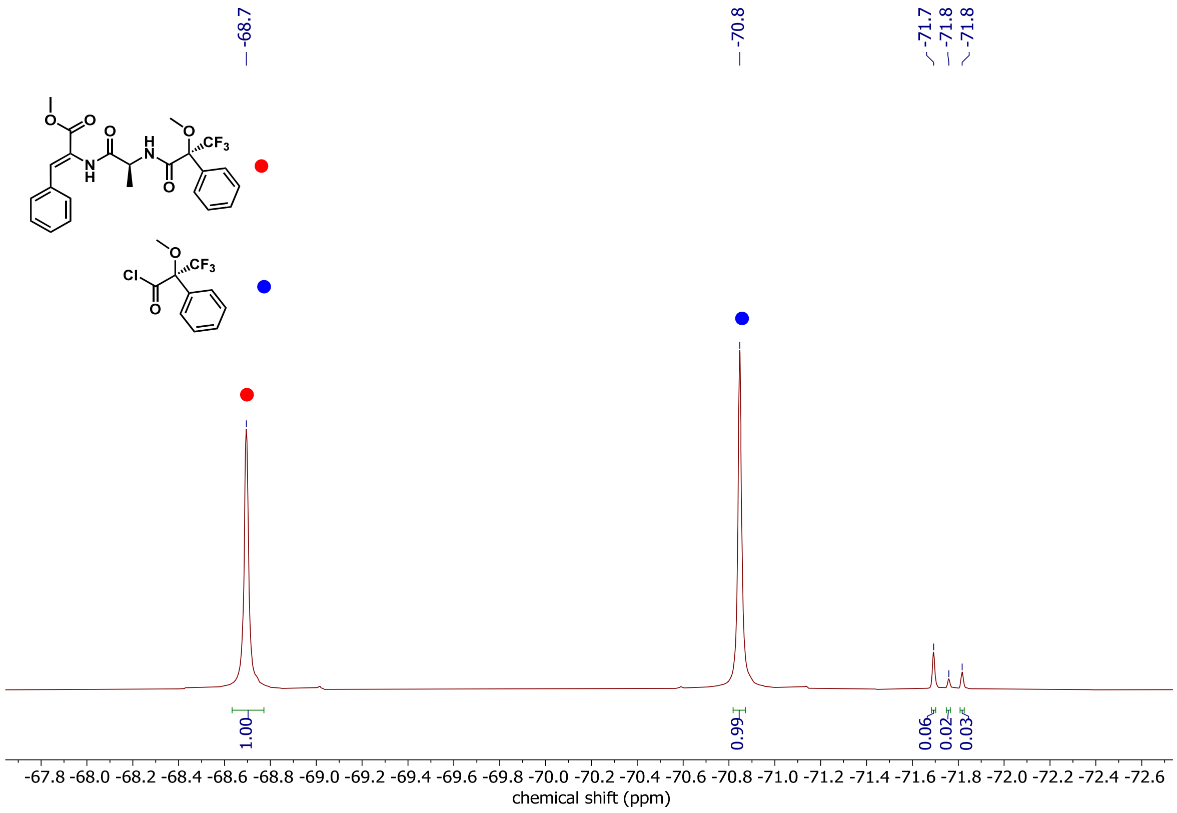

Figure S4: Crude ^{19}F NMR spectra for Mosher's amide synthesis ($CDCl_3$, 376 MHz).

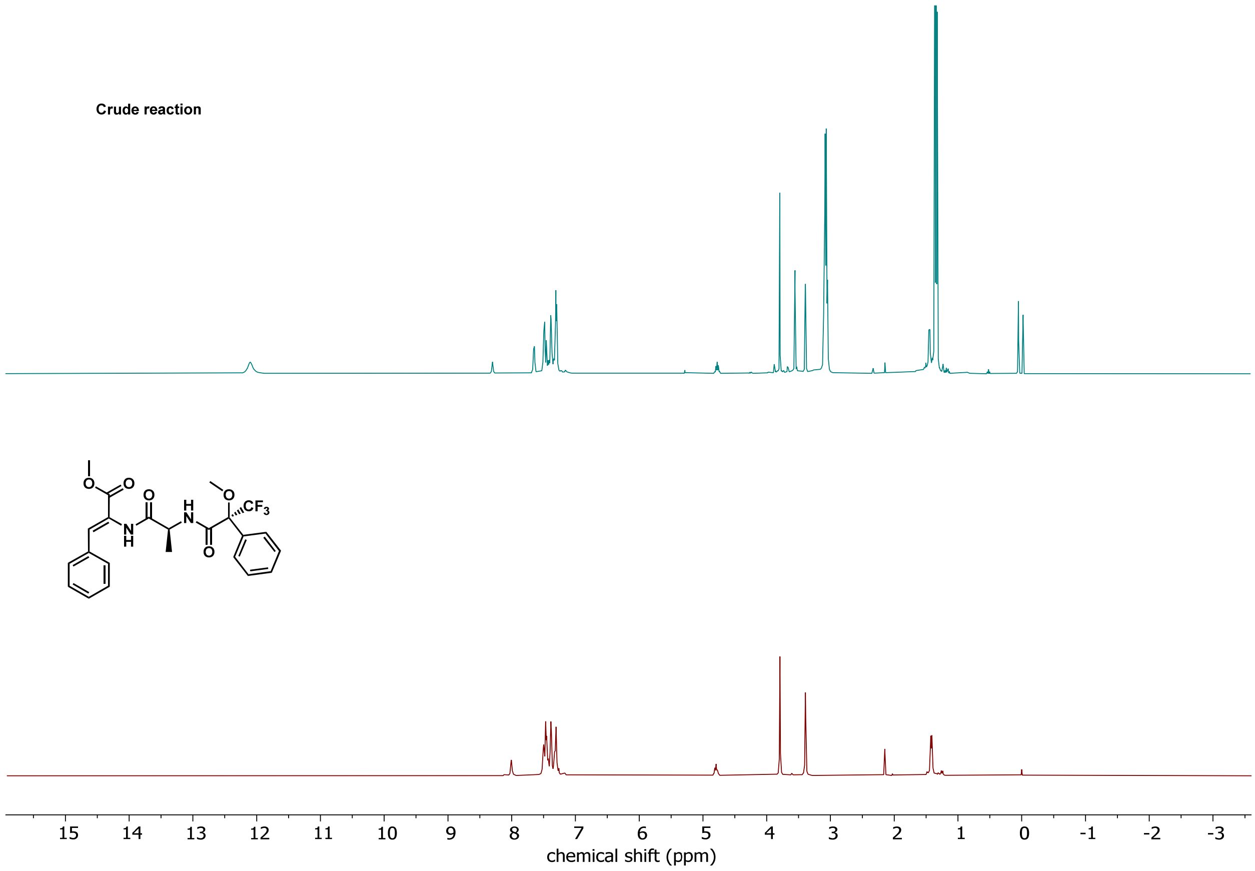

Figure S5: Crude ^{1}H NMR spectra for Mosher's amide synthesis stacked against authentic sample **S6**. ($CDCl_3$, 400 MHz).

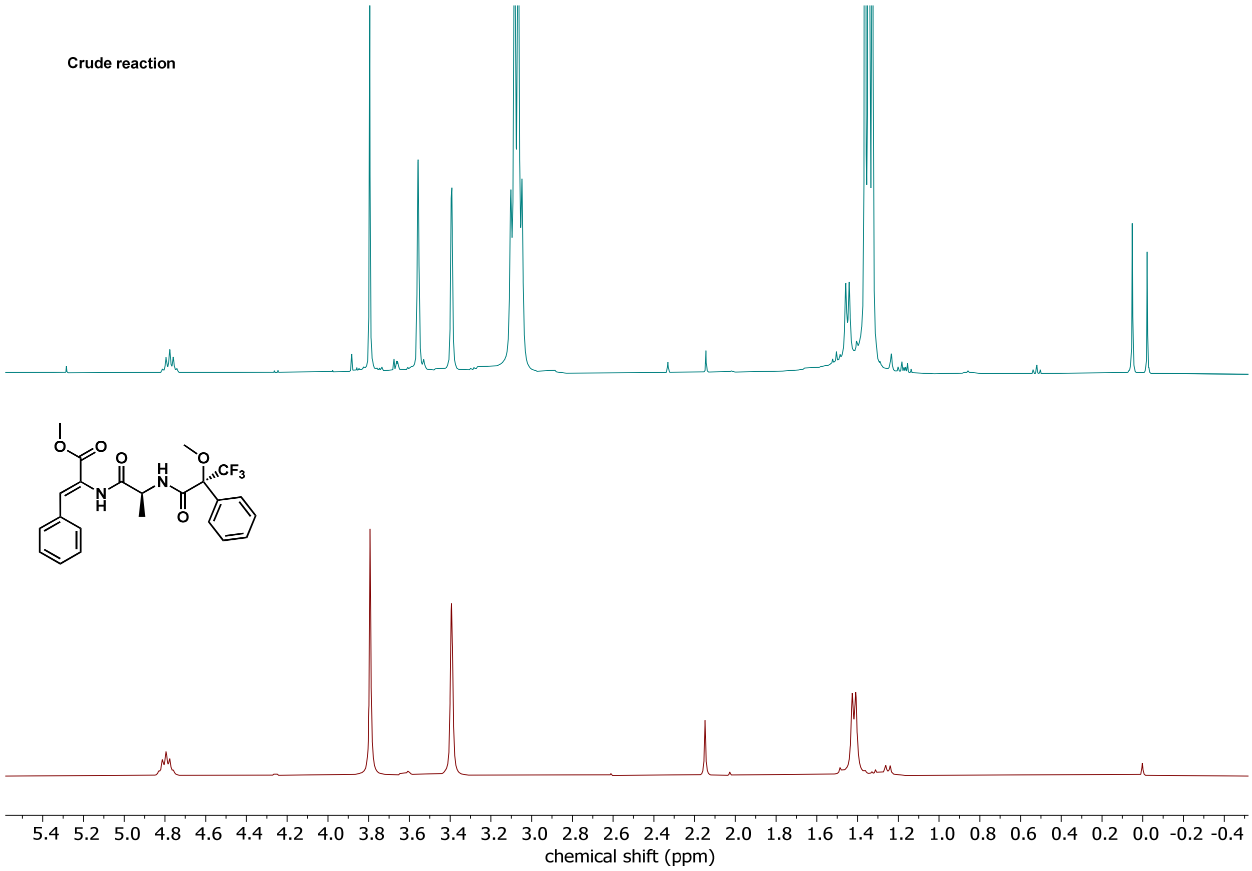

Figure S6: Zoomed in crude ^{1}H NMR spectra for Mosher's amide synthesis stacked against authentic sample **S6**. ($CDCl_3$, 400 MHz).

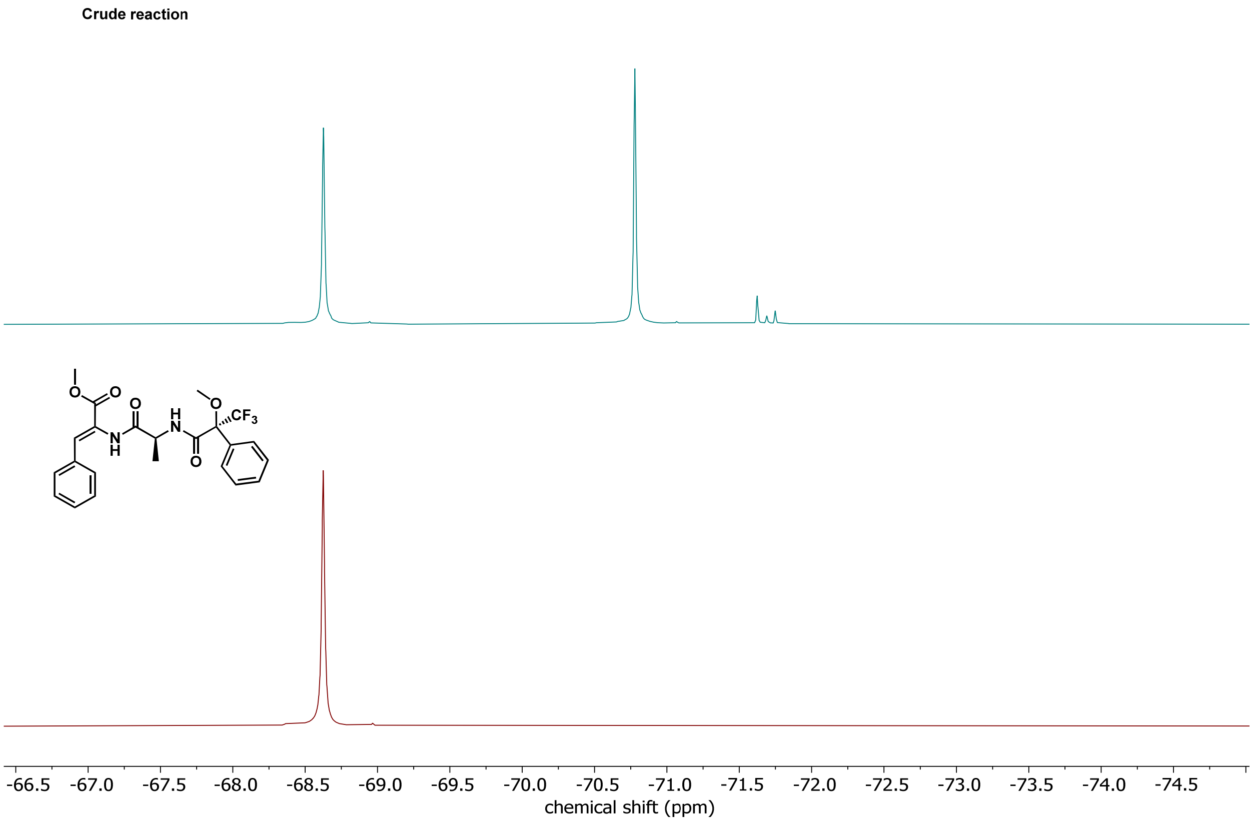

Figure S7: Crude ^{19}F NMR spectra for Mosher's amide synthesis stacked against authentic sample **S6**. ($CDCl_3$, 376 MHz).

8. Crystallographic Data

X-Ray Diffraction Techniques. All structures were collected on a Rigaku Oxford Diffraction Synergy-S diffractometer equipped with a HyPix6000HE detector and operating with CuKα radiation source. Data collection, unit cell refinement, and data processing were carried out with CrysAlisPro,[26] while structures were solved utilizing SHELXS[27] and refined using SHELXL[28] via Olex2.[29] Olex2, PovRay,[30] and ORTEP[31] applications were used to generate structure graphics. Crystals were mounted on a cryoloop or glass fiber pin using Paratone N oil. Structures were collected at 100 K. All non-hydrogen atoms were refined anisotropically. Hydrogen atoms were placed at idealized positions and refined using a riding model. The isotropic displacement parameters of all hydrogen atoms were fixed to 1.2 times the atoms they are linked to (1.5 times for methyl groups). Further details on particular structures are noted below.

[$C_{19}H_{17}N_3O$] (**3b**) Crystals were grown via slow diffusion in EtOAc at room temperature. The structure was solved in the orthorhombic space group $Pna2_1$ with 4 molecules per unit cell. The final refinement model involved anisotropic displacement parameters for non-hydrogen atoms. A riding model was used for the C-H hydrogen atoms. The N-H and O-H hydrogen atoms were located from the difference electron density map, and the positions and isotropic displacement parameters were refined independently.

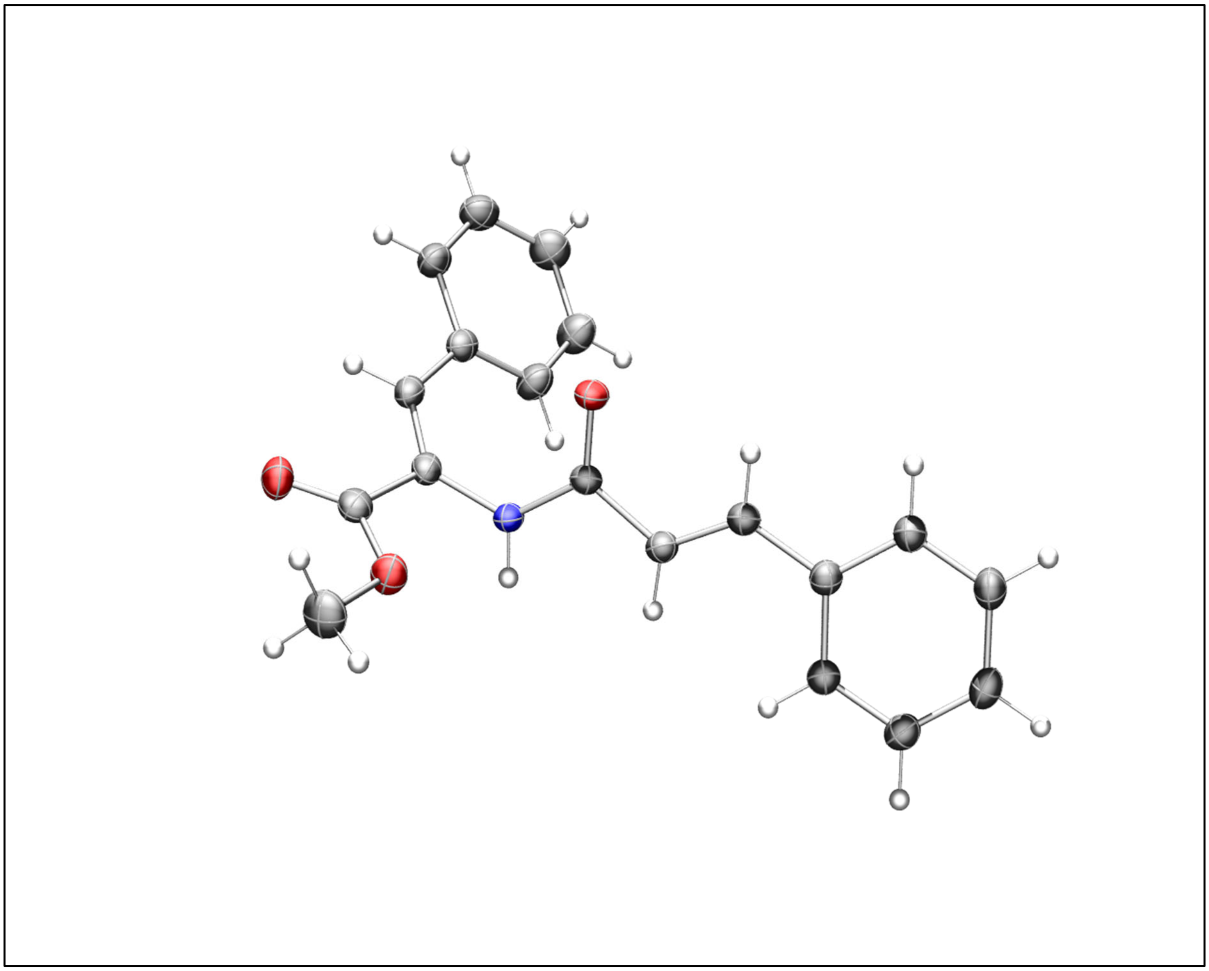

Figure S8: Solid-state molecular structure of compound **3b** with anisotropic displacement ellipsoids at 50% probability level ;Color scheme: O, red; N,blue; C,gray; H, white.

Table S3. X-ray diffraction experimental details

	$[C_{19}H_{17}N_3O]$ (3b)
Empirical Formula	$C_{19}H_{17}N_3O$
Formula Weight	307.33
Temp. (K)	100
Radiation	Cu Kα (λ = 1.54184)
Crystal System	Orthorhombic
Space Group	Pna21

a (Å)	9.65680(10)
b (Å)	19.8800(2)
c (Å)	8.24520(10)
α (°)	90
β (°)	90
γ (°)	90
Volume (Å^3)	1582.89(3)
Z	4
ρ_{calc} (g/cm^3)	1.290
μ (mm^{-1})	0.709
F(000)	648.0
Crystal size (mm^3)	0.6 × 0.39 × 0.03
2Θ range for data collection (°)	8.896 to 154.894
Index Ranges	-9 ≤ h ≤ 12, -25 ≤ k ≤ 25, -10 ≤ l ≤ 10
Reflections Collected	51679
Independent Reflections	3338
Data/Restraints/Parameters	3338/1/277
GOF	1.068
Final R indexes [I>=2σ (I)]	R1 = 0.0370, wR2 = 0.0976
Final R indexes [all data]	R1 = 0.0373, wR2 = 0.0978
Largest diff. peak/hole/e (Å^{-3})	0.21/-0.20
Flack Parameter	0.2(2)

9. NMR Spectra

^{1}H NMR **3a** (400 MHz) in $CDCl_3$

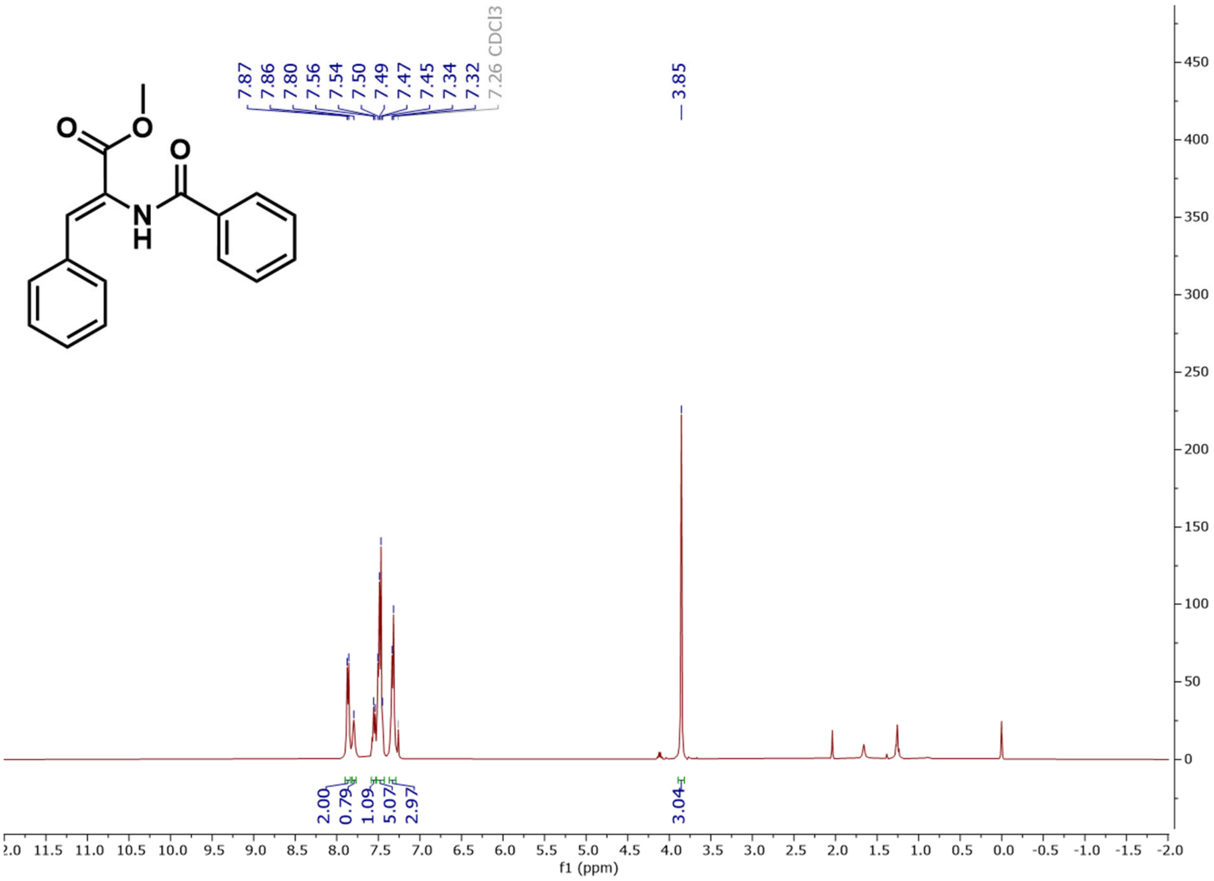

^{13}C NMR **3a** (101 MHz) in $CDCl_3$

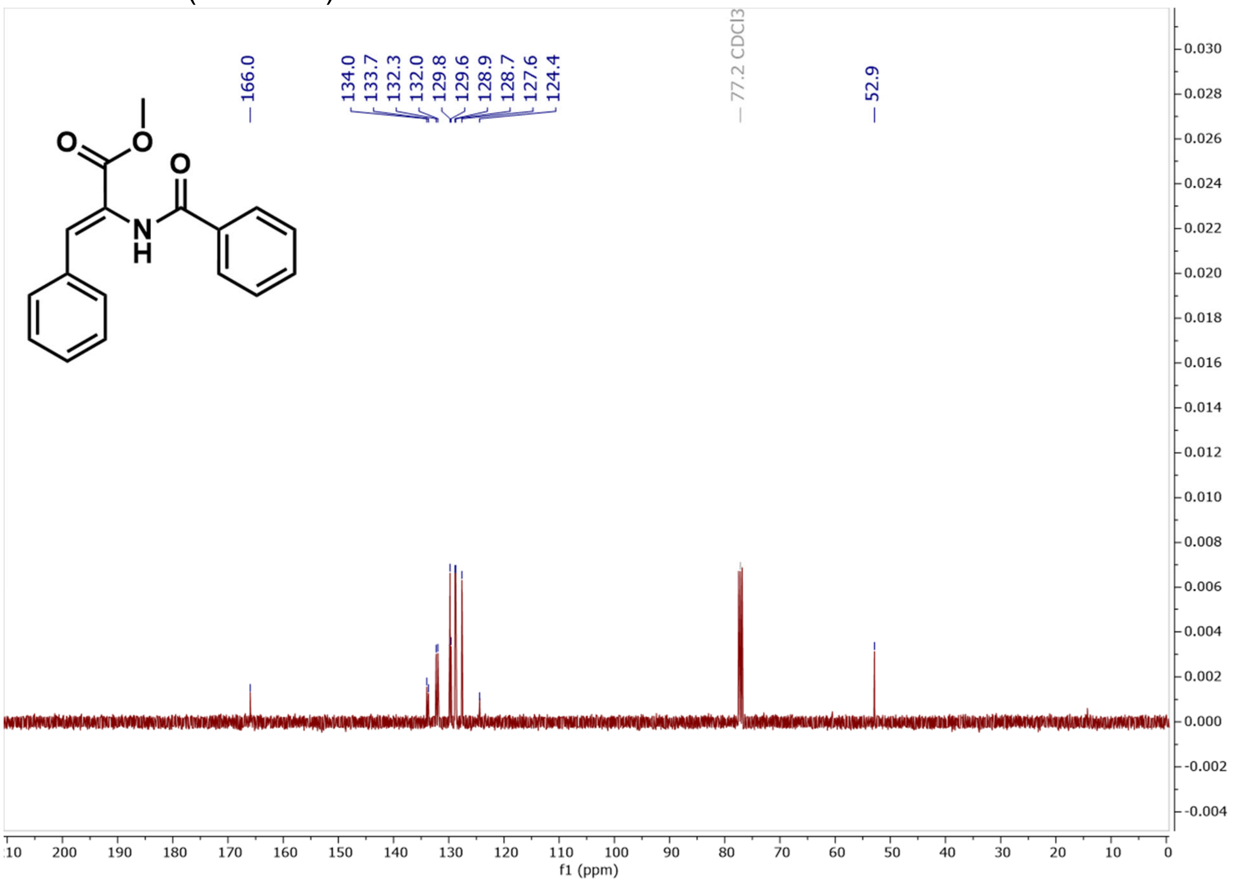

^{1}H NMR of (*E*)-**3a** (400 MHz) in $CDCl_3$

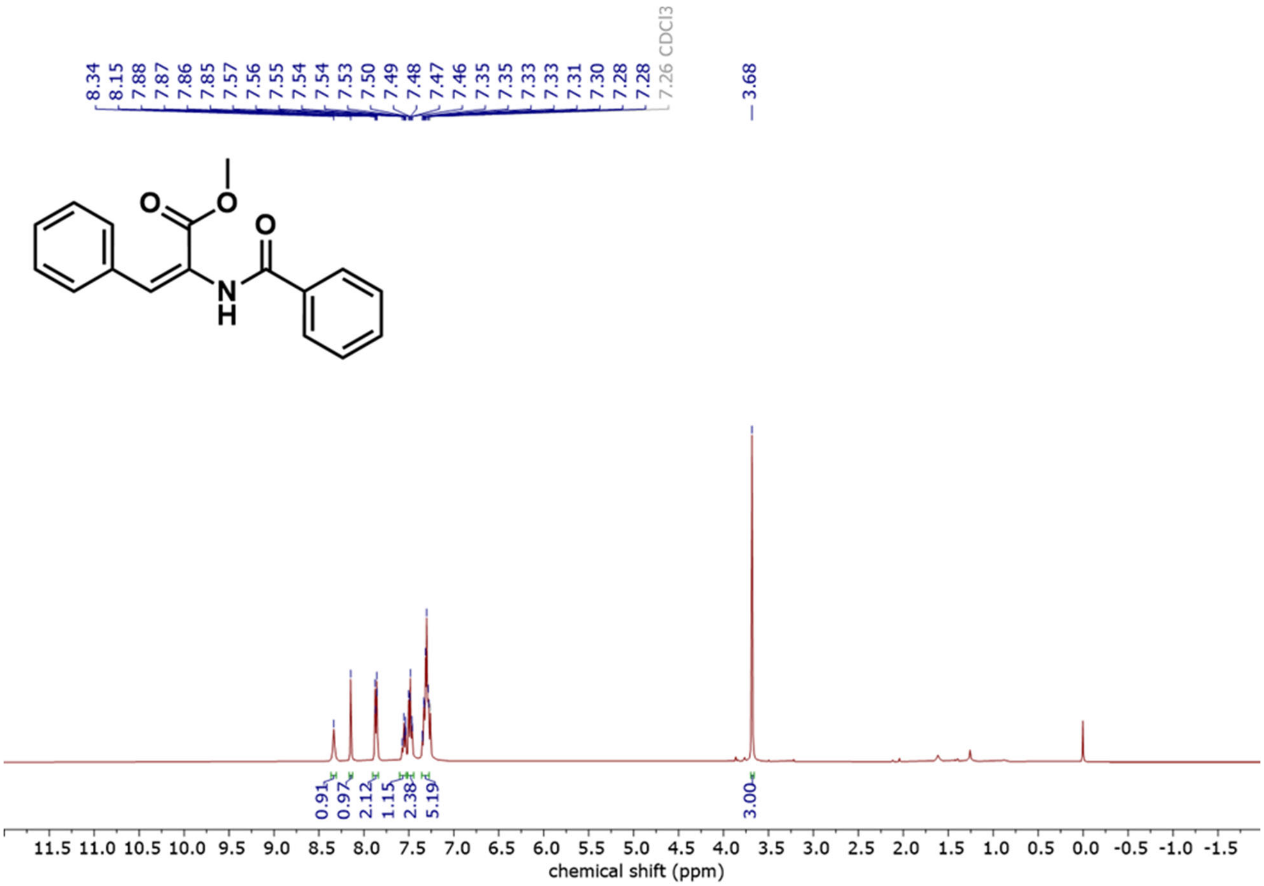

^{13}C NMR of (*E*)-**3a** (126 MHz) in $CDCl_3$

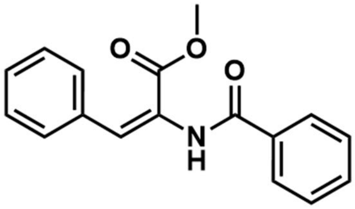

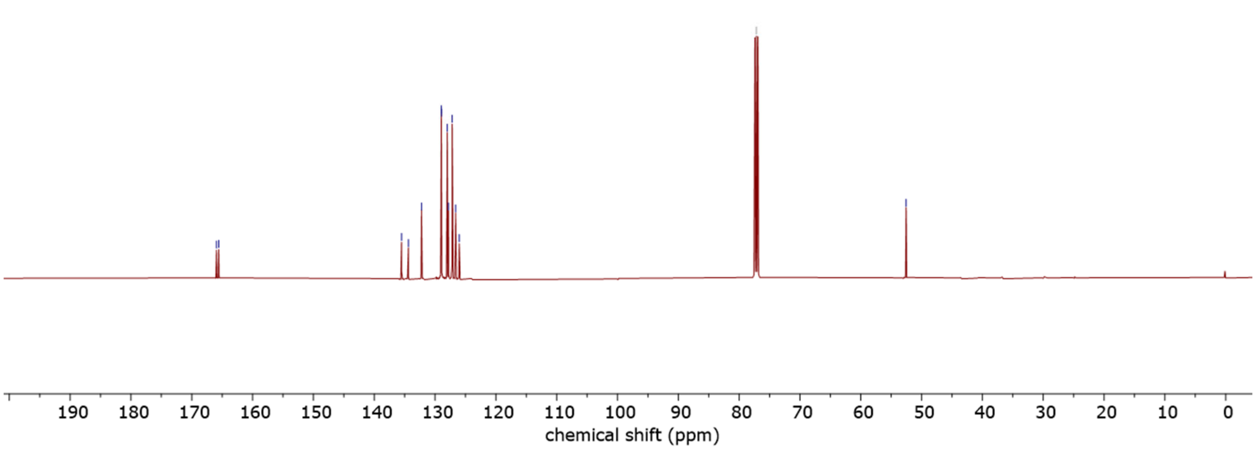

^{1}H NMR **3b** (400 MHz) in $CDCl_3$

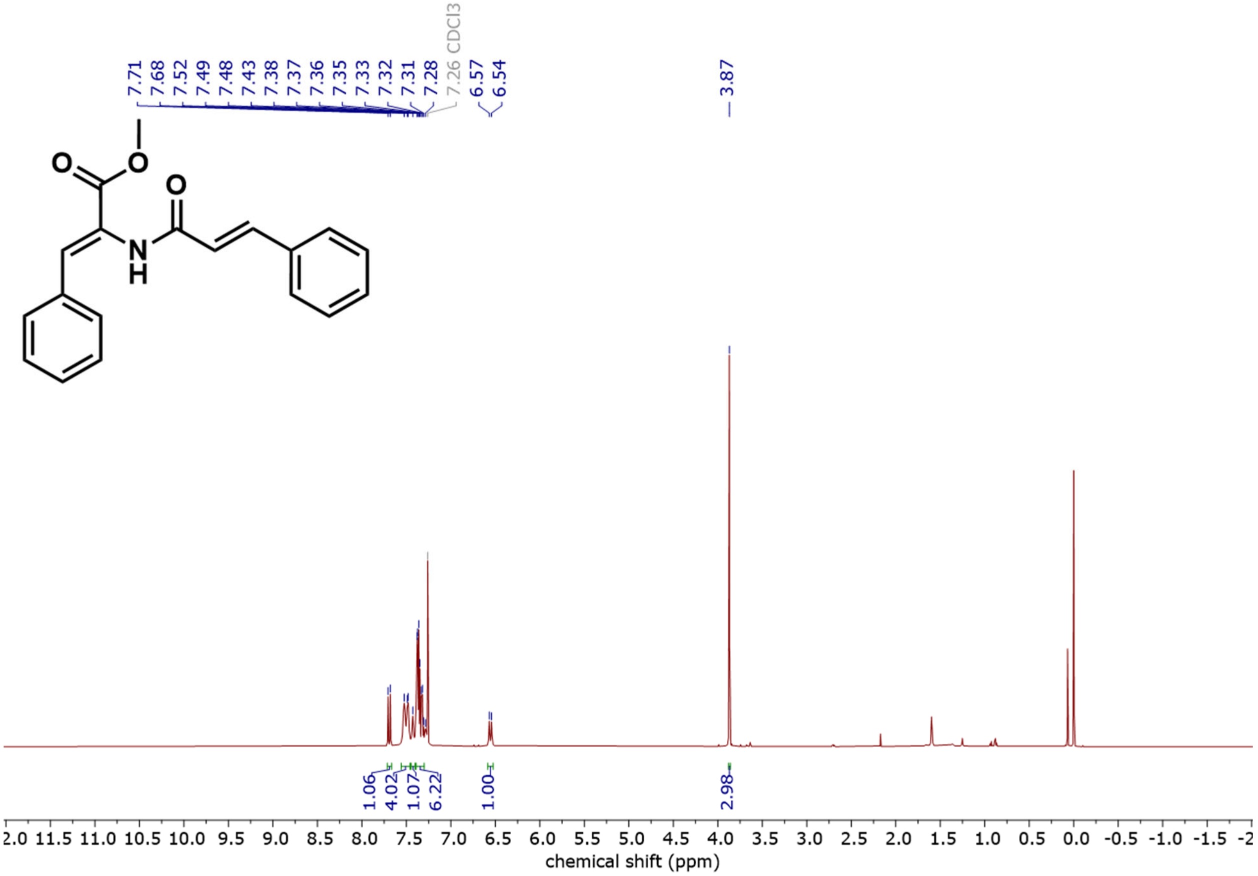

^{13}C NMR **3b** (101 MHz) in $CDCl_3$

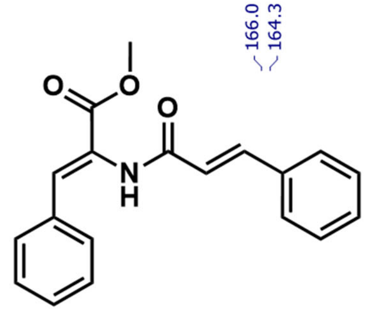

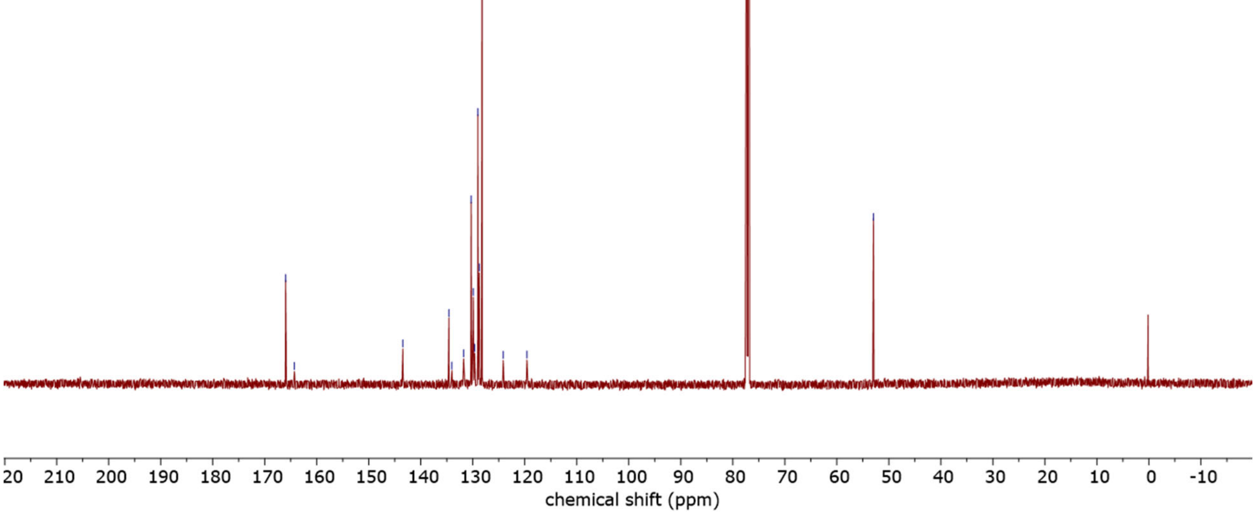

^{1}H NMR **3c** (500 Mz) in $CDCl_3$

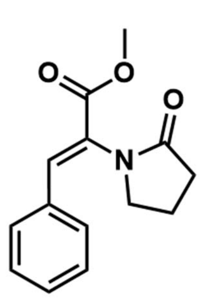

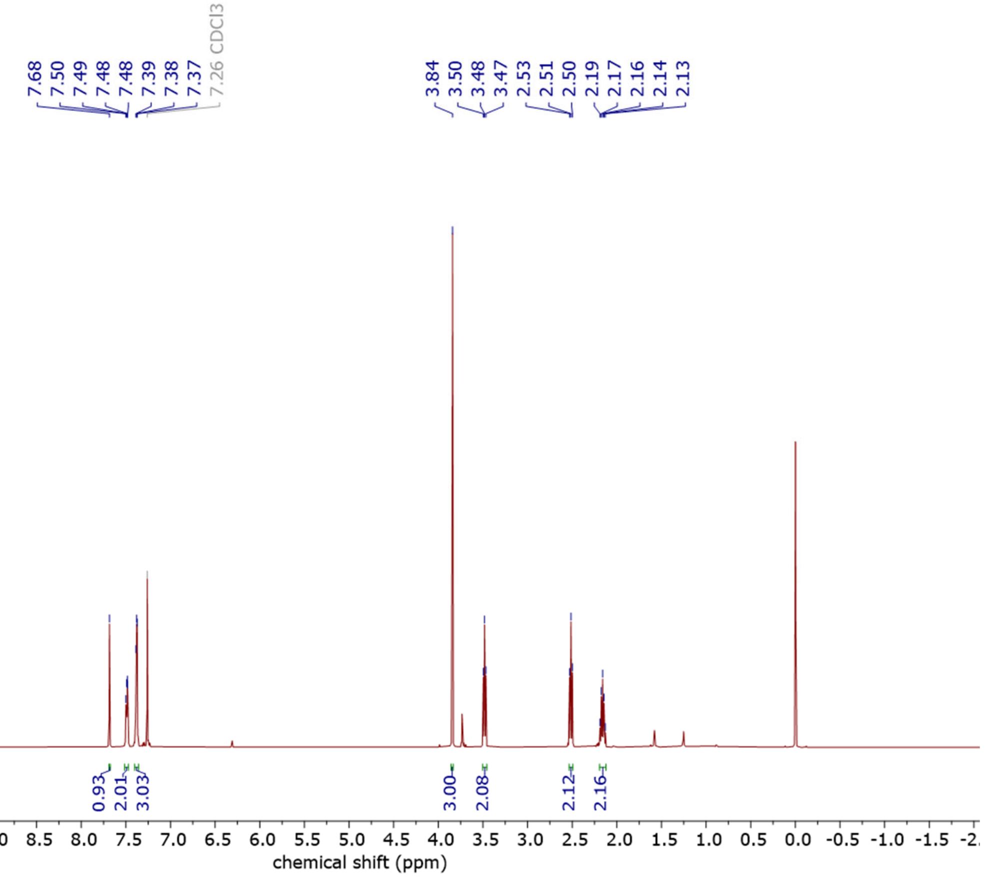

^{13}C NMR **3c** (126 MHz) in $CDCl_3$

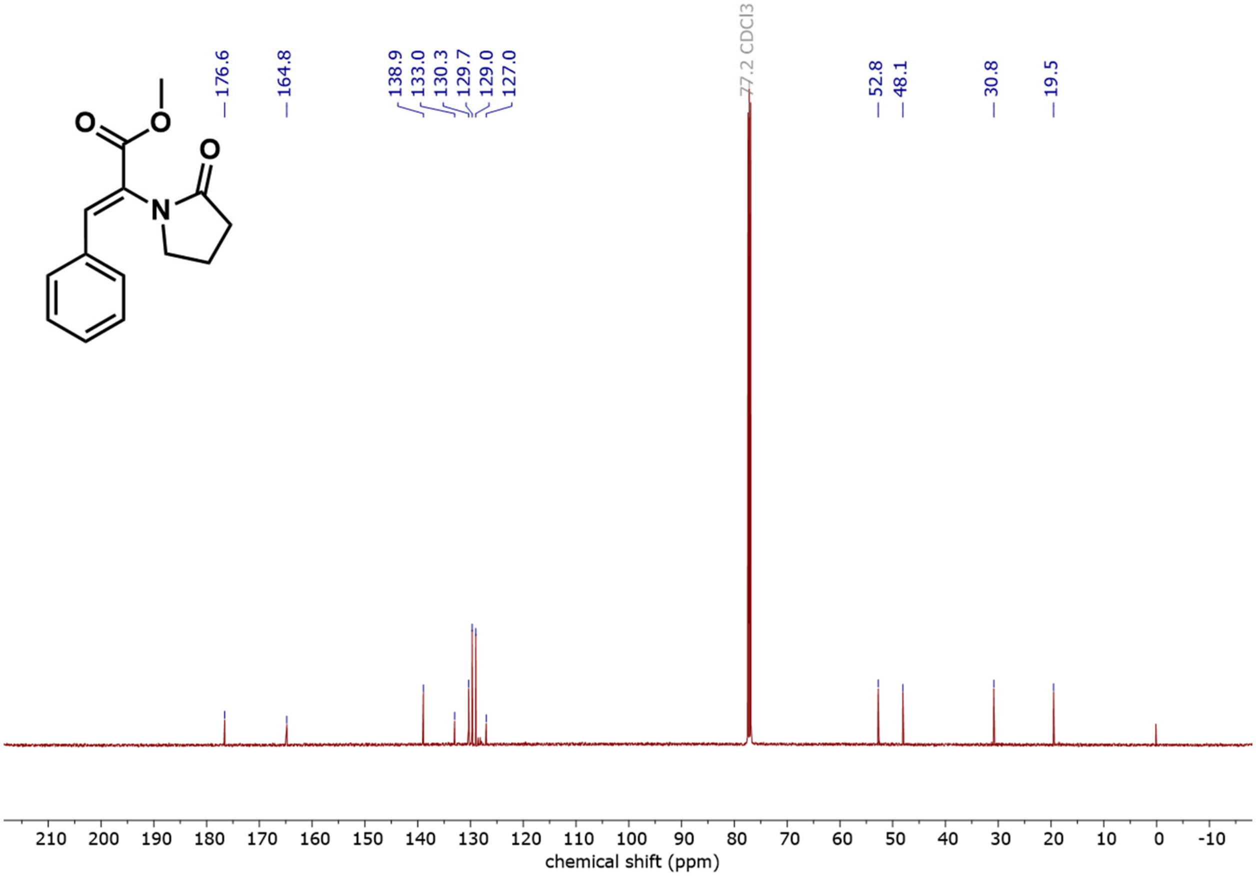

^{1}H NMR **3d** (500 MHz) in $CDCl_3$

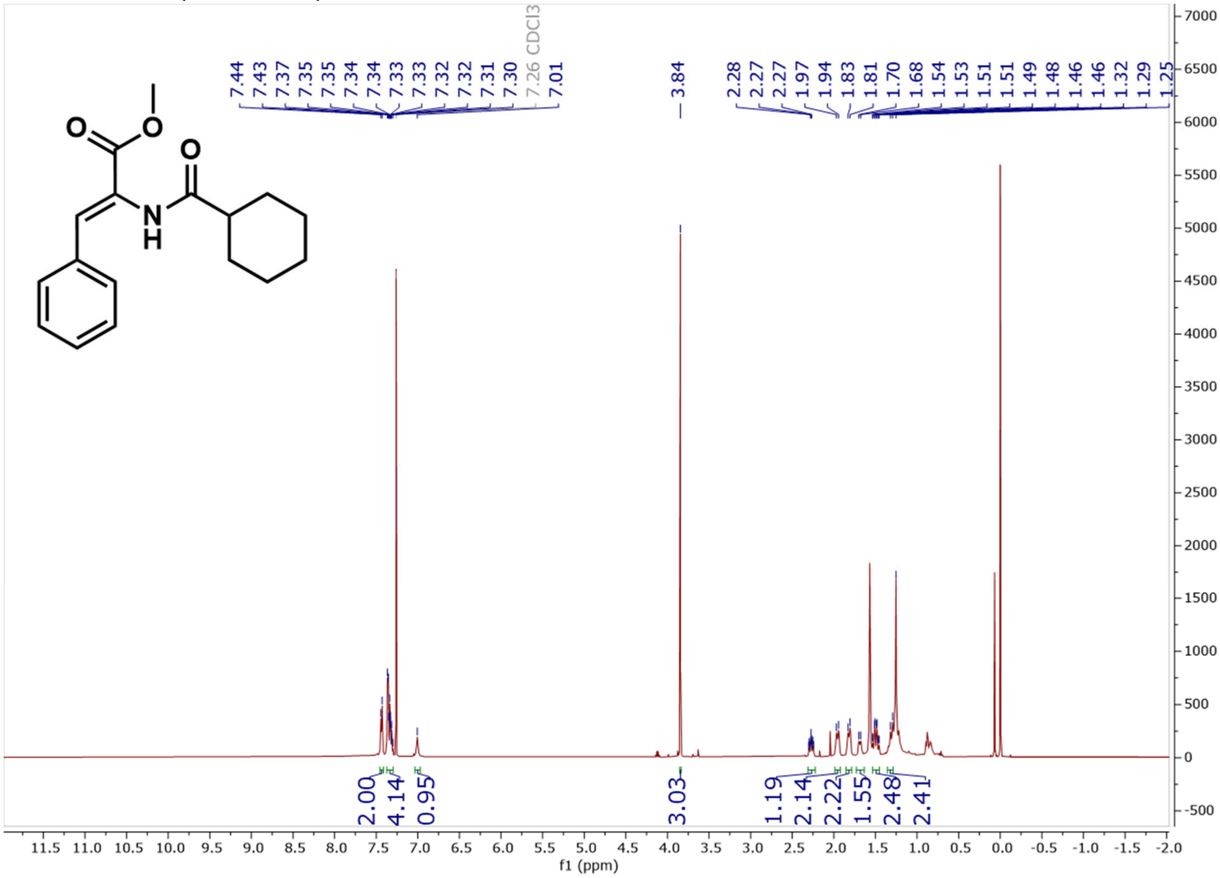

^{13}C NMR **3d** (126 MHz) in $CDCl_3$

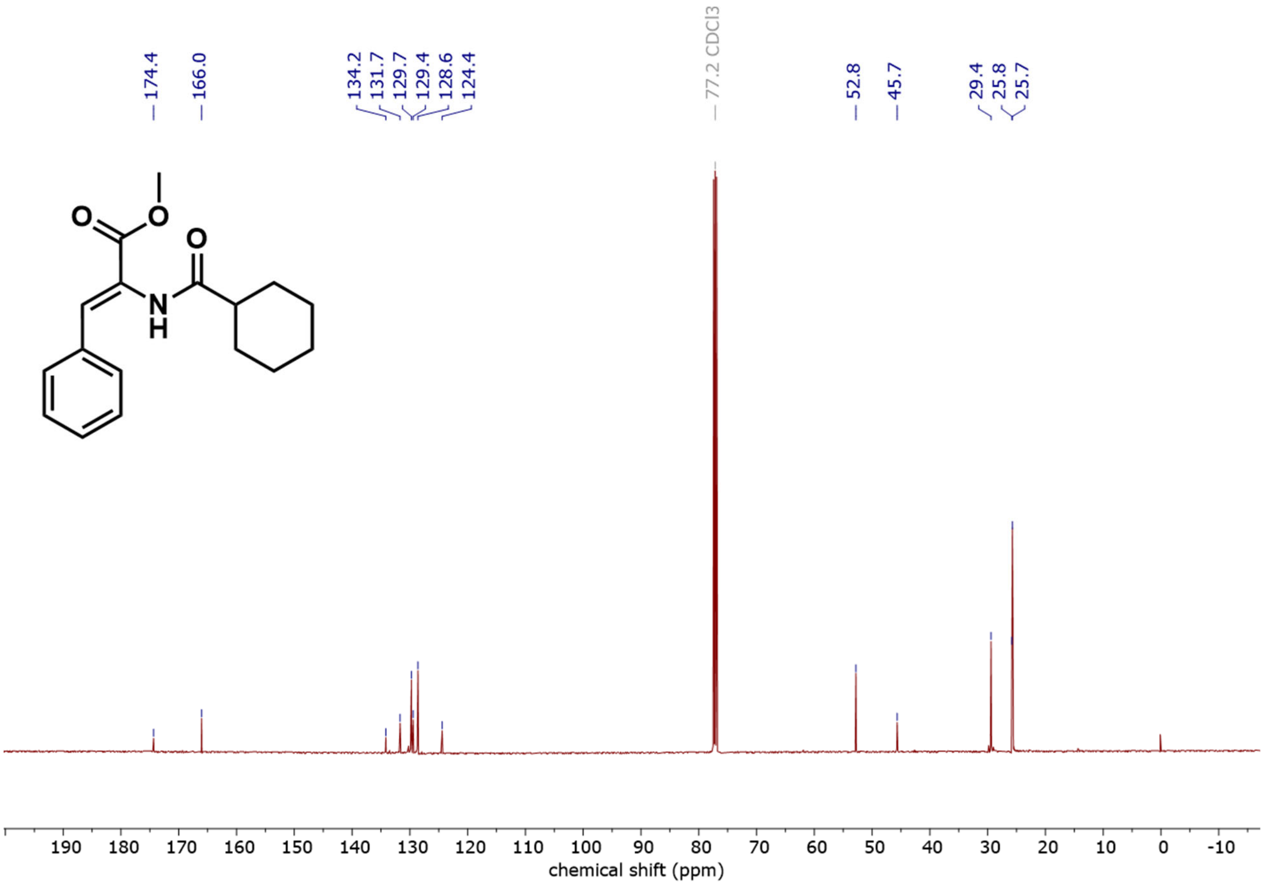

^{1}H NMR **3e** (600 MHz) in $CDCl_3$

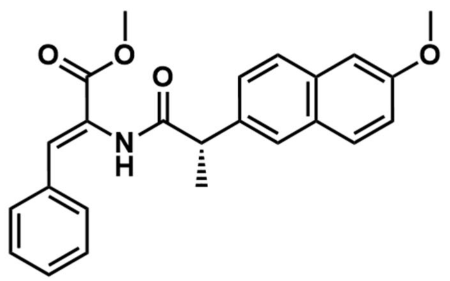

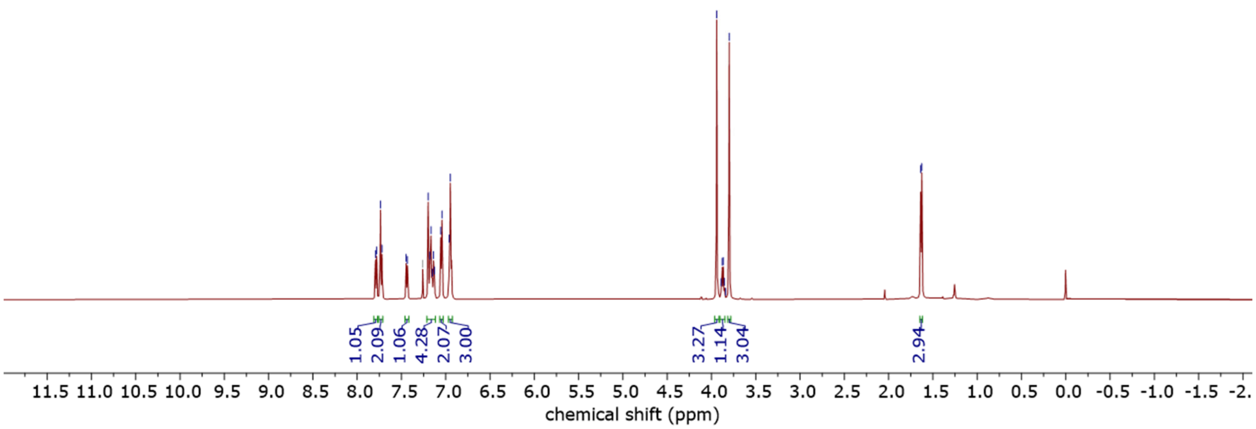

^{13}C NMR **3e** (151 MHz) in $CDCl_3$

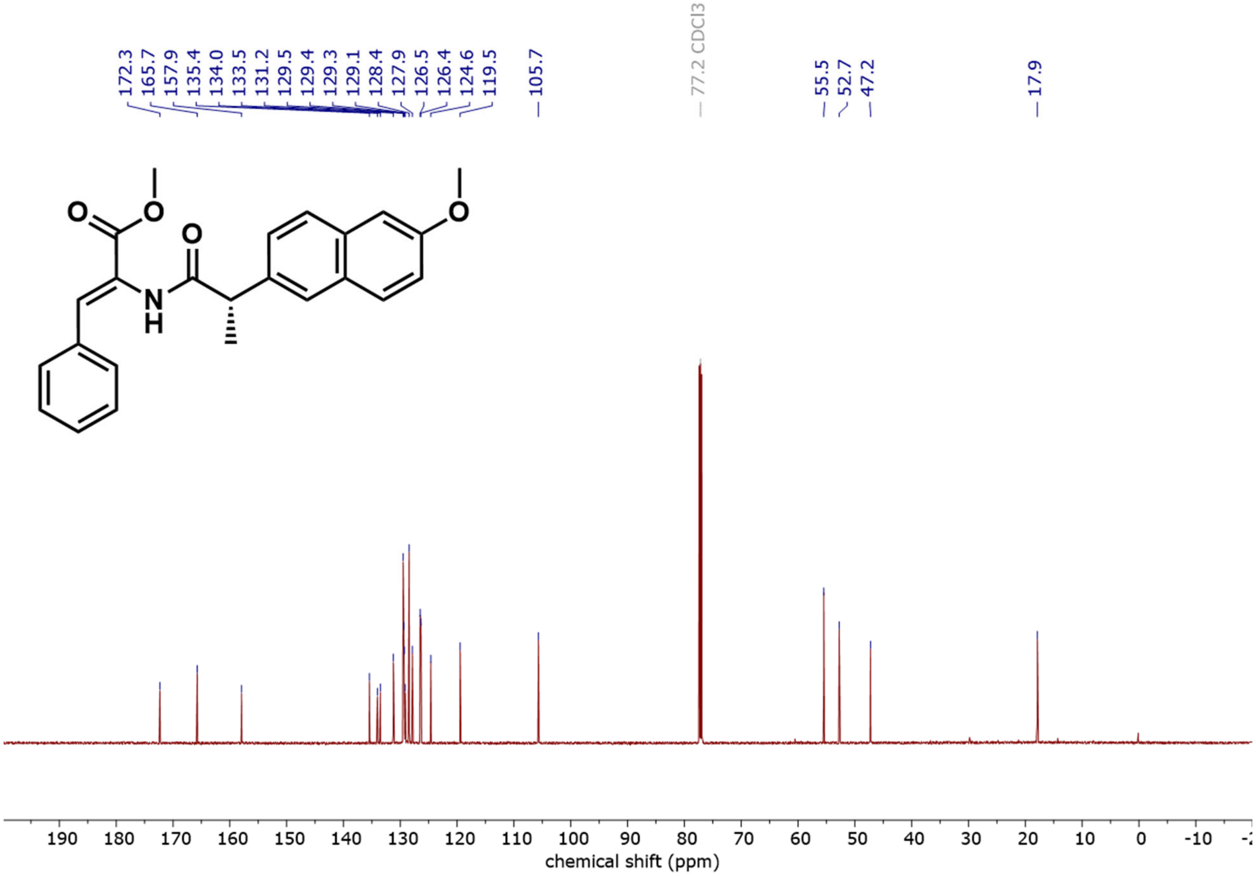

^{1}H NMR **3f** (400 MHz) in $CDCl_3$

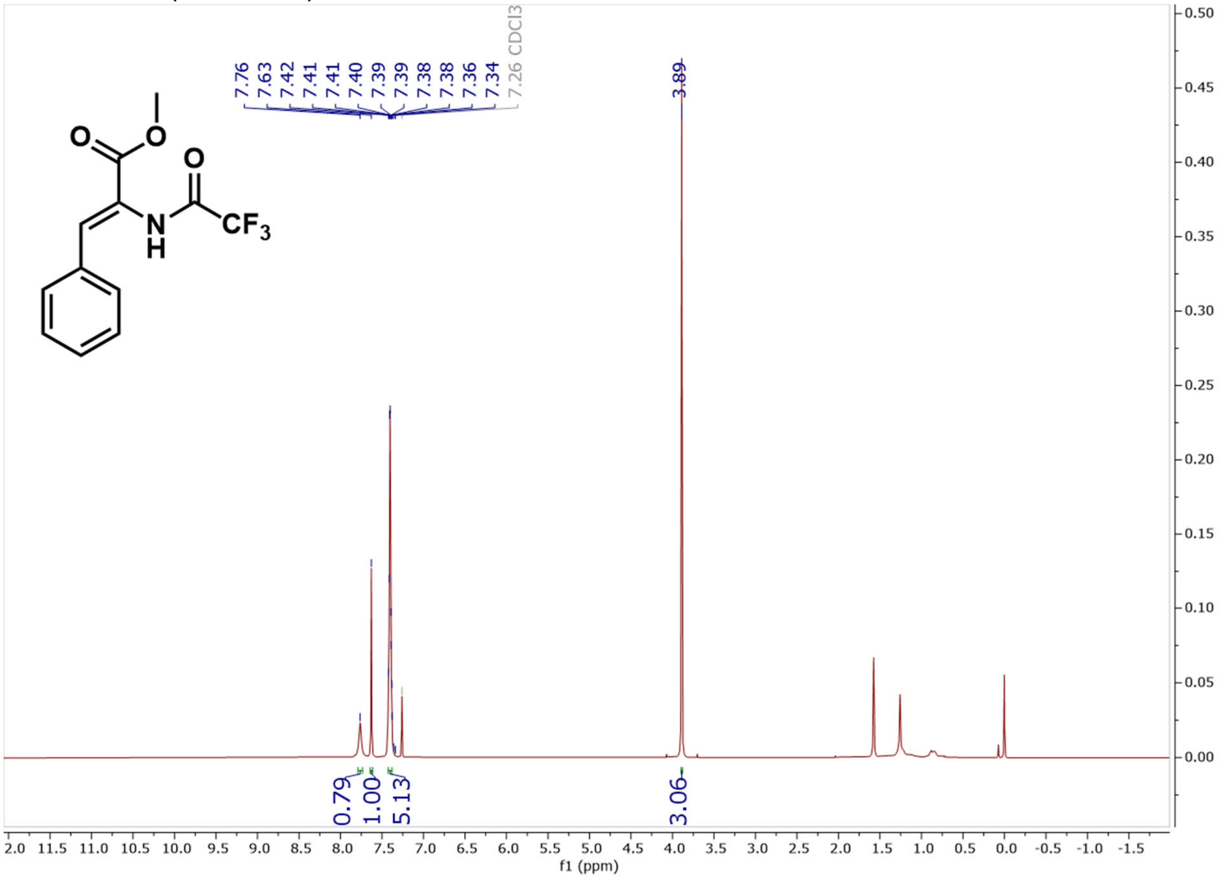

^{13}C NMR **3f** (126 MHz) in $CDCl_3$

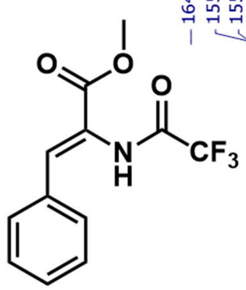

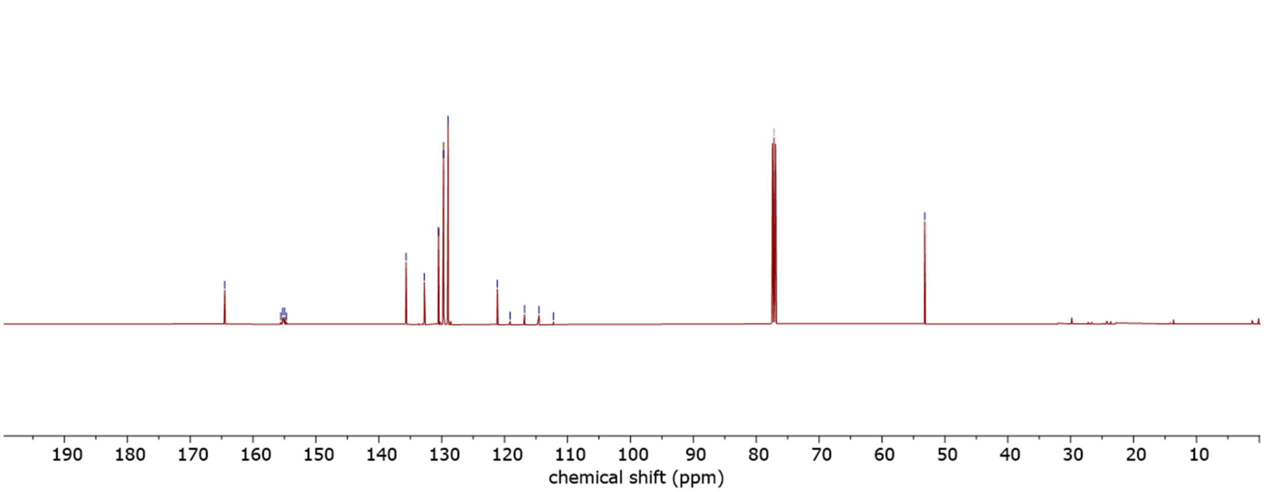

^{19}F NMR **3f** (376 MHz) in $CDCl_3$

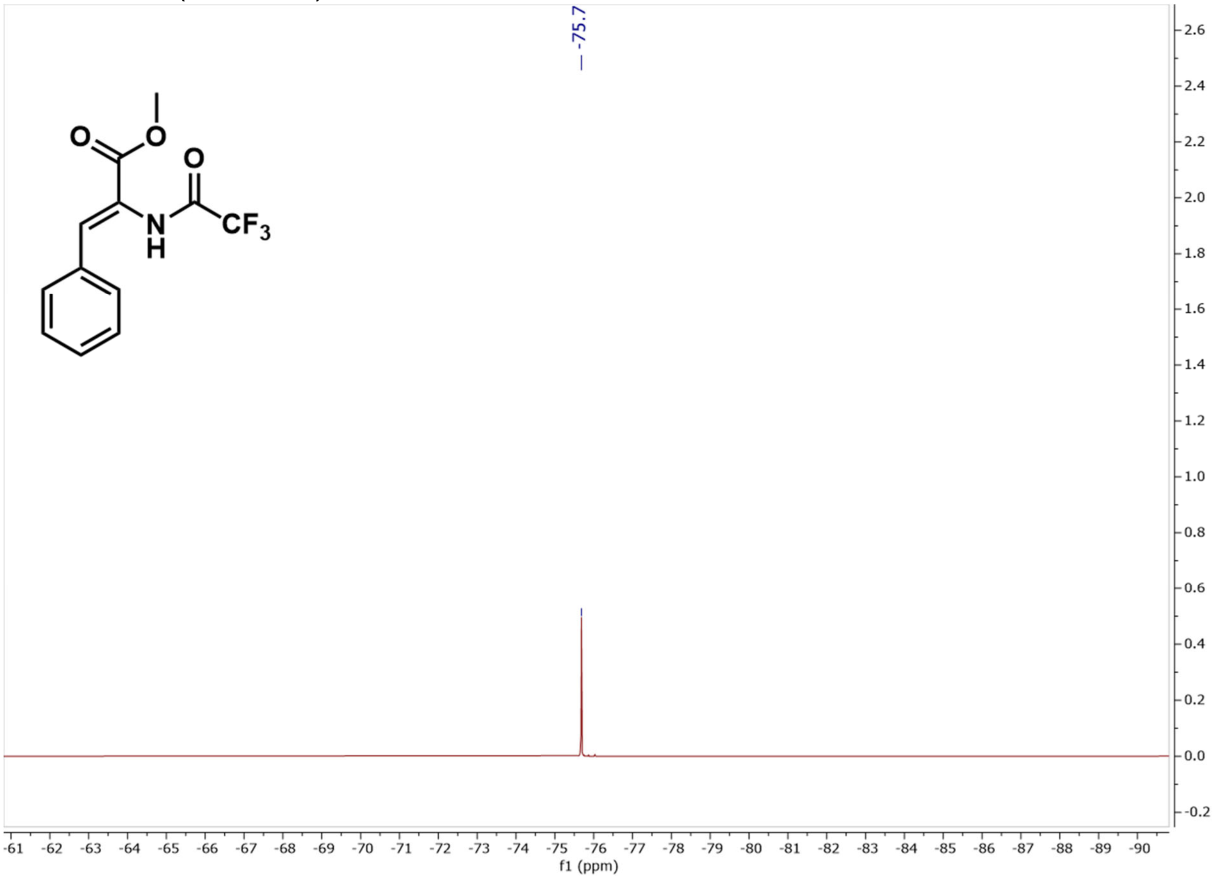

^{1}H NMR **3g** (400 MHz) in $CDCl_3$

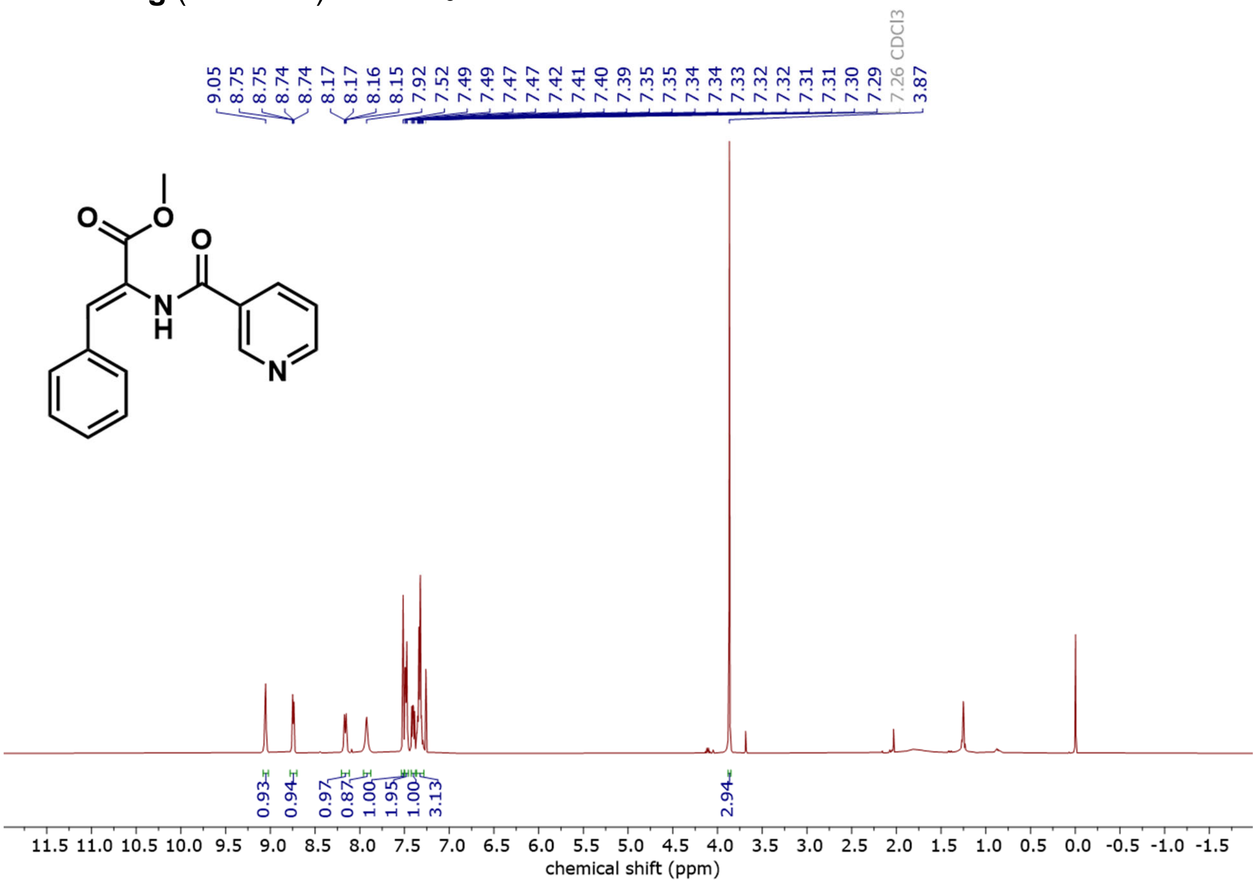

^{13}C NMR of **3g** (101 MHz) in $CDCl_3$

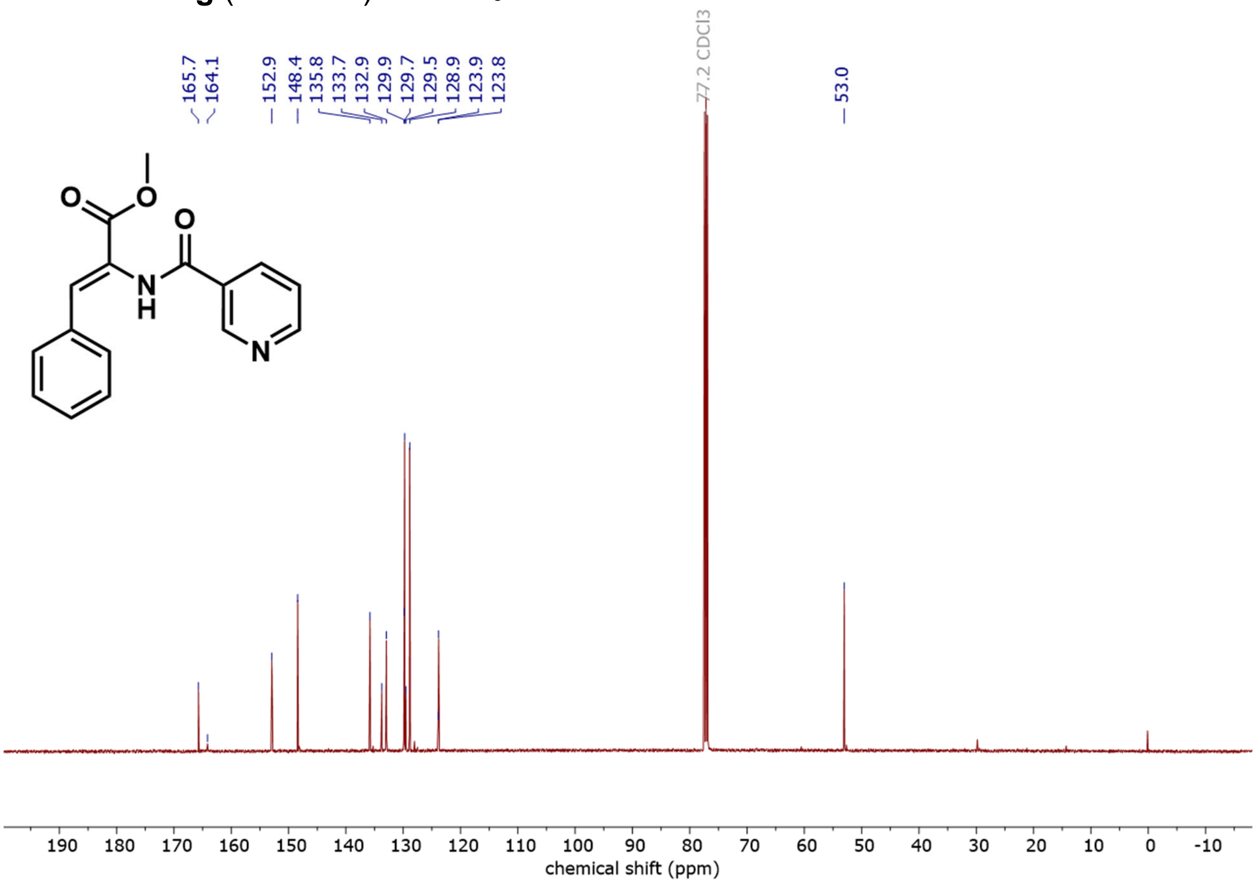

^{1}H NMR of **3h** (400 MHz) in $CDCl_3$

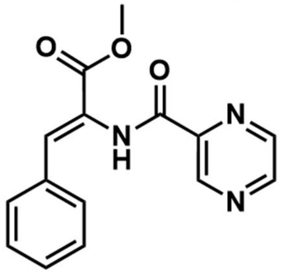

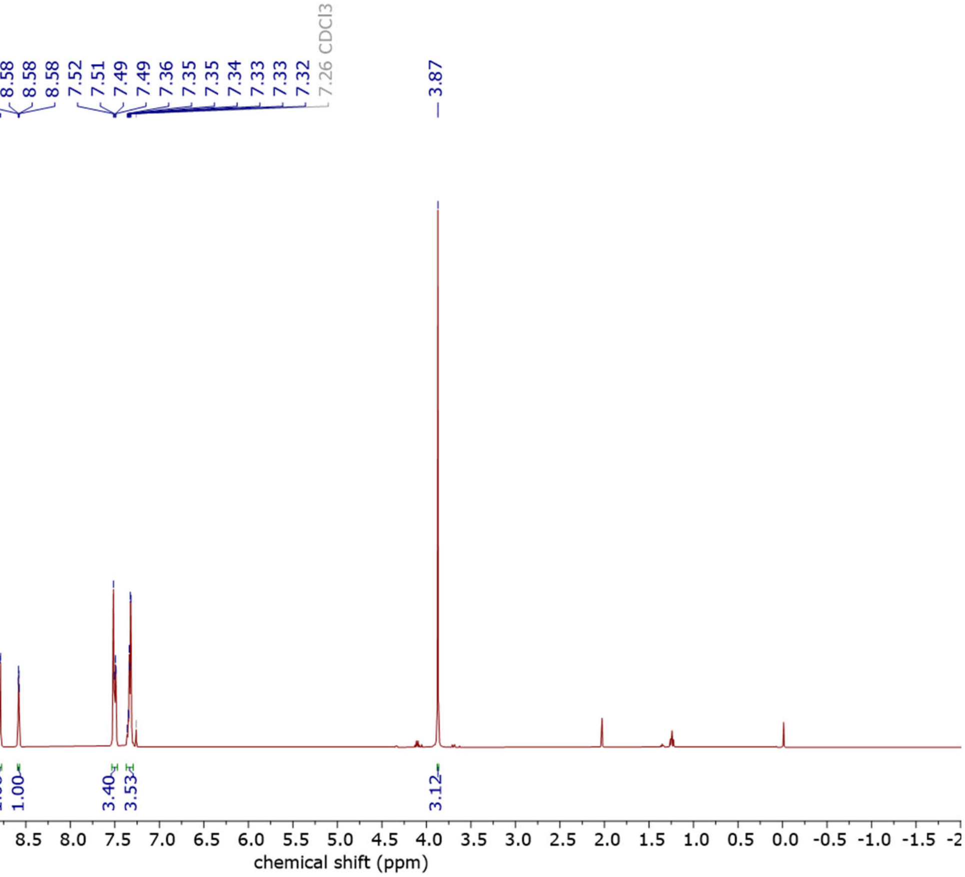

^{13}C NMR of **3h** (101 MHz) in $CDCl_3$

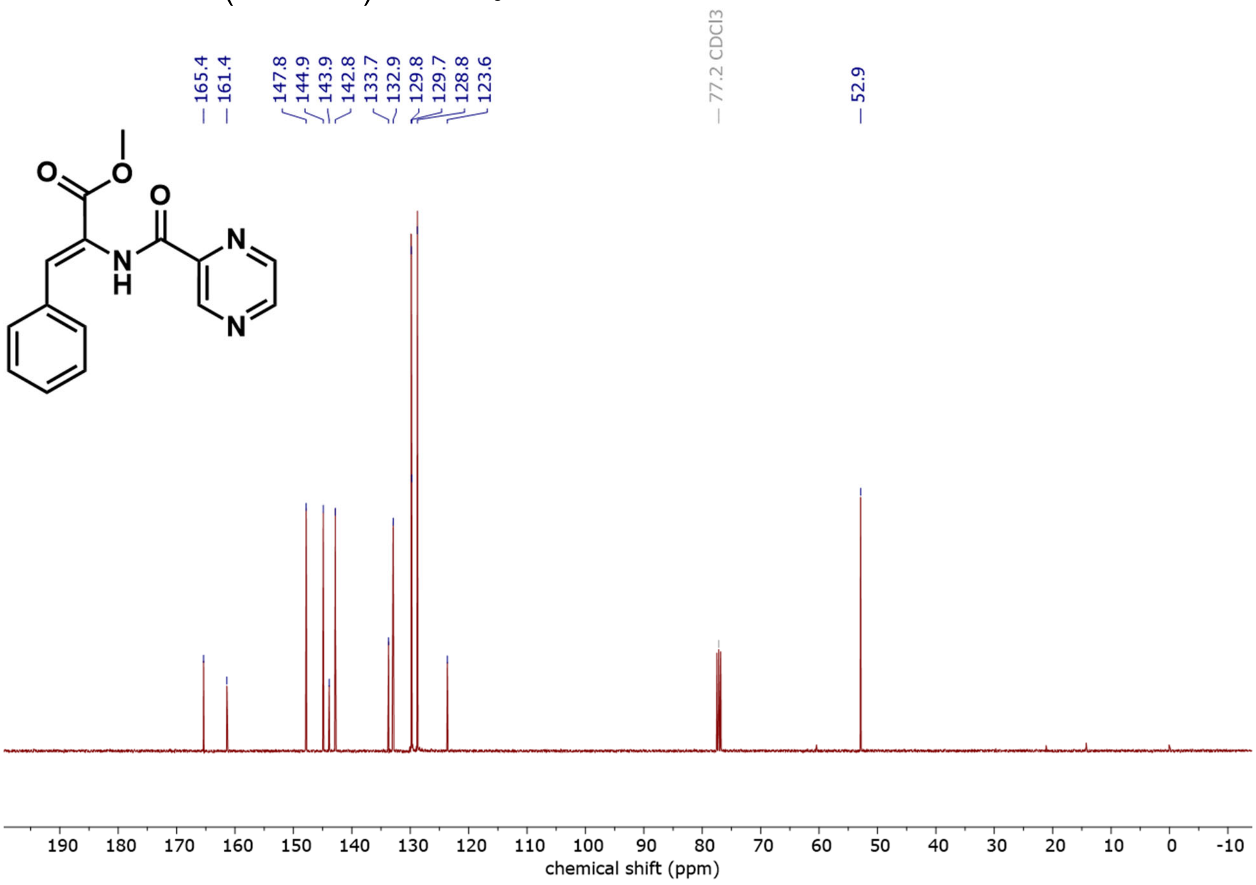

^{1}H NMR of **3i** (400 MHz) in $CDCl_3$

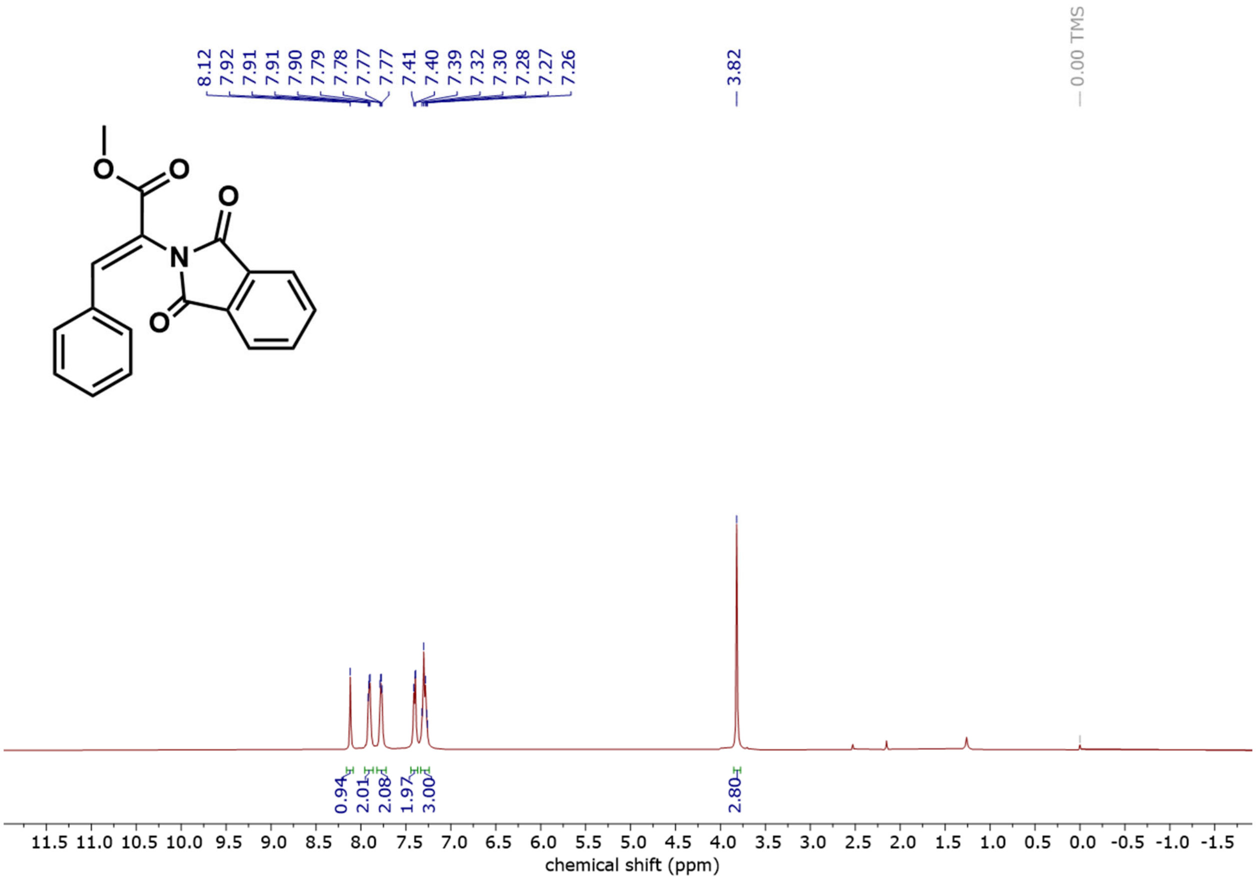

^{13}C NMR of **3i** (151 MHz) in $CDCl_3$

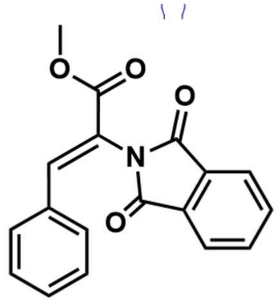

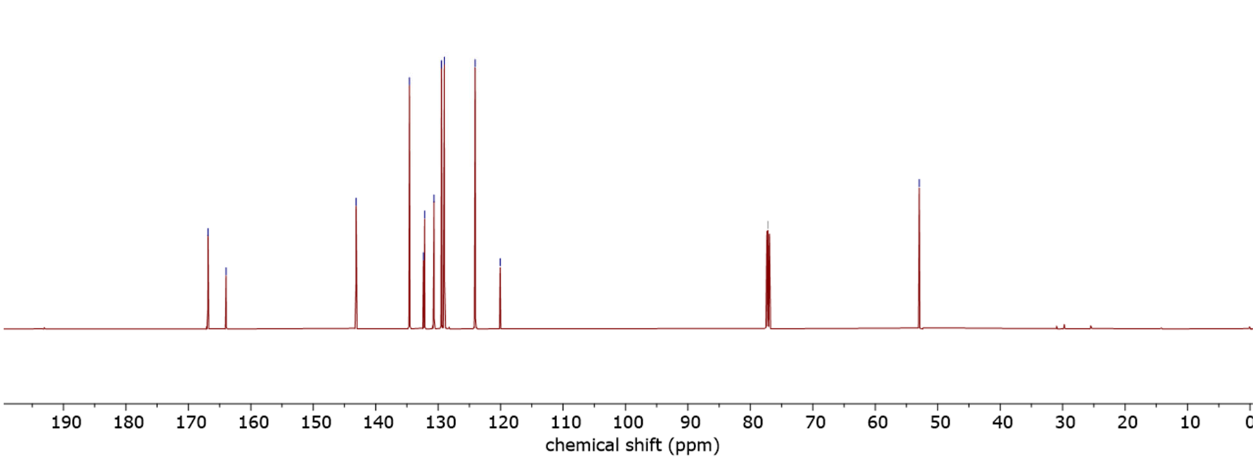

^1H NMR of **3j** (600 MHz) in $CDCl_3$

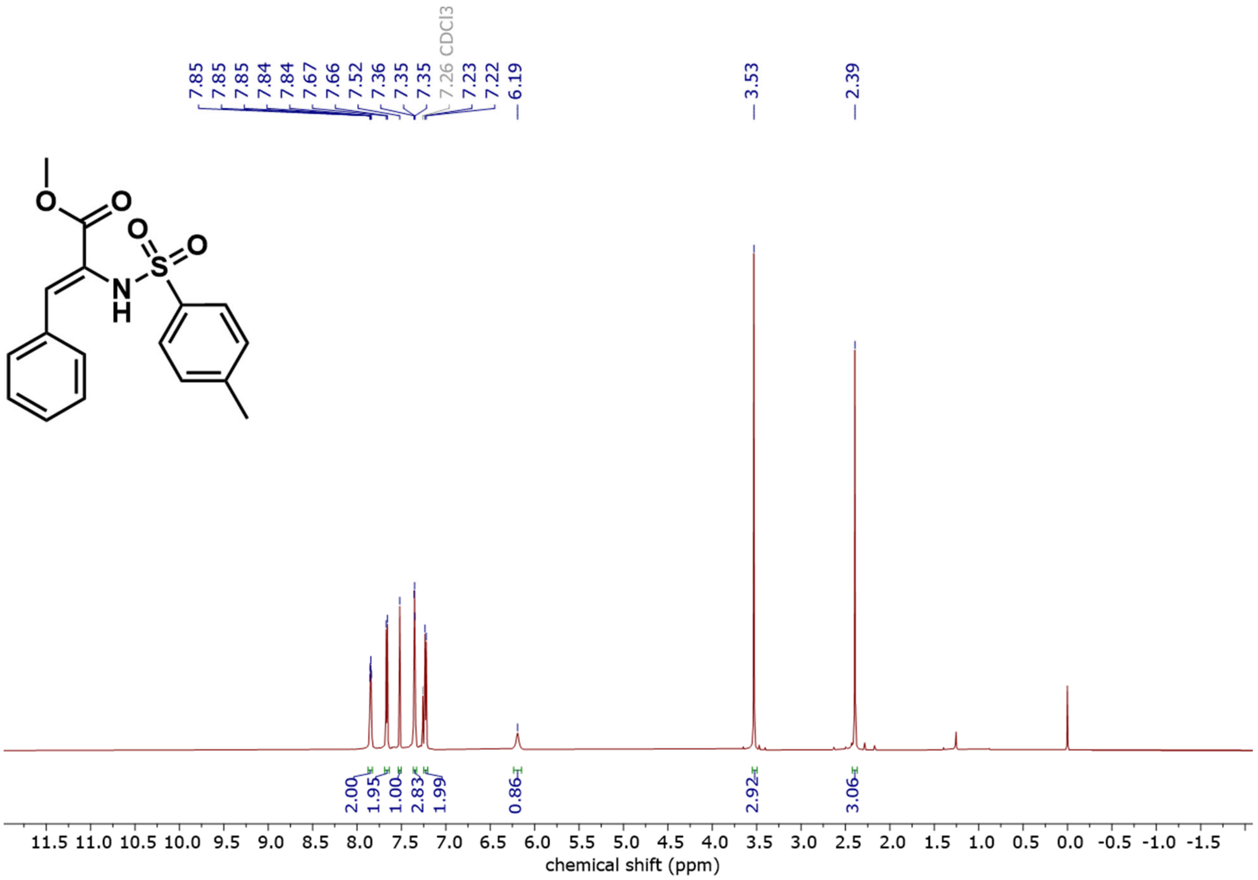

^{13}C NMR of **3j** (151 MHz) in $CDCl_3$

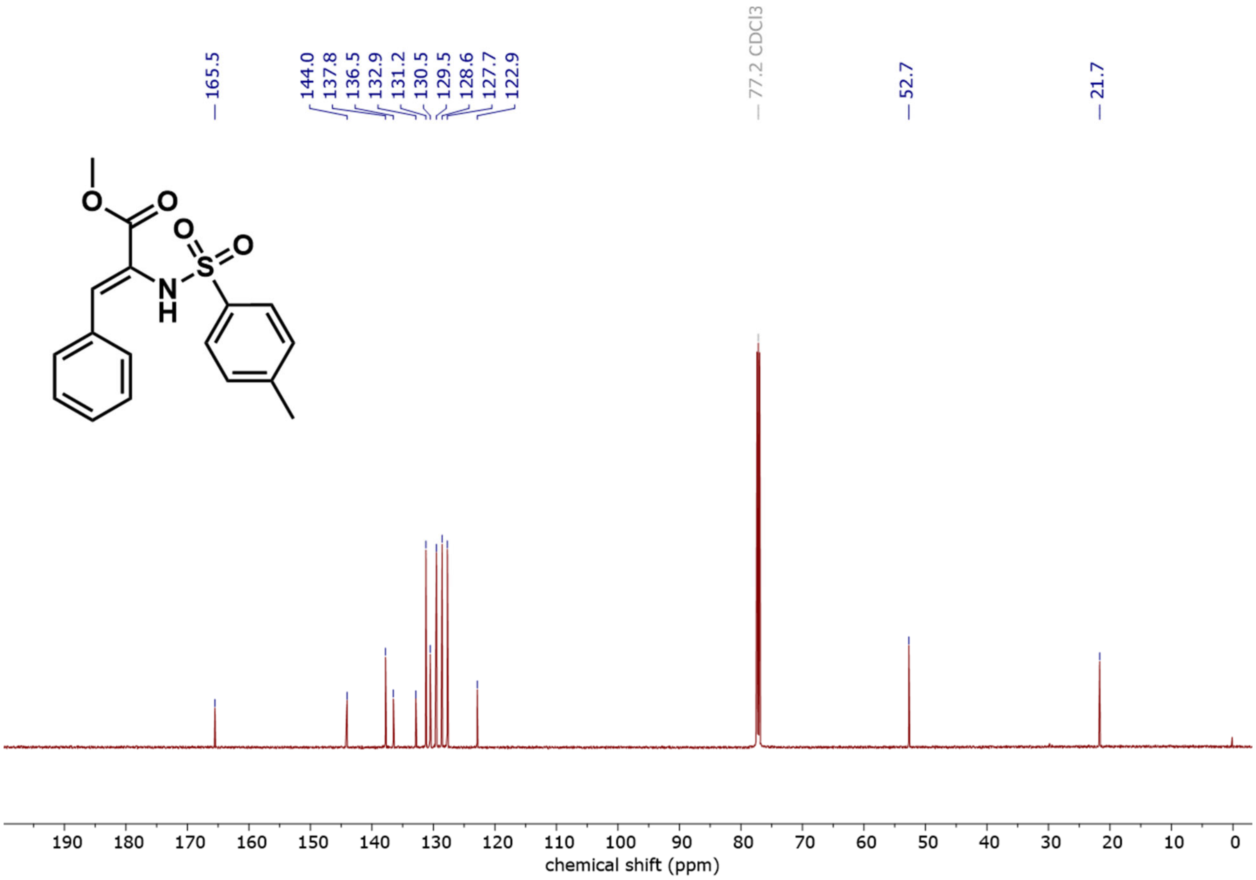

^{1}H NMR of **3k** (400 MHz) in $CDCl_3$

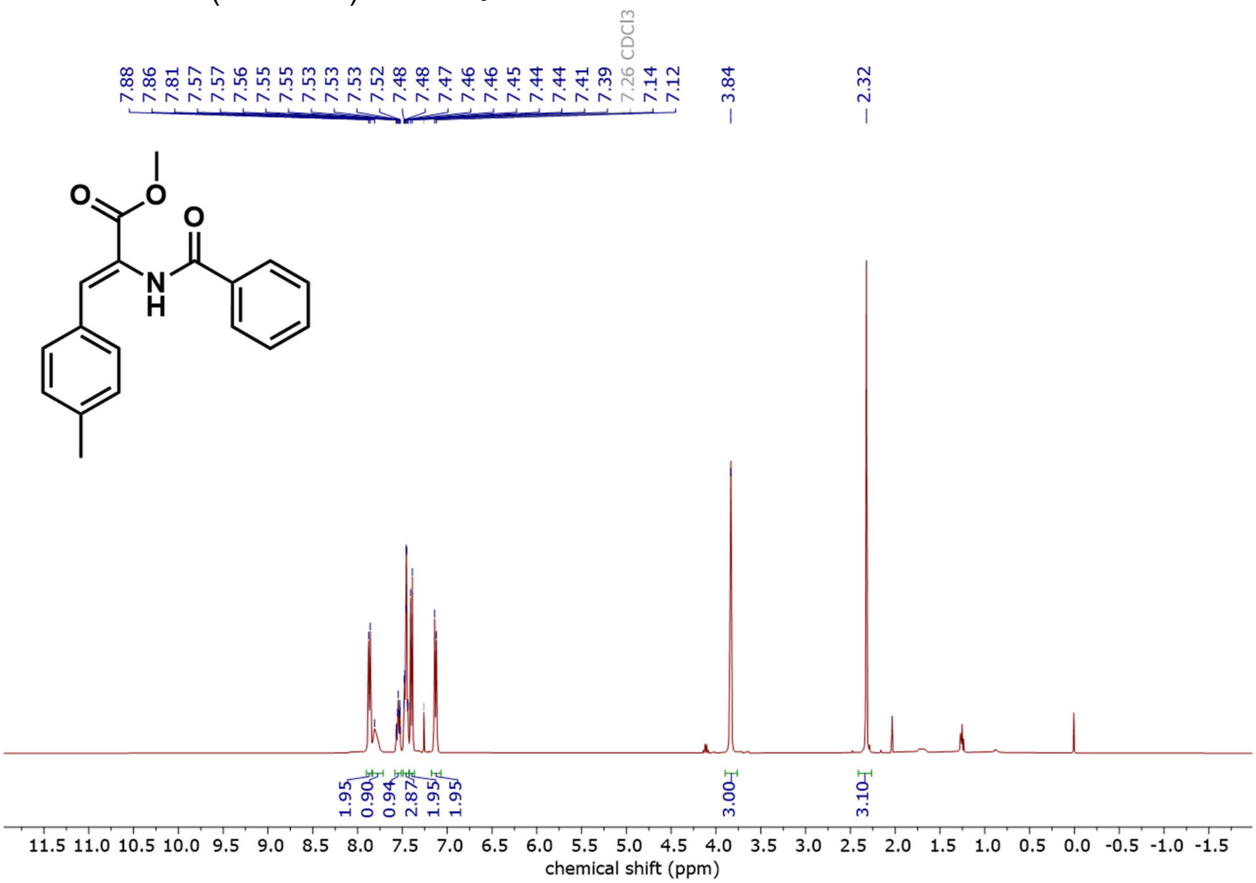

^{13}C NMR of **3k**(101 MHz) in $CDCl_3$

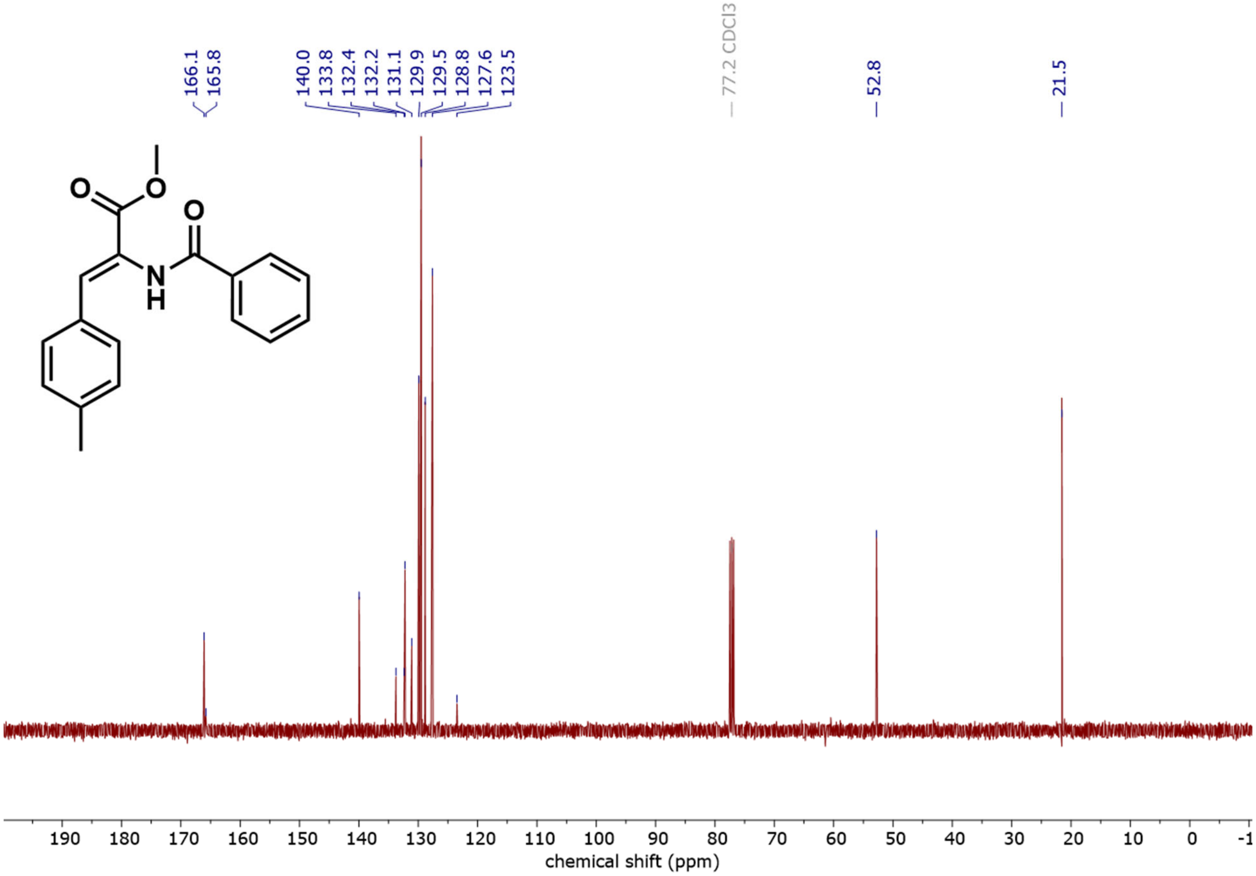

^{1}H NMR of **3l** (400 MHz) in $CDCl_3$

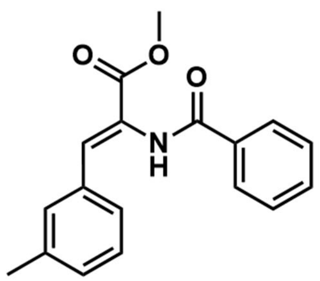

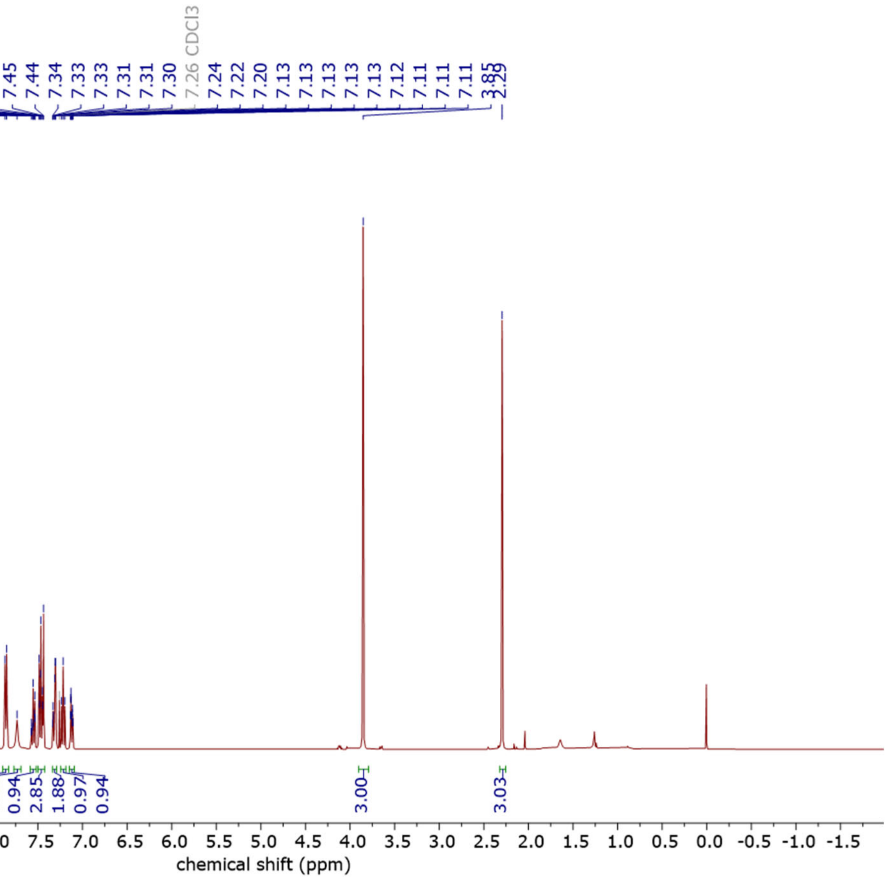

^{13}C NMR of **3l** (101MHz) in $CDCl_3$

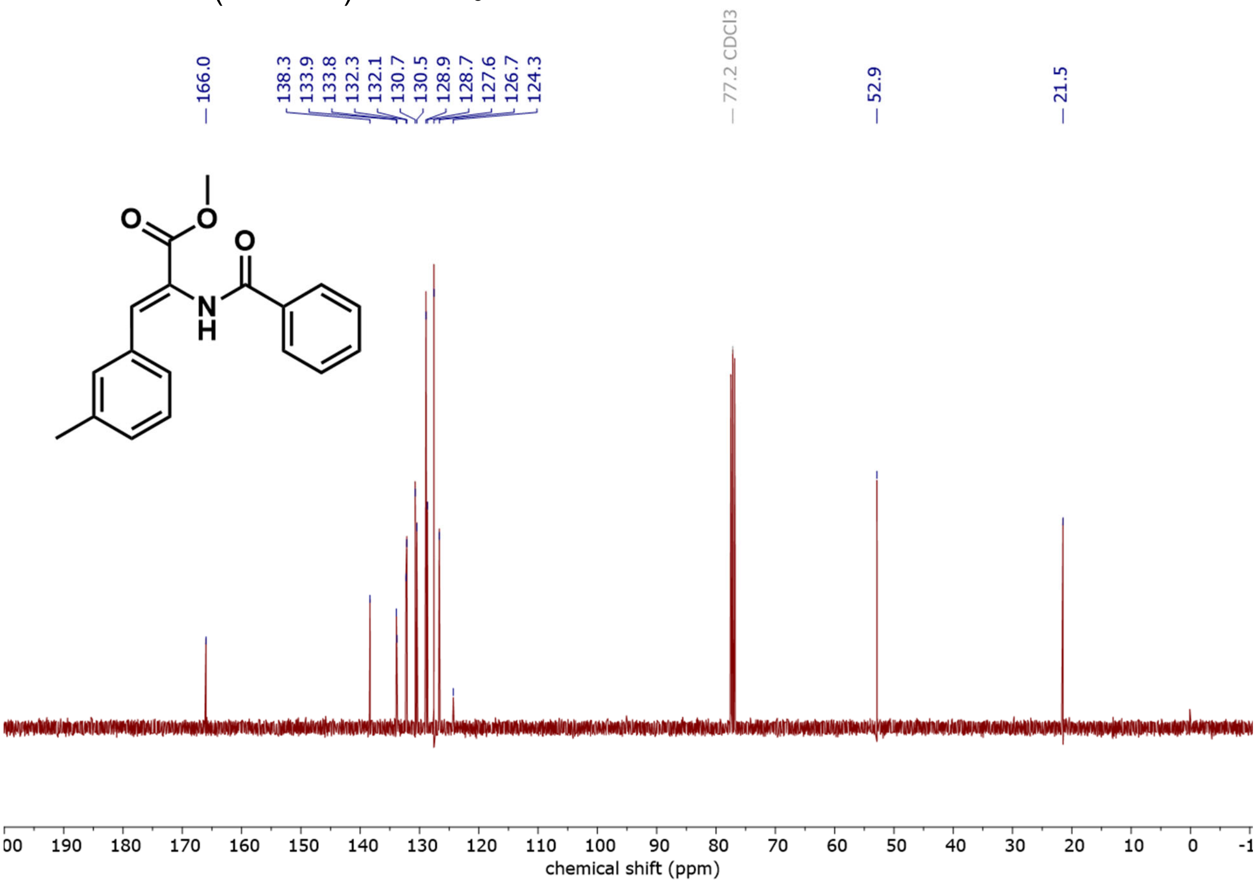

^{1}H NMR of **3m** (400 MHz) in $CDCl_3$

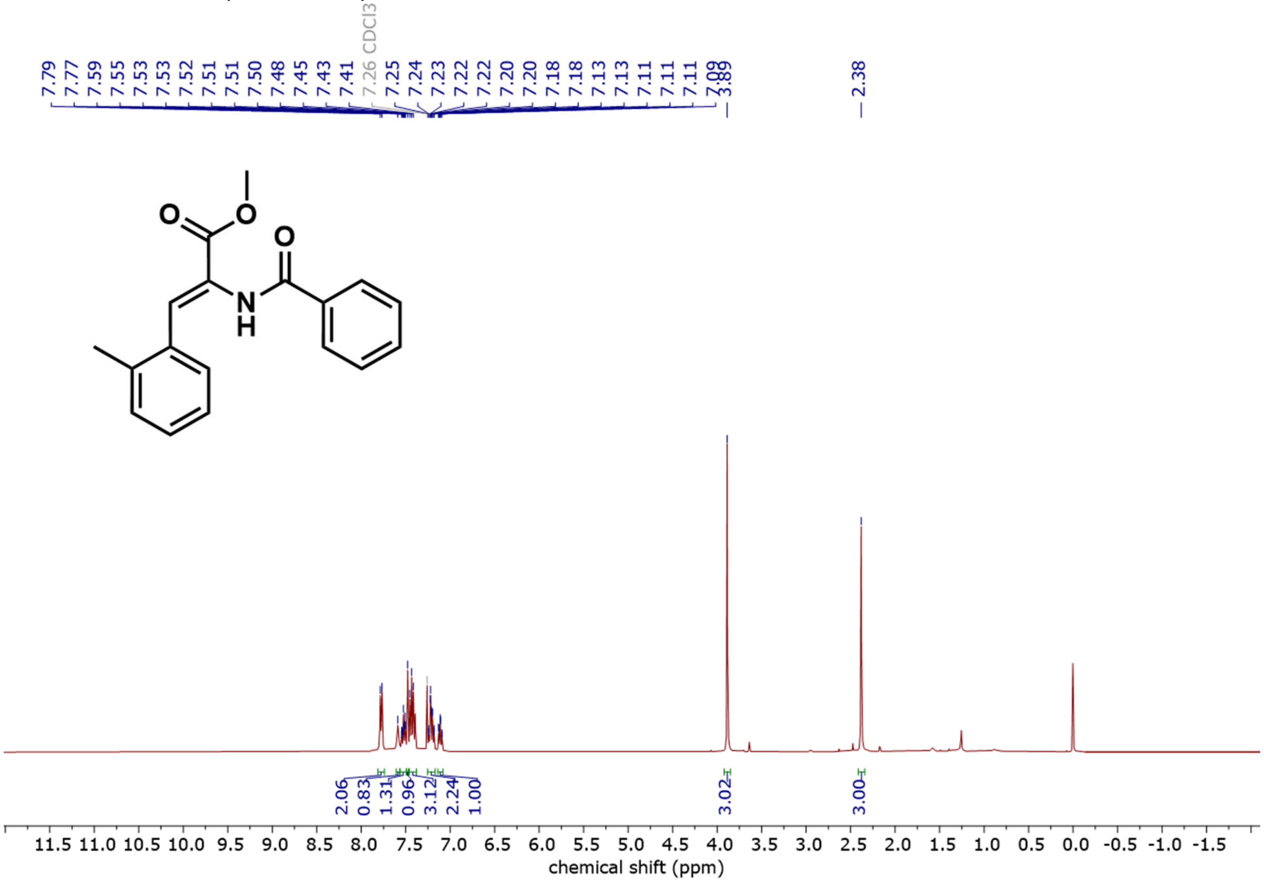

^{13}C NMR of **3m** (101 MHz) in $CDCl_3$

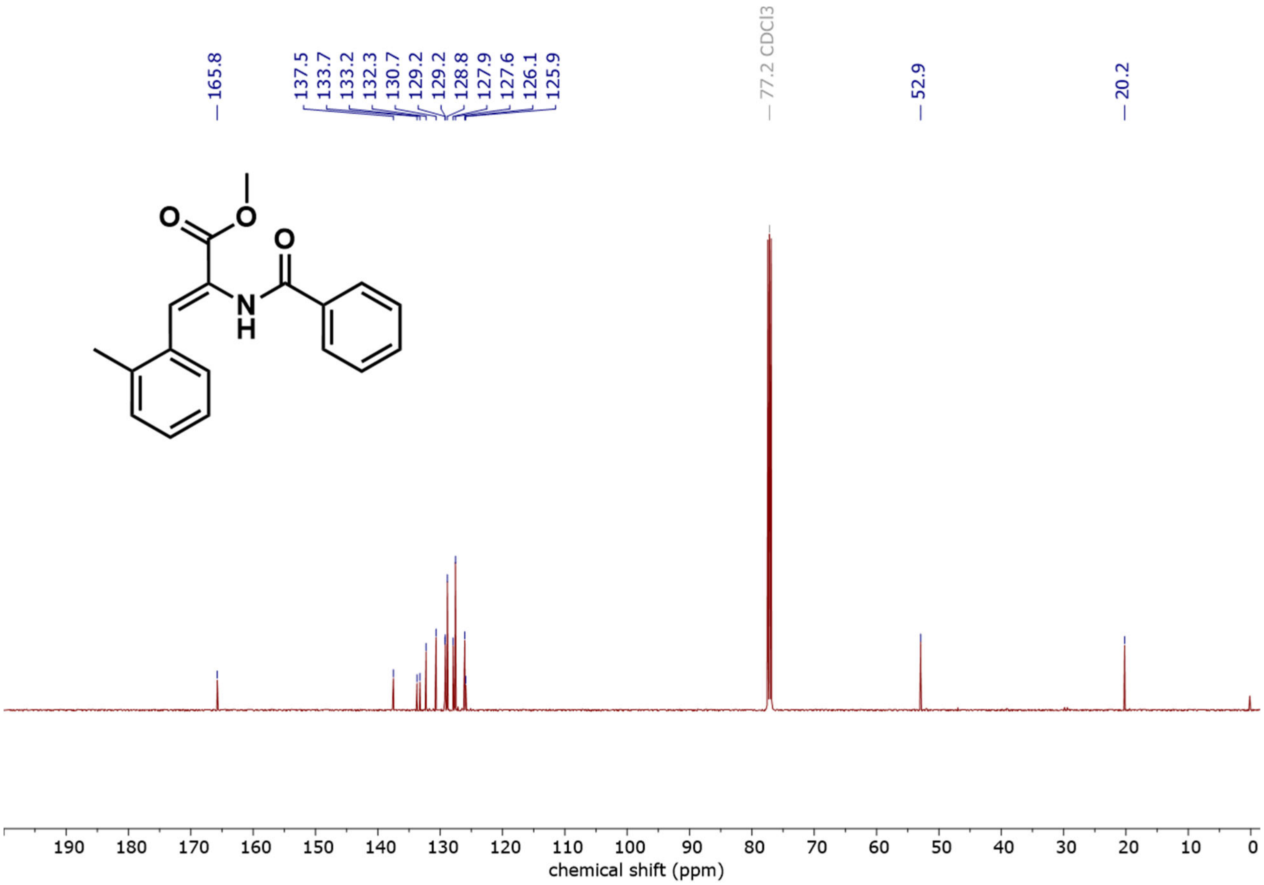

^{1}H NMR of **3n** (400 MHz) in $CDCl_3$

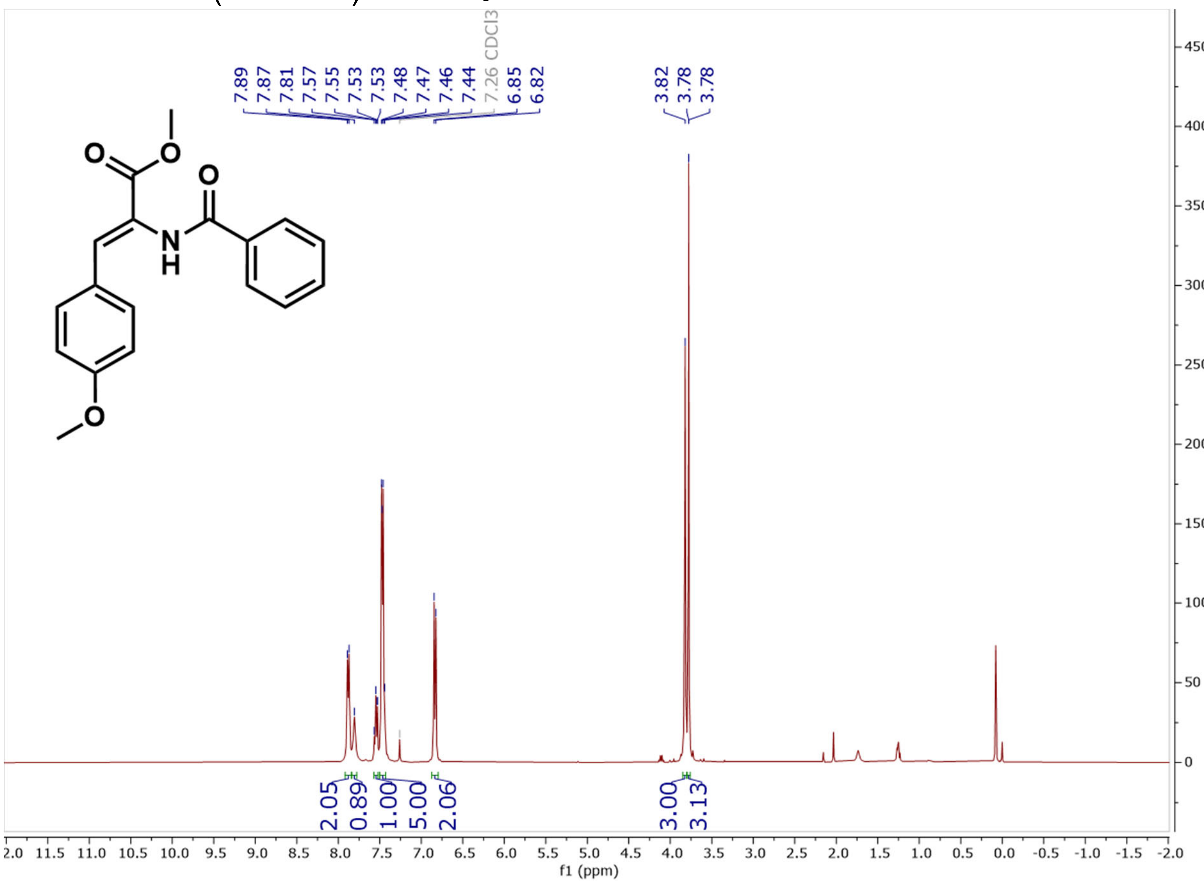

^{13}C NMR of **3n** (101 MHz) in $CDCl_3$

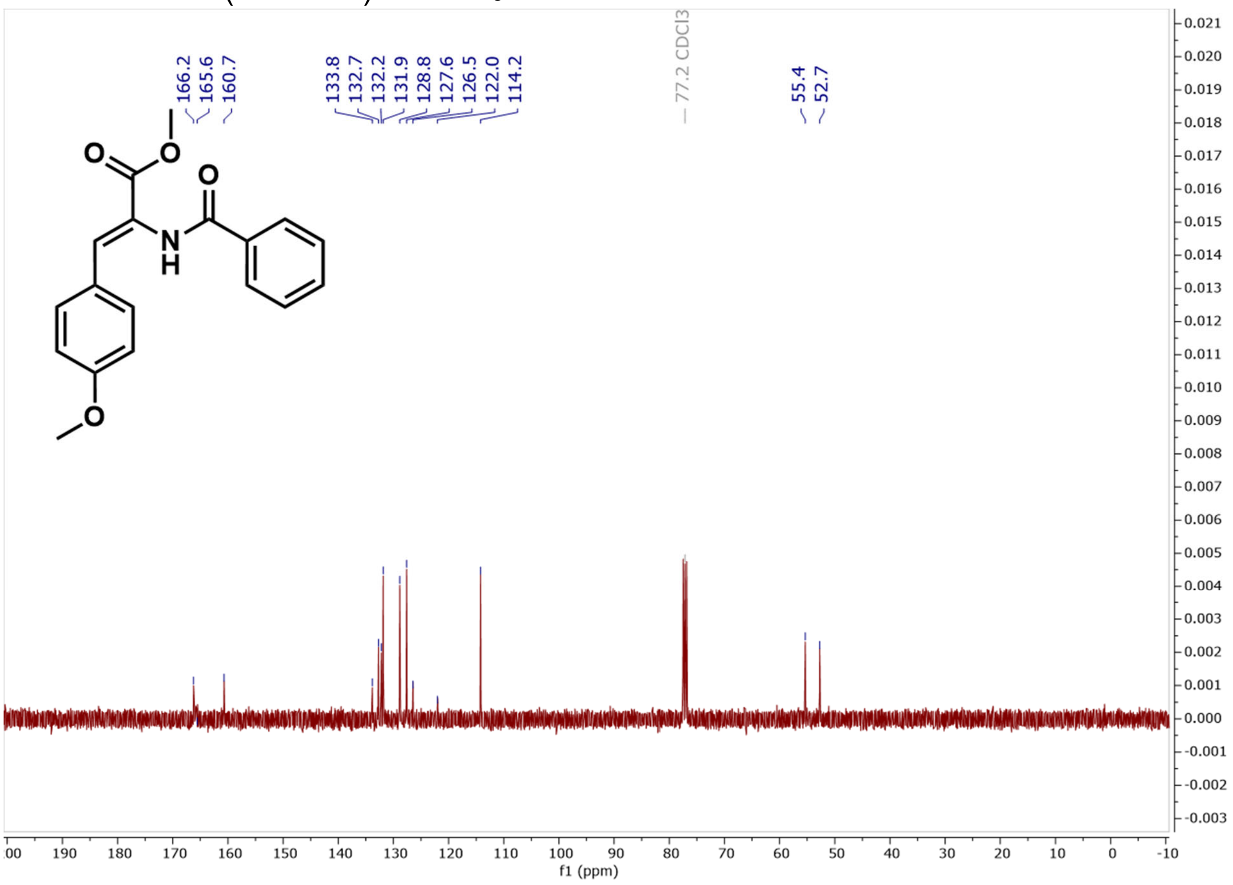

^{1}H NMR of **3o** (400 MHz) in $CDCl_3$

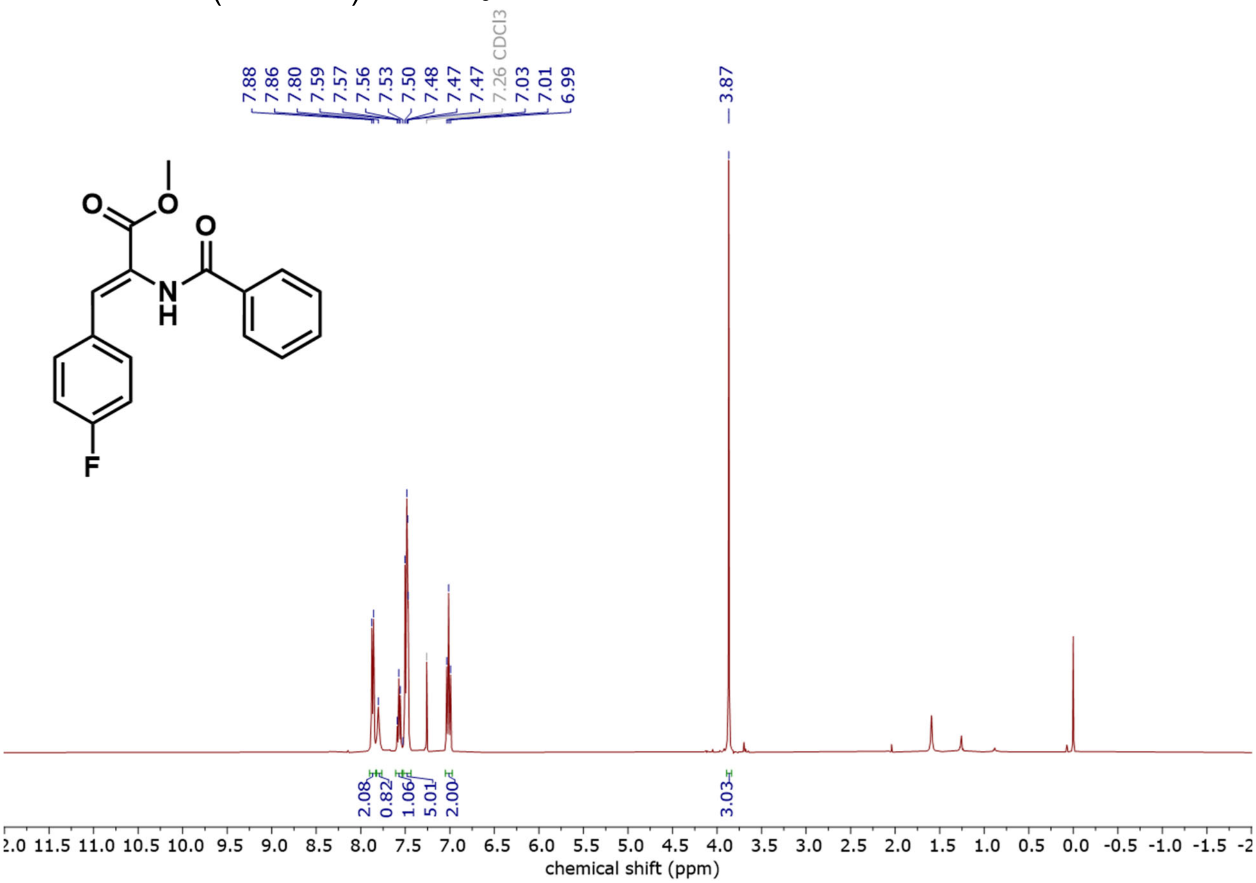

^{13}C NMR of **3o** (101 MHz) in $CDCl_3$

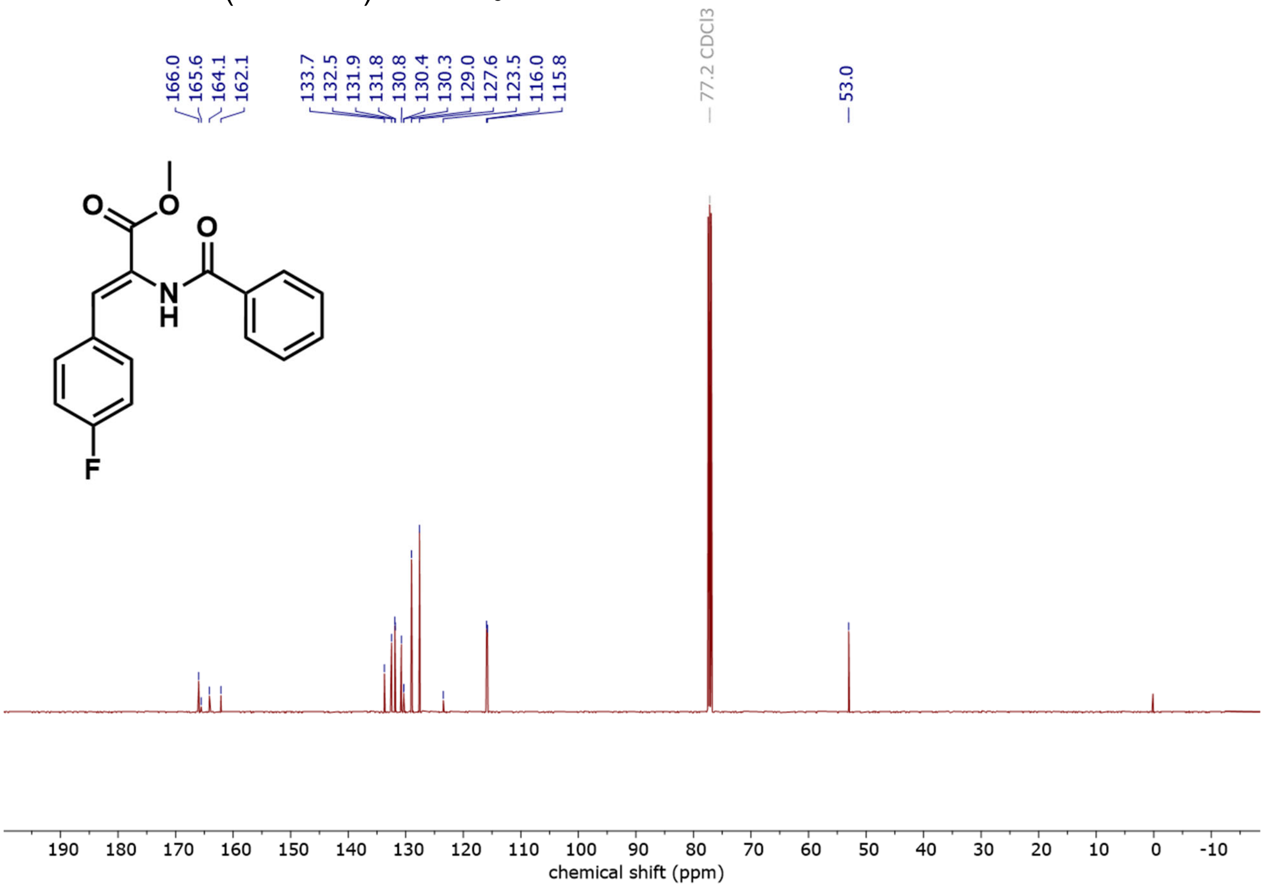

^{19}F NMR of **3o** (376 MHz) in $CDCl_3$

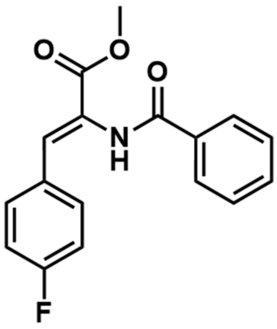

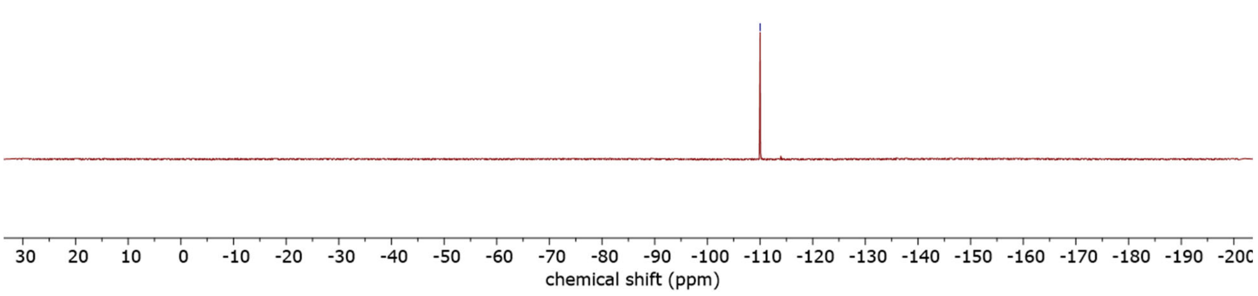

^{1}H NMR of **3p** (400 MHz) in $CDCl_3$

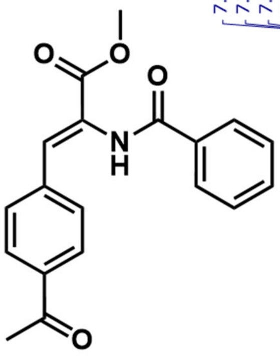

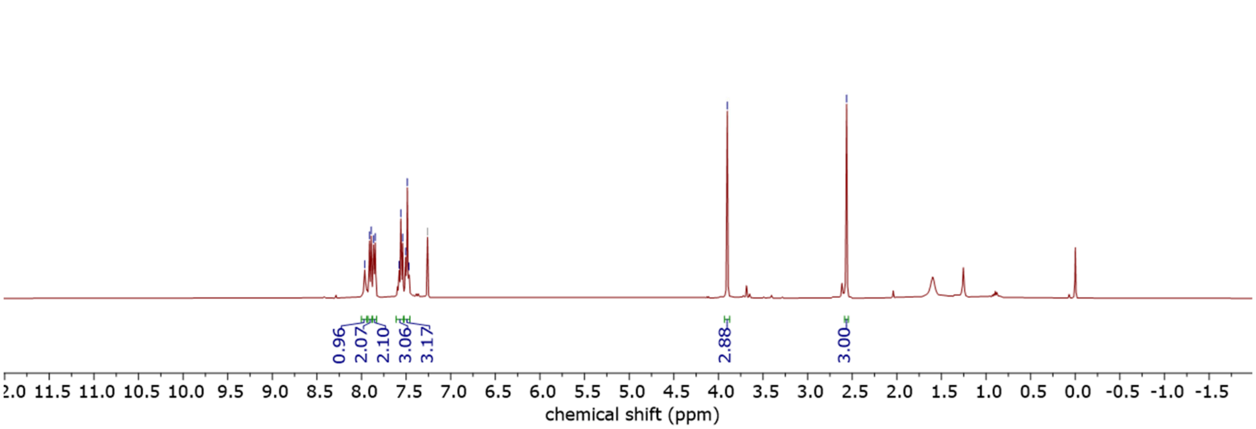

^{13}C NMR of **3p** (101 MHz) in $CDCl_3$

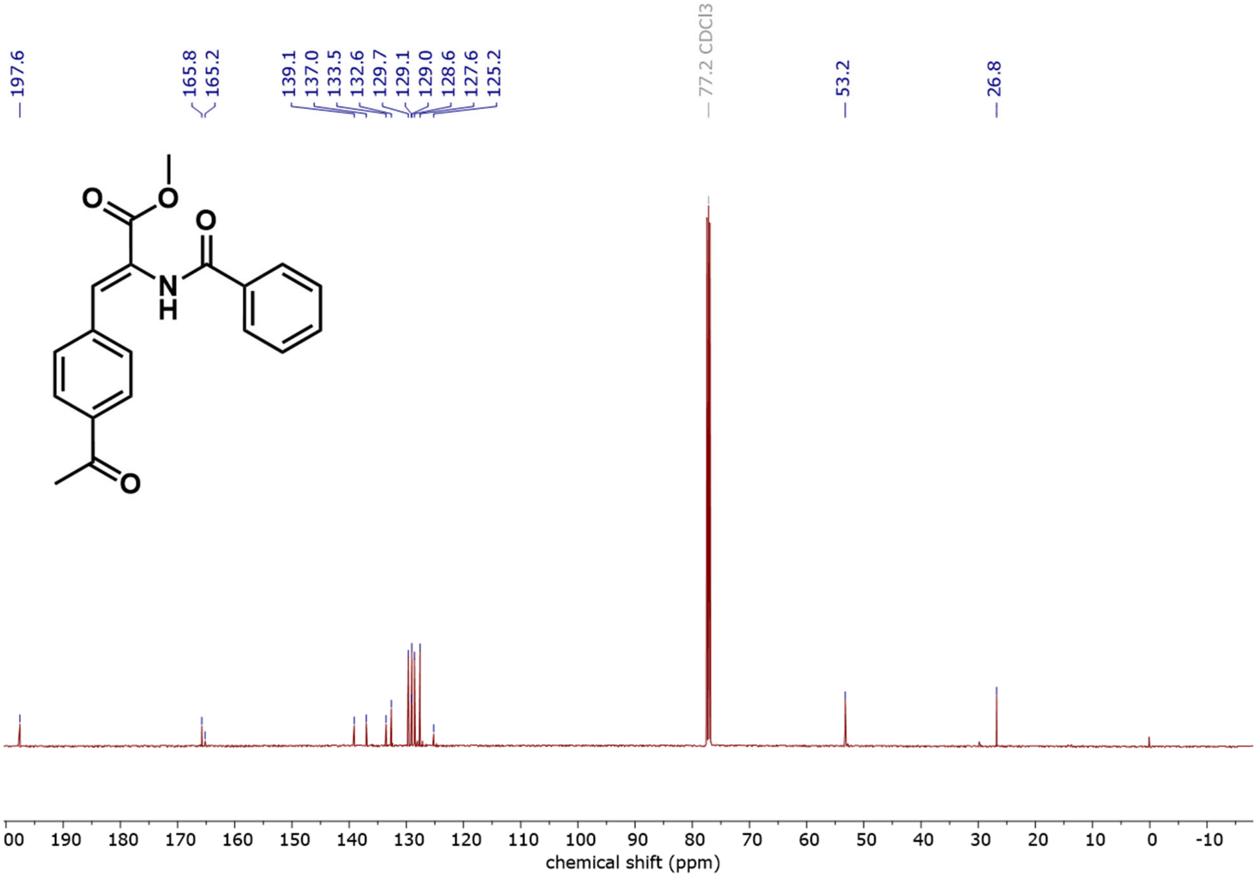

^{1}H NMR of **3q** (400 MHz) in $CDCl_3$

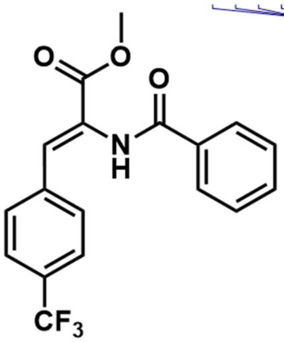

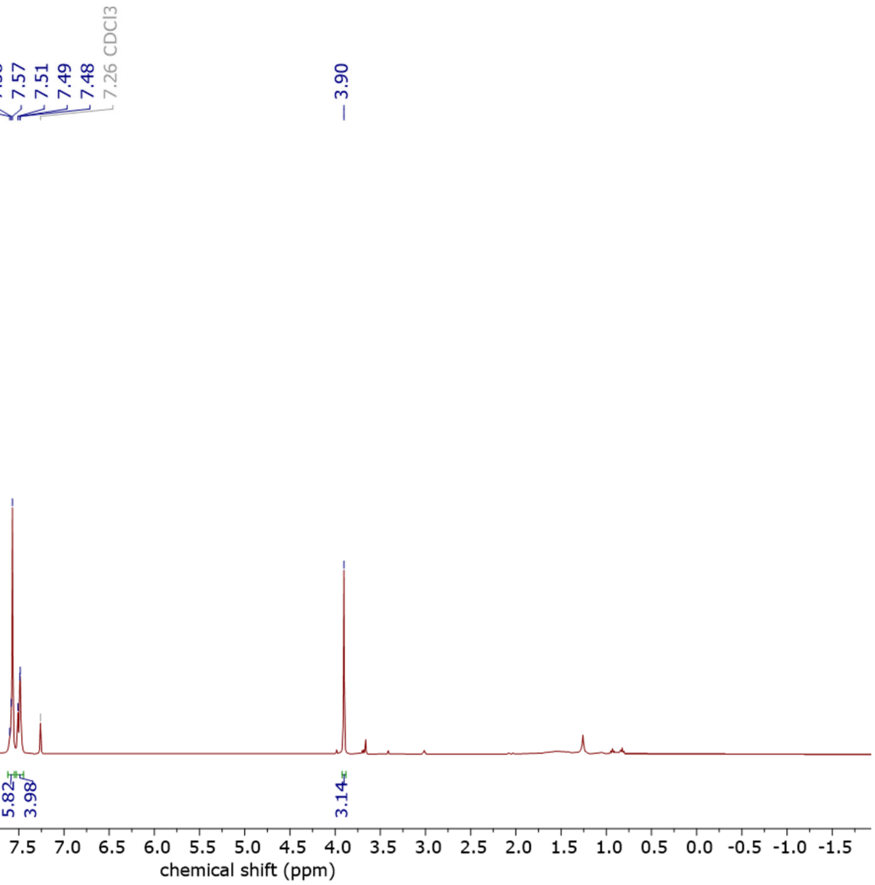

^{13}C NMR of **3q** (126 MHz) in $CDCl_3$

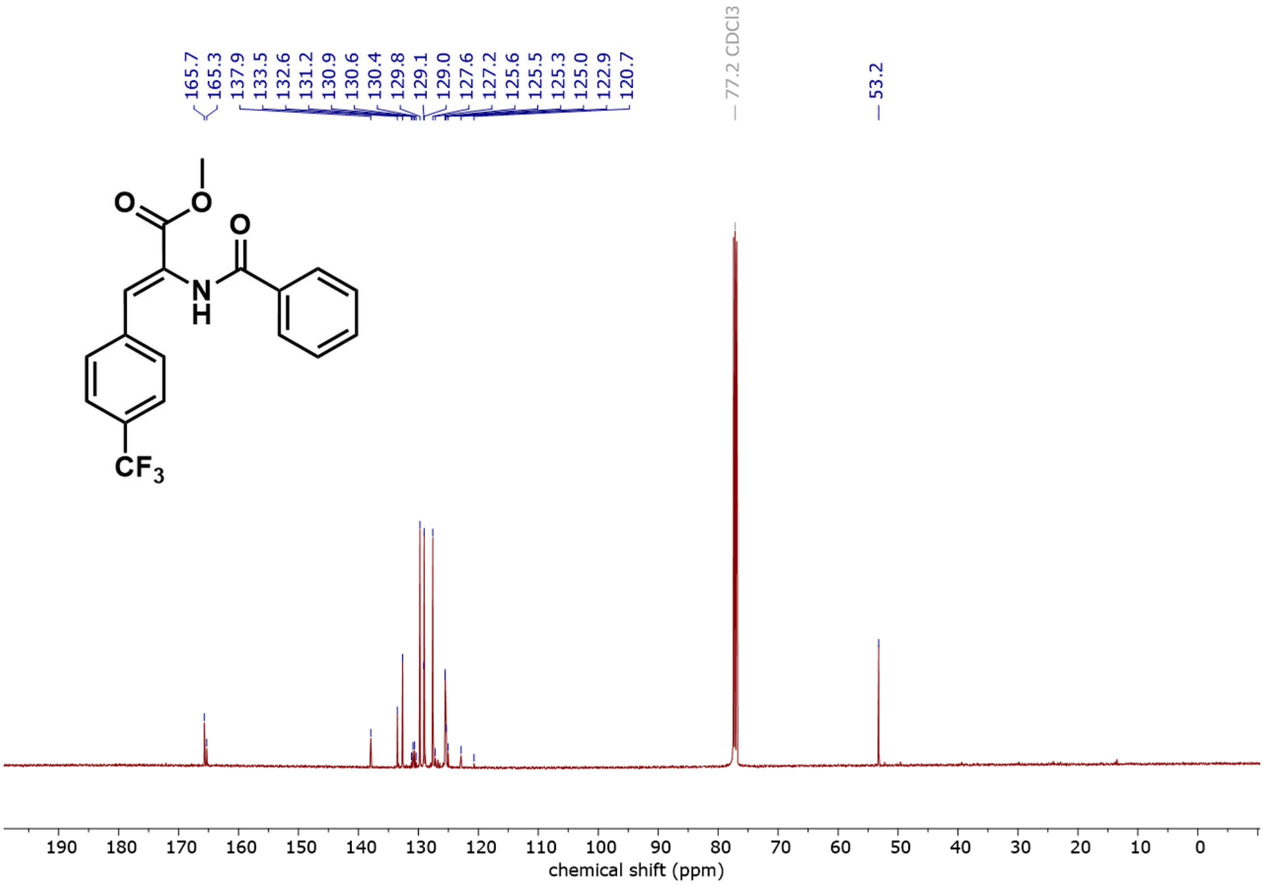

^{19}F NMR of **3q** (376 MHz) in $CDCl_3$

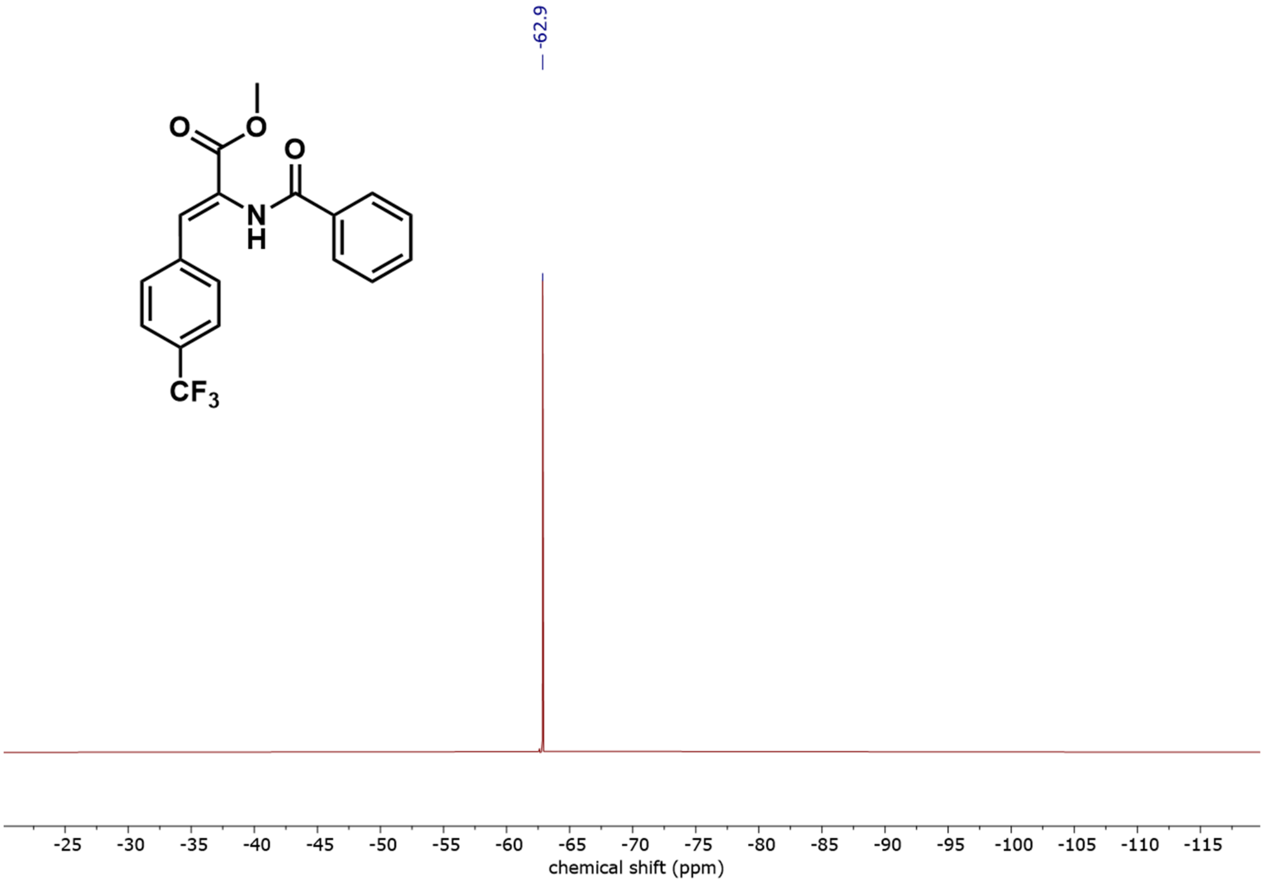

^{1}H NMR of **3r** (400 MHz) in $CDCl_3$

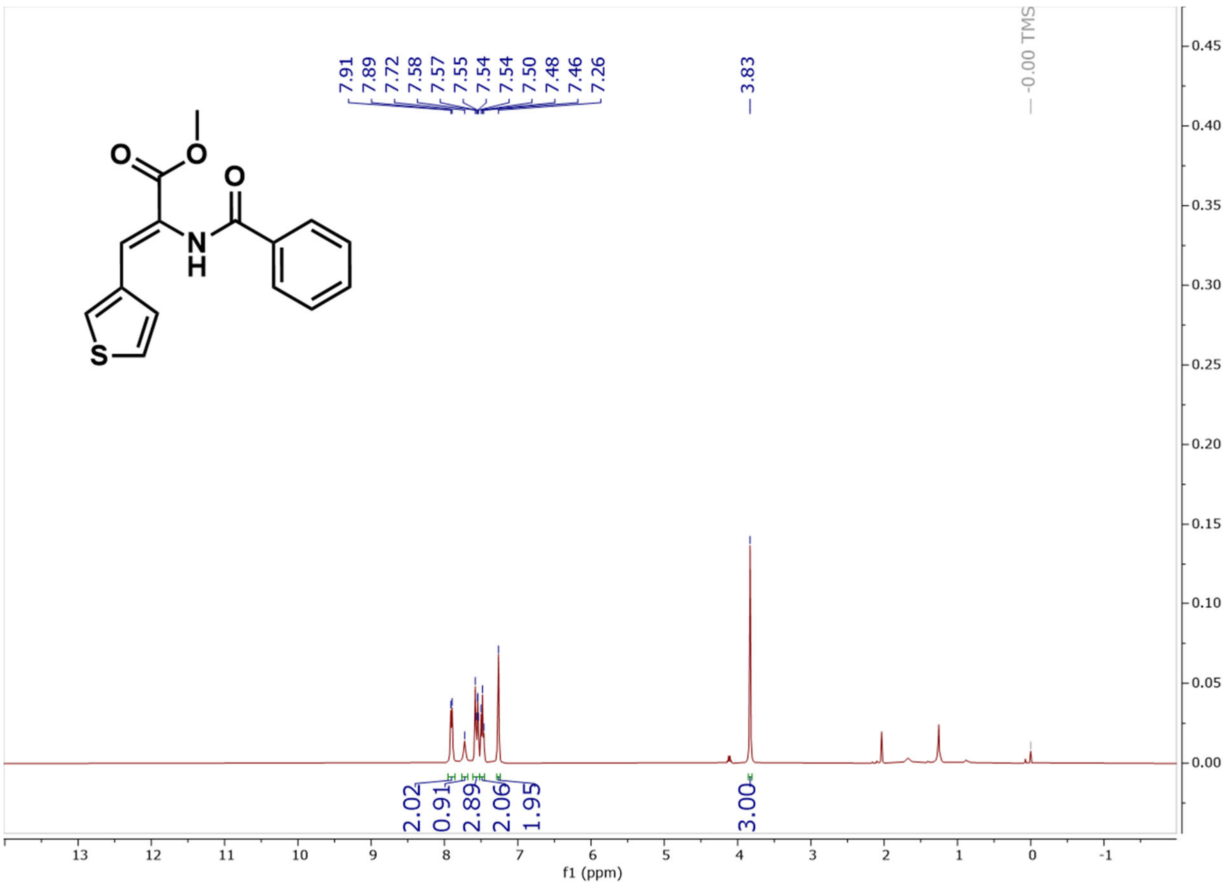

^{13}C NMR of **3r** (126 MHz) in $CDCl_3$

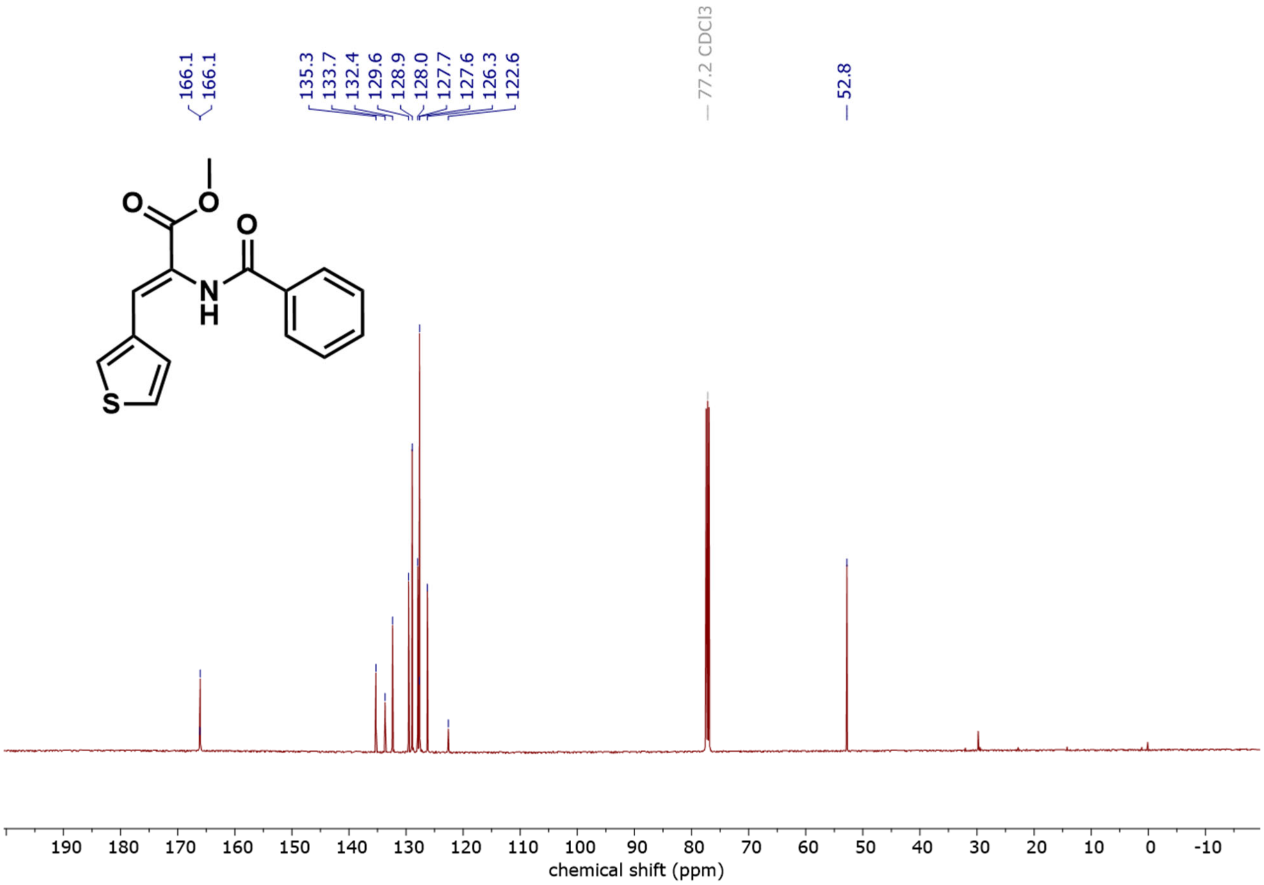

^{1}H NMR of **3s** (500 MHz) in $CDCl_3$

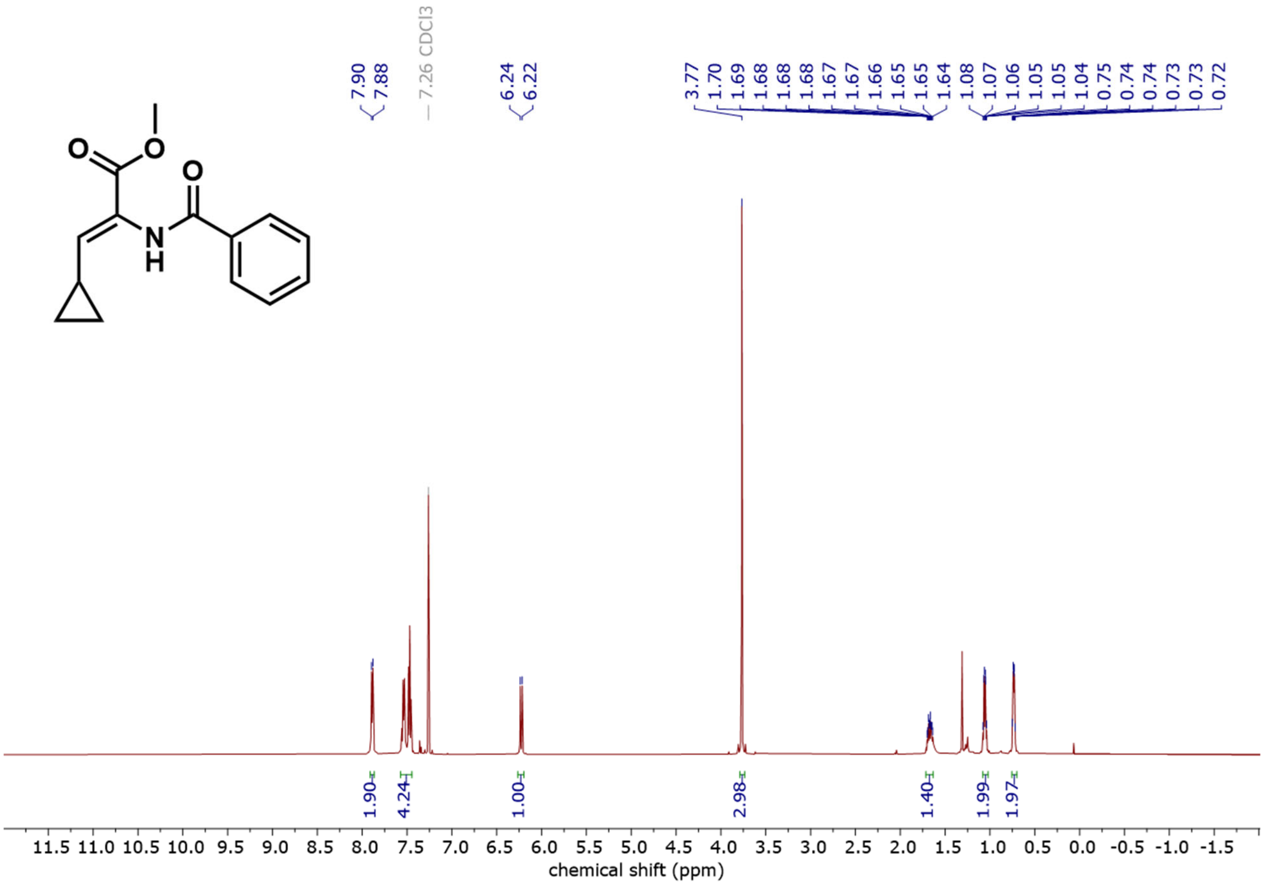

^{13}C NMR of **3s** (126 MHz) in $CDCl_3$

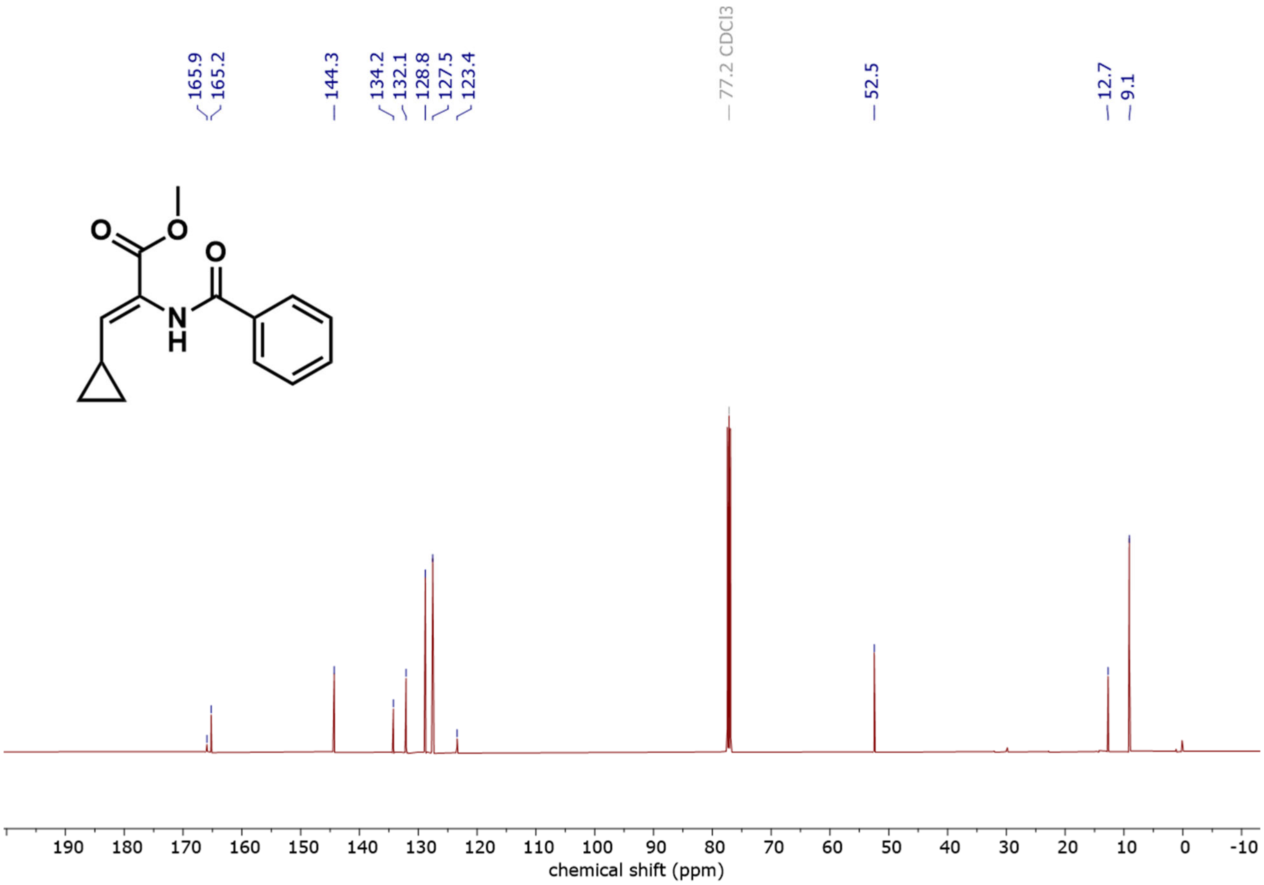

^1H NMR of **3t** (400 MHz) in $CDCl_3$

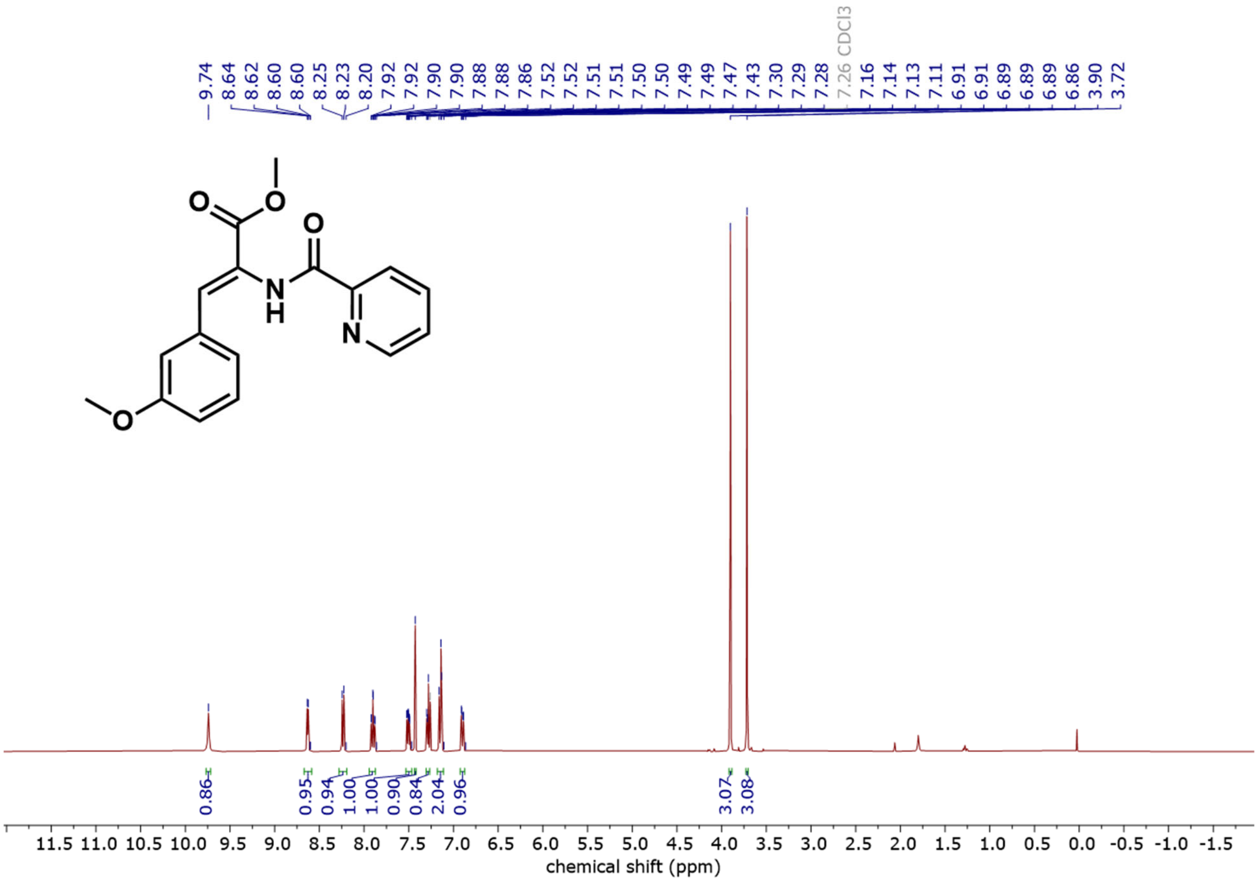

^{13}C NMR of **3t** (101 MHz) in $CDCl_3$

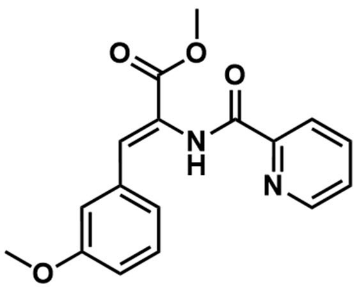

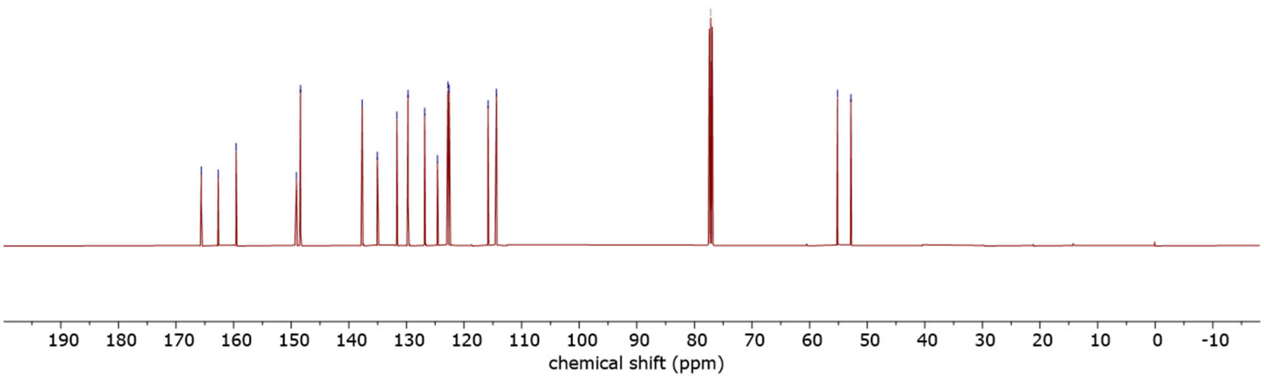

^{1}H NMR of **3u** (400 MHz) in $CDCl_3$

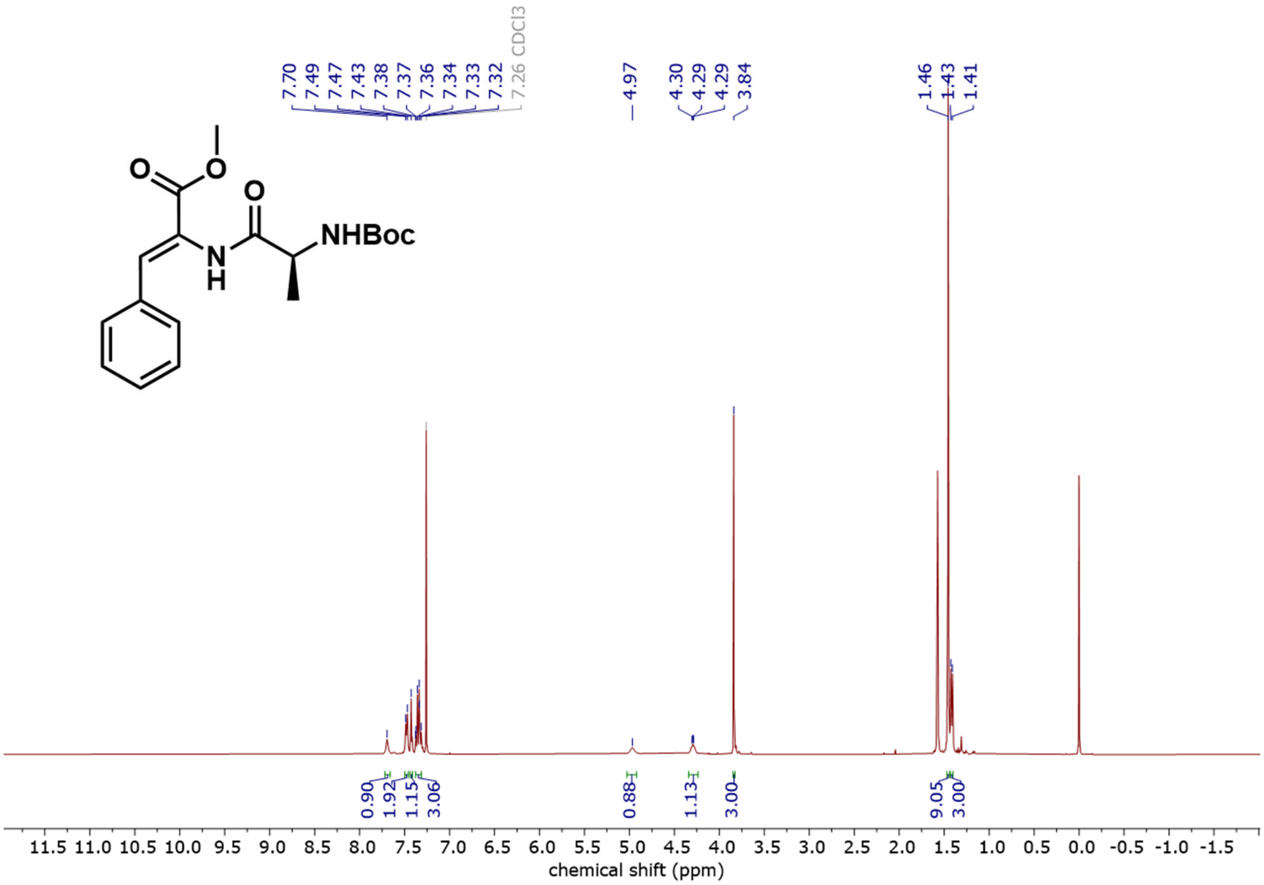

^{13}C NMR of **3u** (101 MHz) in $CDCl_3$

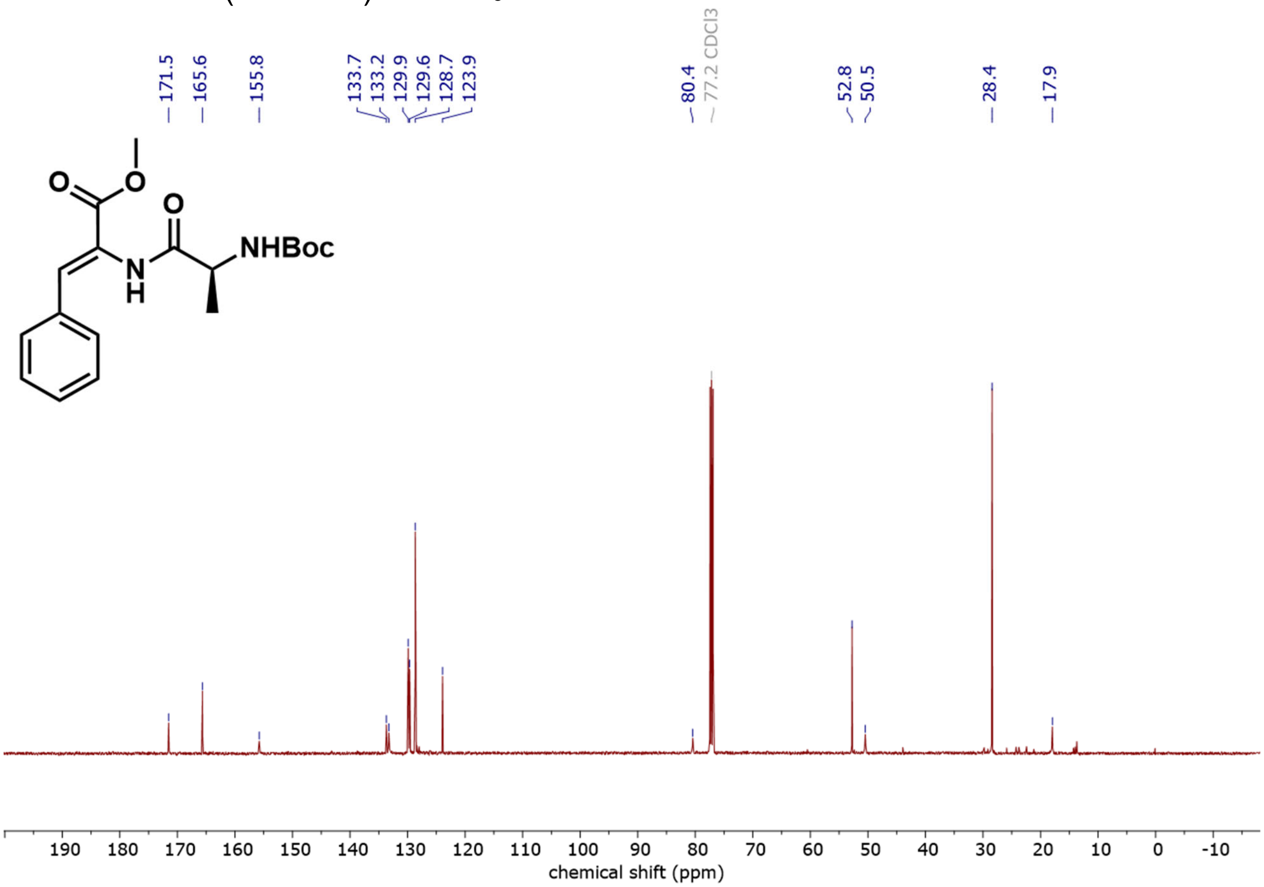

^{1}H NMR of **3v** (400 MHz) in $CDCl_3$

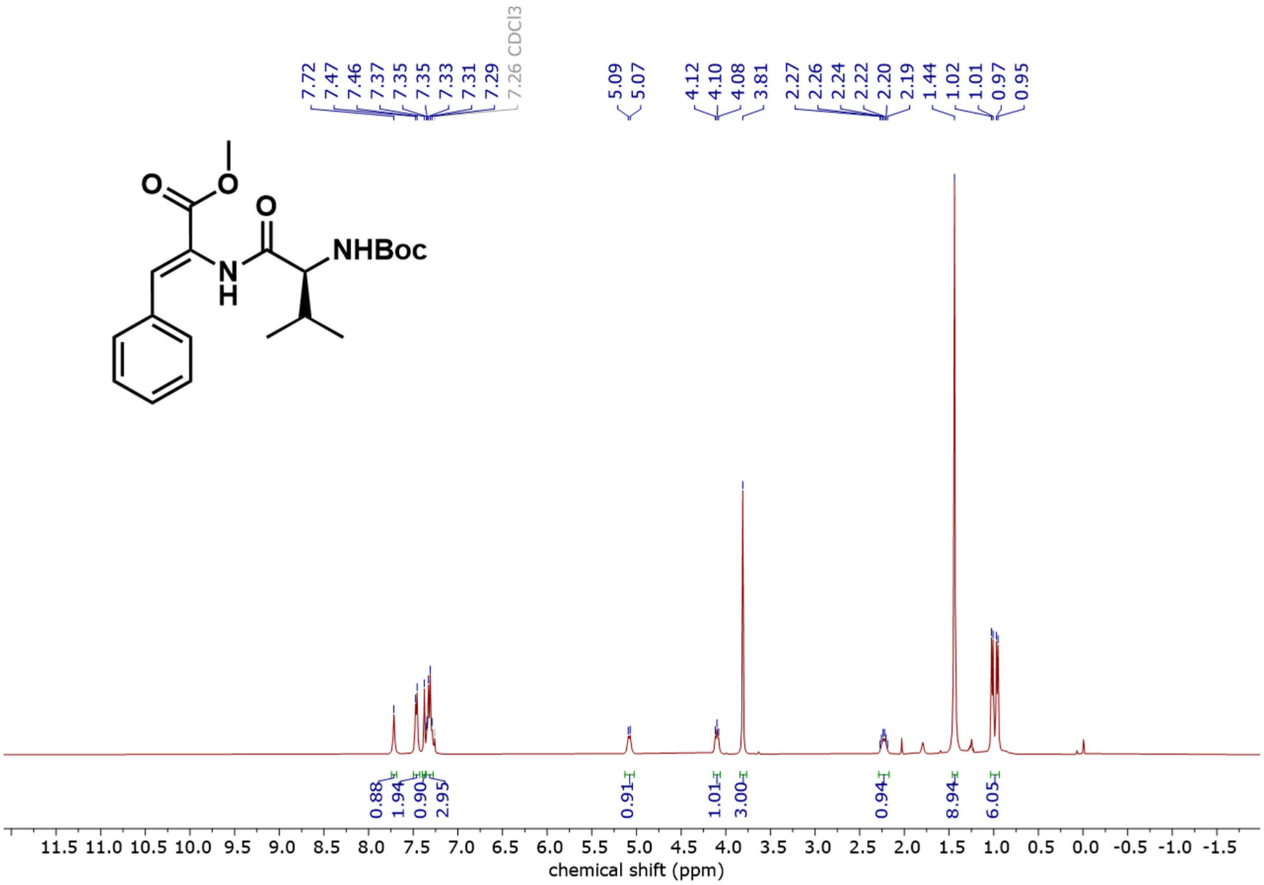

^{13}C NMR of **3v** (101 MHz) in $CDCl_3$

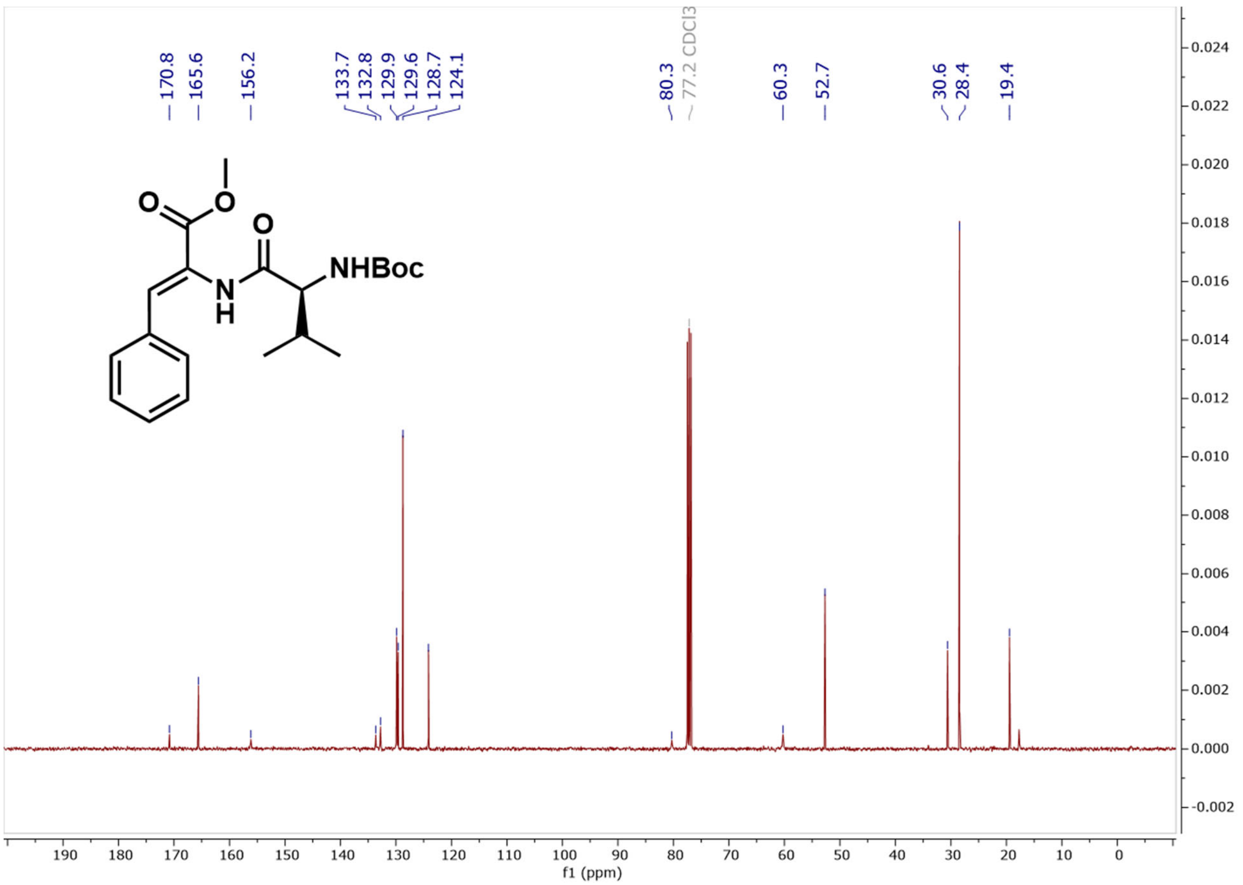

^{1}H NMR of **3w** (400 MHz) in CD_3OD

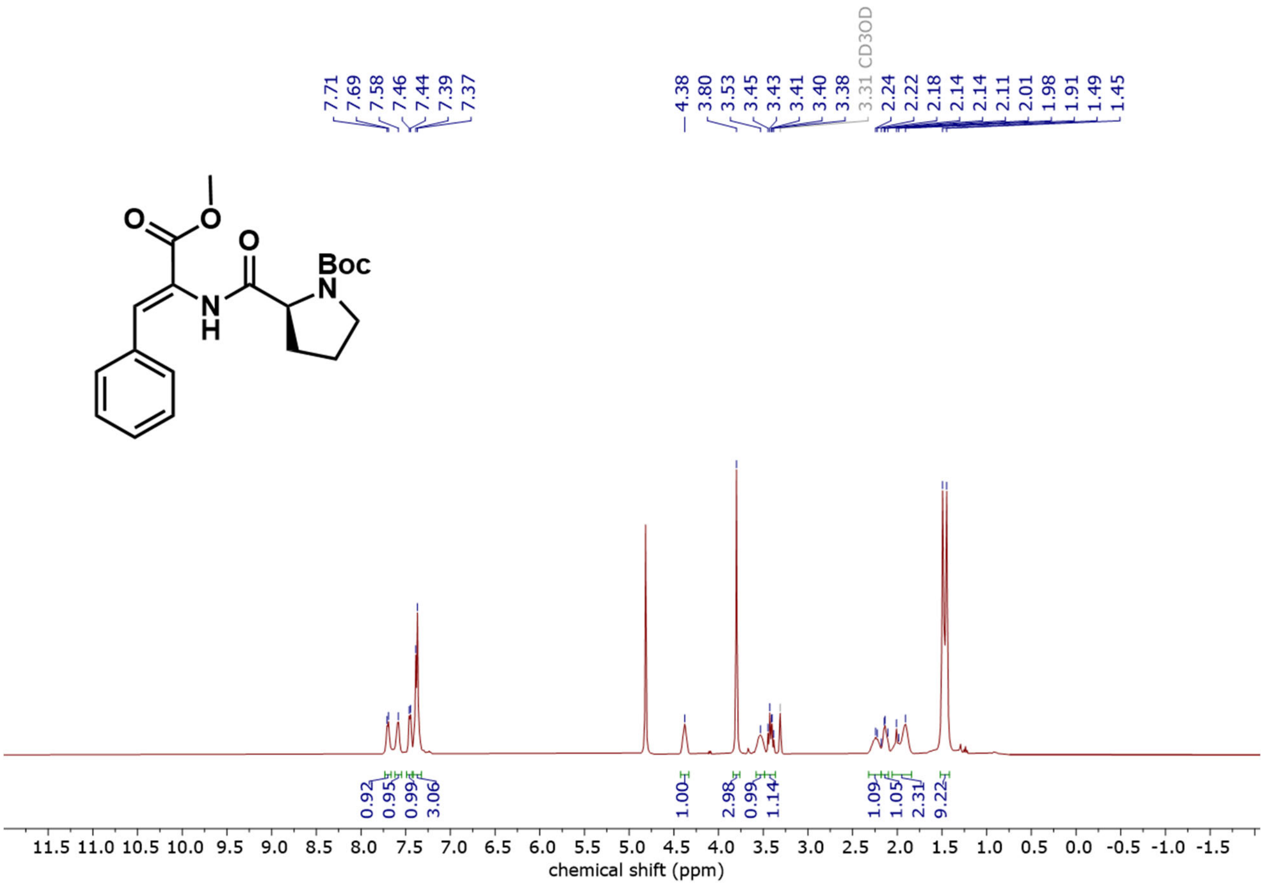

^{13}C NMR of **3w** (101 MHz) in CD_3OD

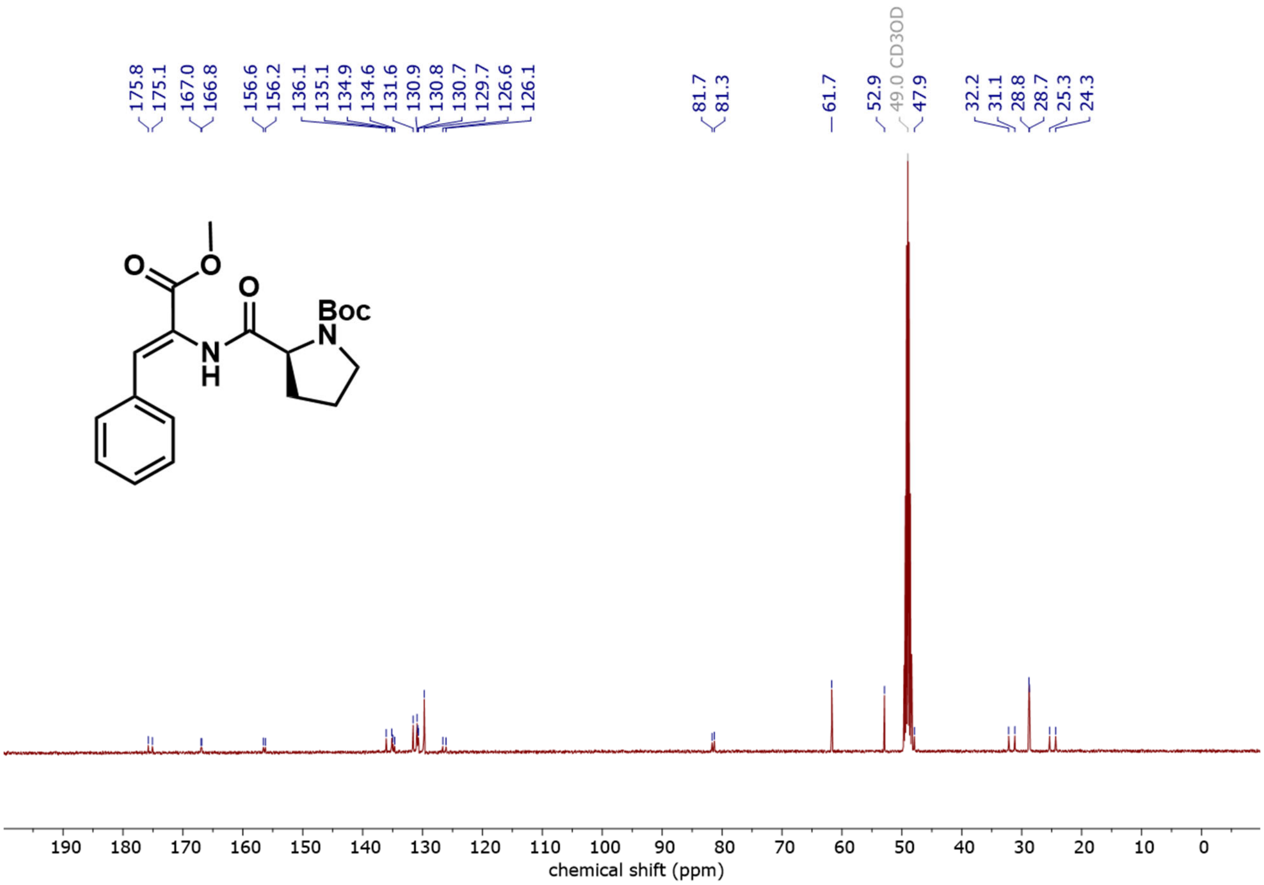

^1H NMR of **3x** (500 MHz) in $CDCl_3$

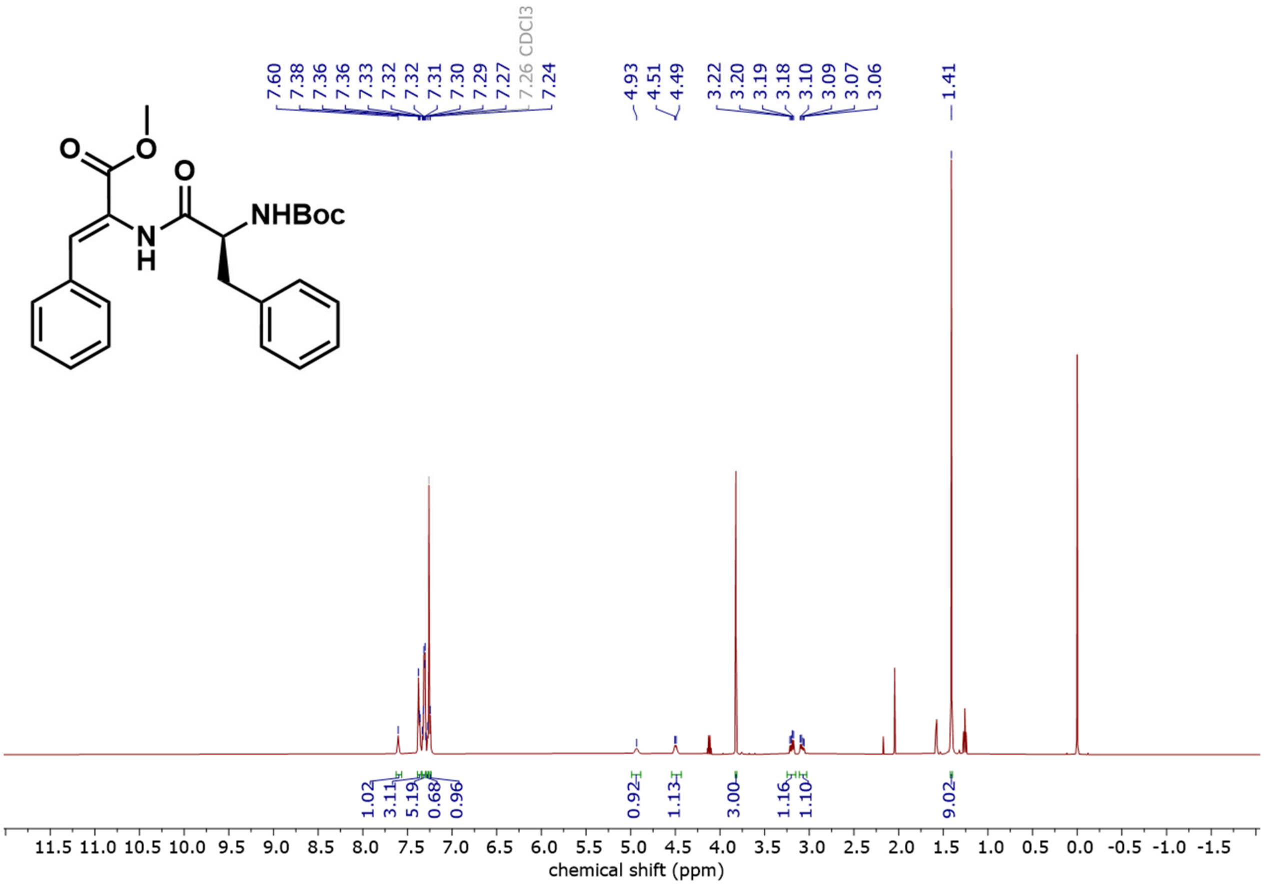

^{13}C NMR of **3x** (126 MHz) in $CDCl_3$

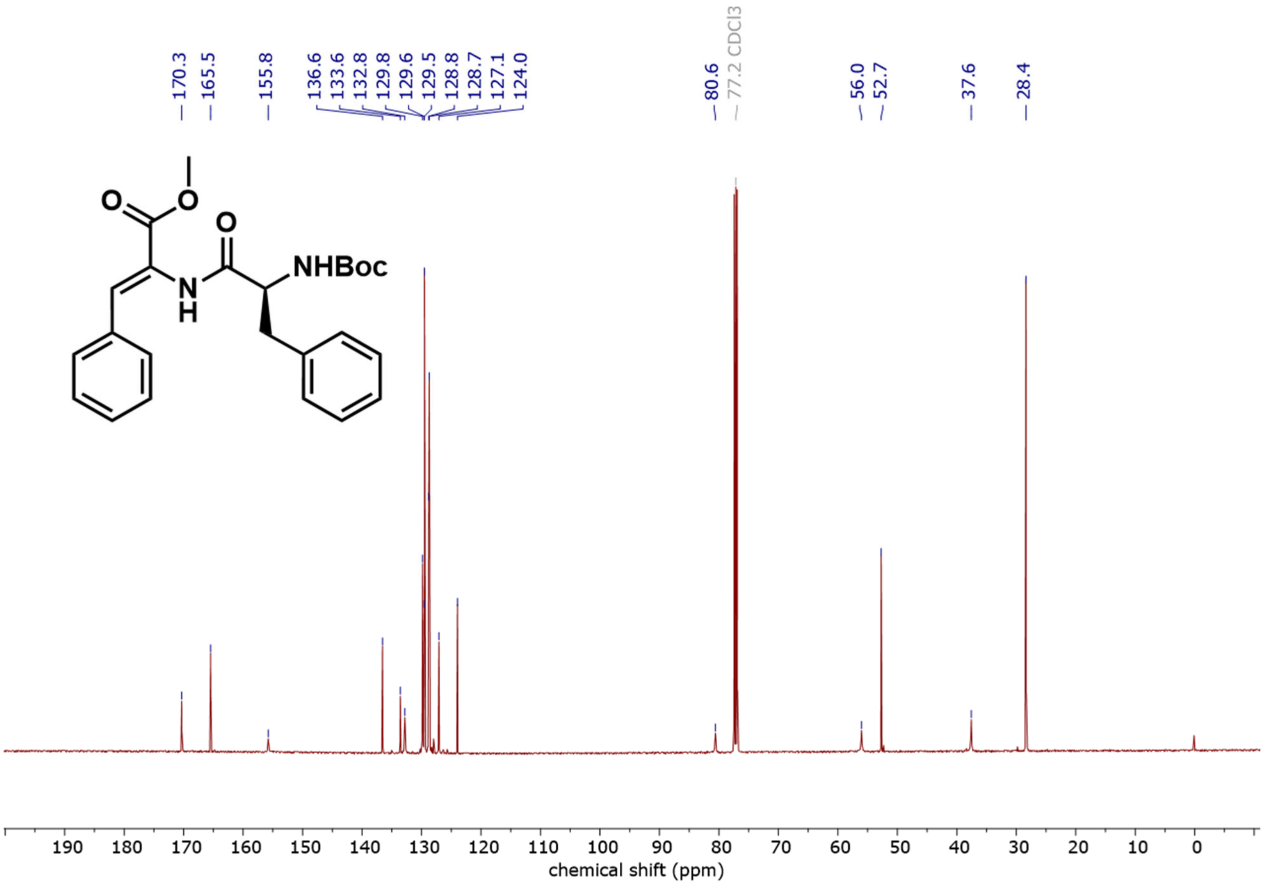

^1H NMR of **3y** (400 MHz) in $CDCl_3$

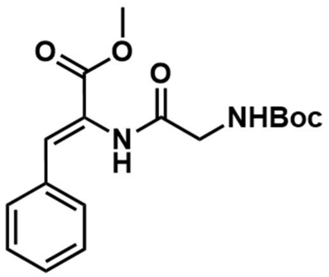

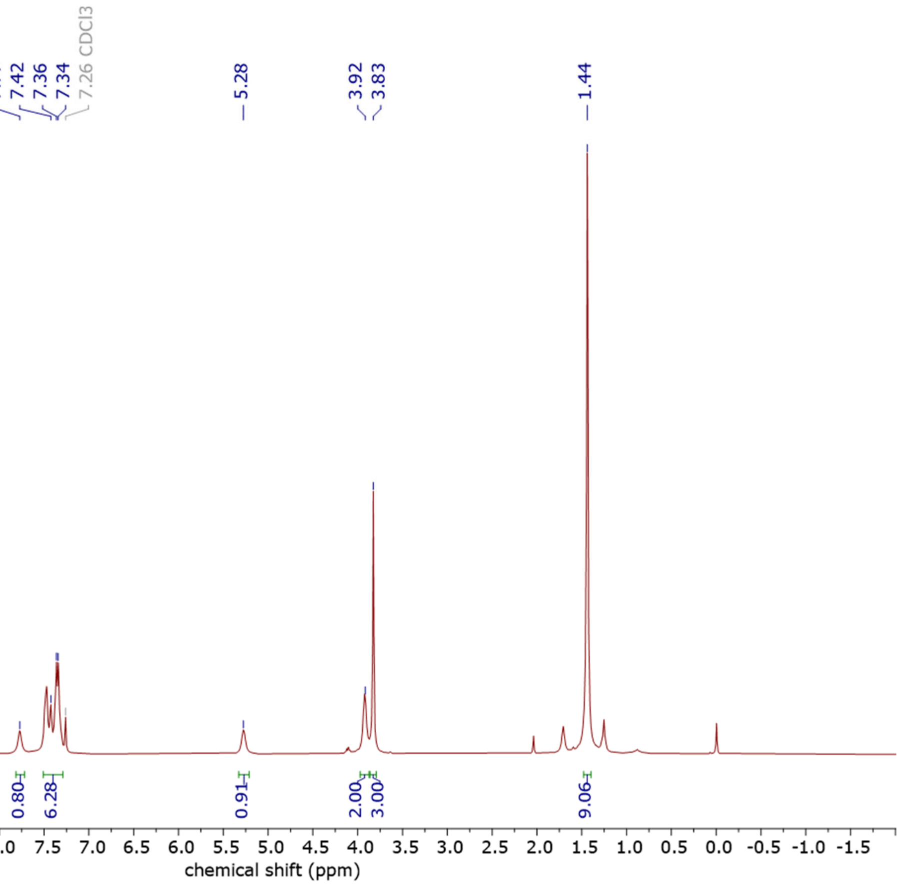

^{13}C NMR of **3y** (151 MHz) in $CDCl_3$

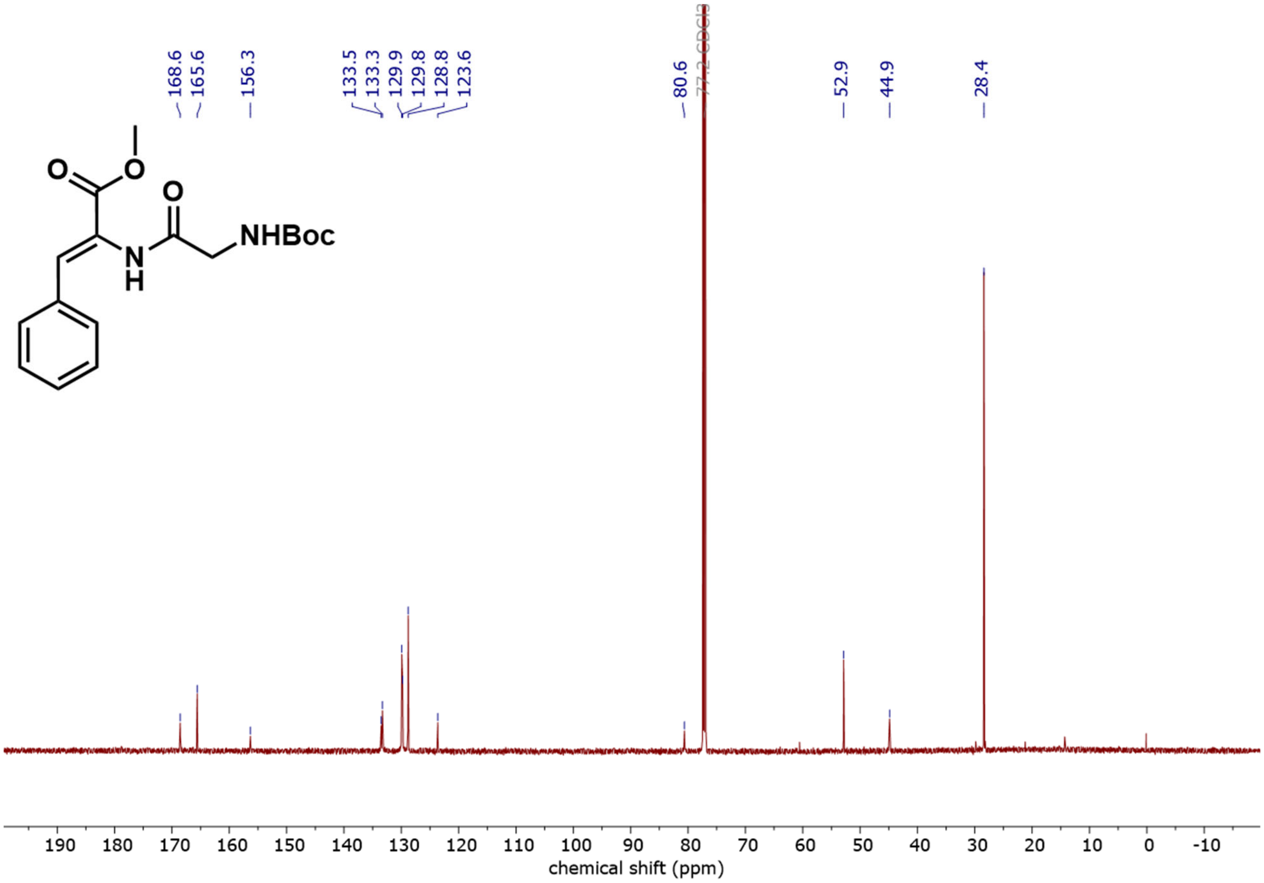

^{1}H NMR of **3z** (400 MHz) in CD_3OD

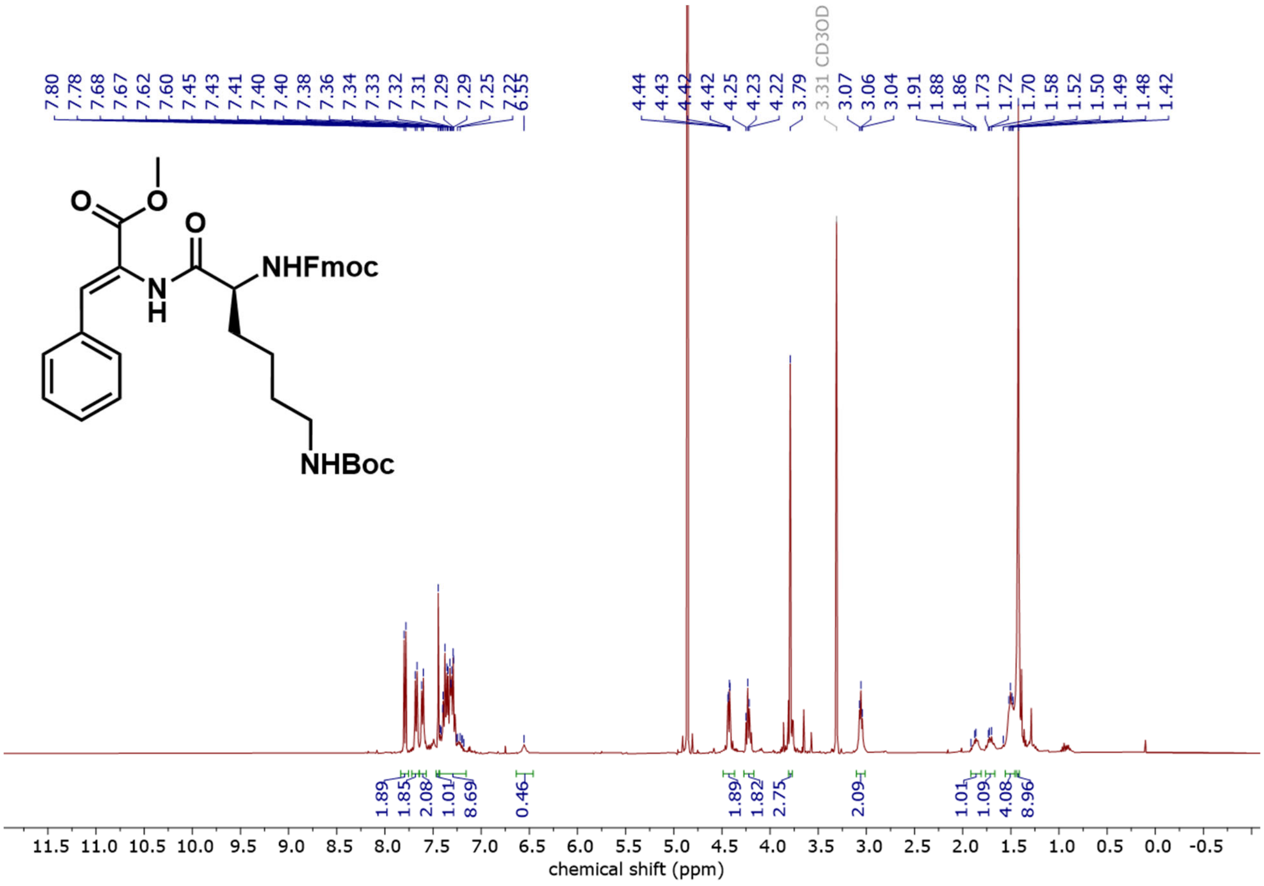

^{13}C NMR of **3z** (126 MHz) in $CDCl_3$

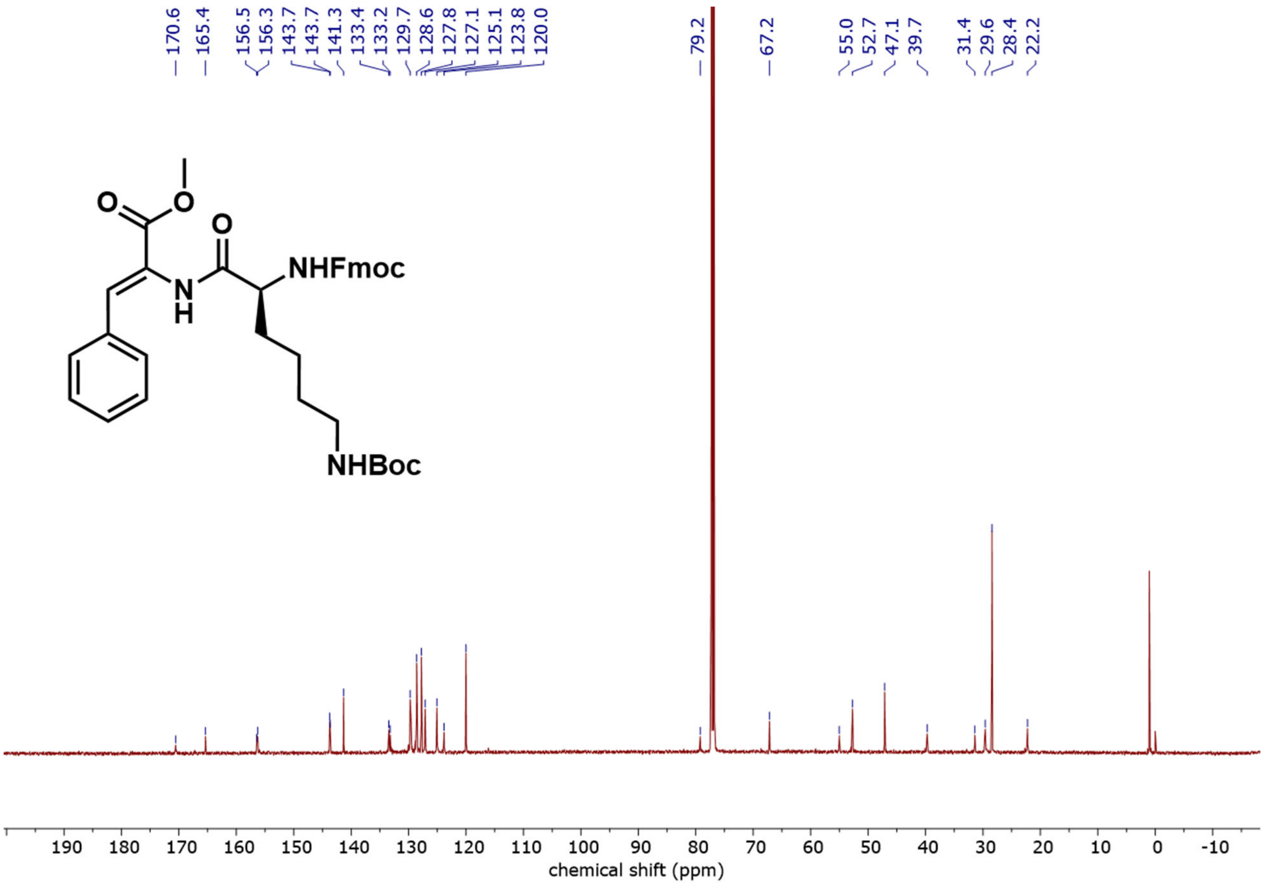

^{1}H NMR of **3aa** (400 MHz) in CD_3OD

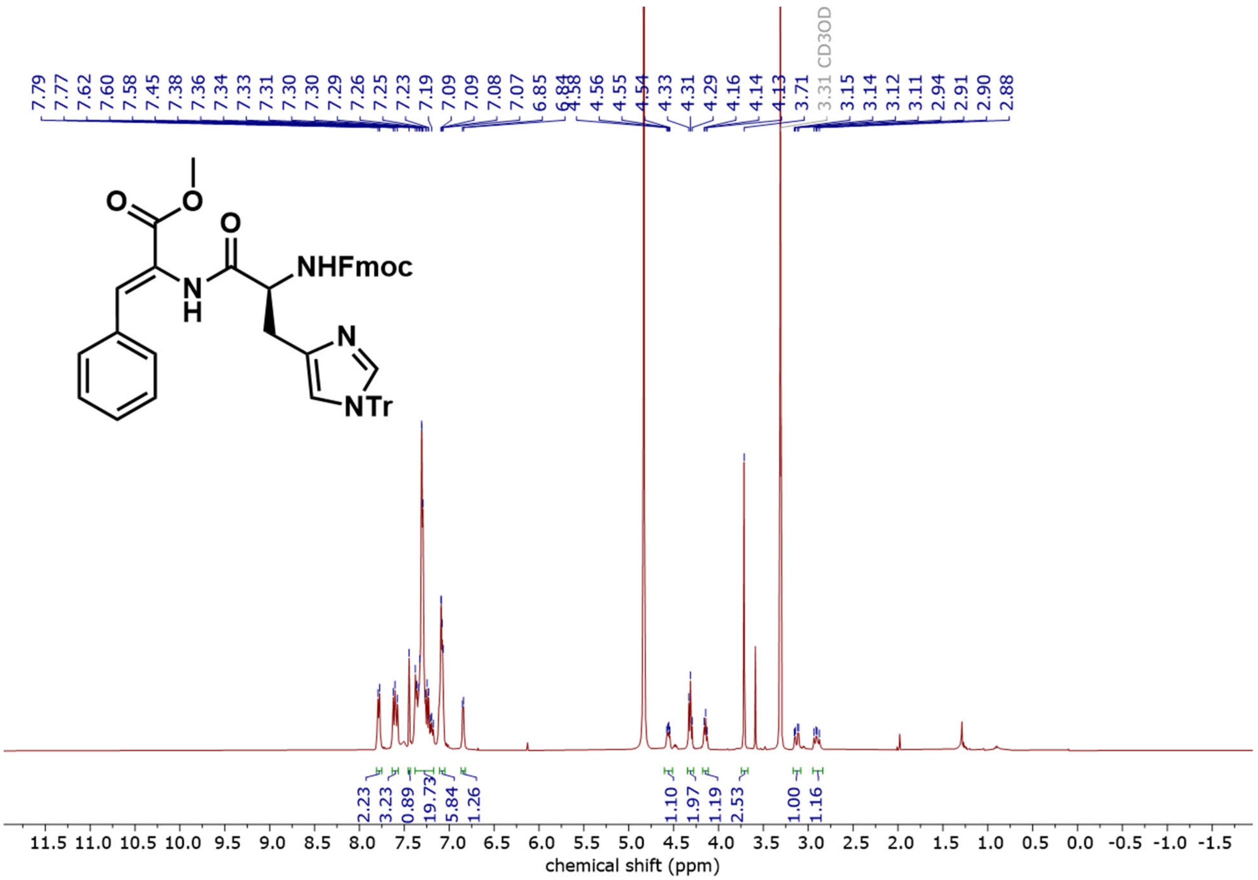

^{13}C NMR of **3aa** (126 MHz) in CD_3OD

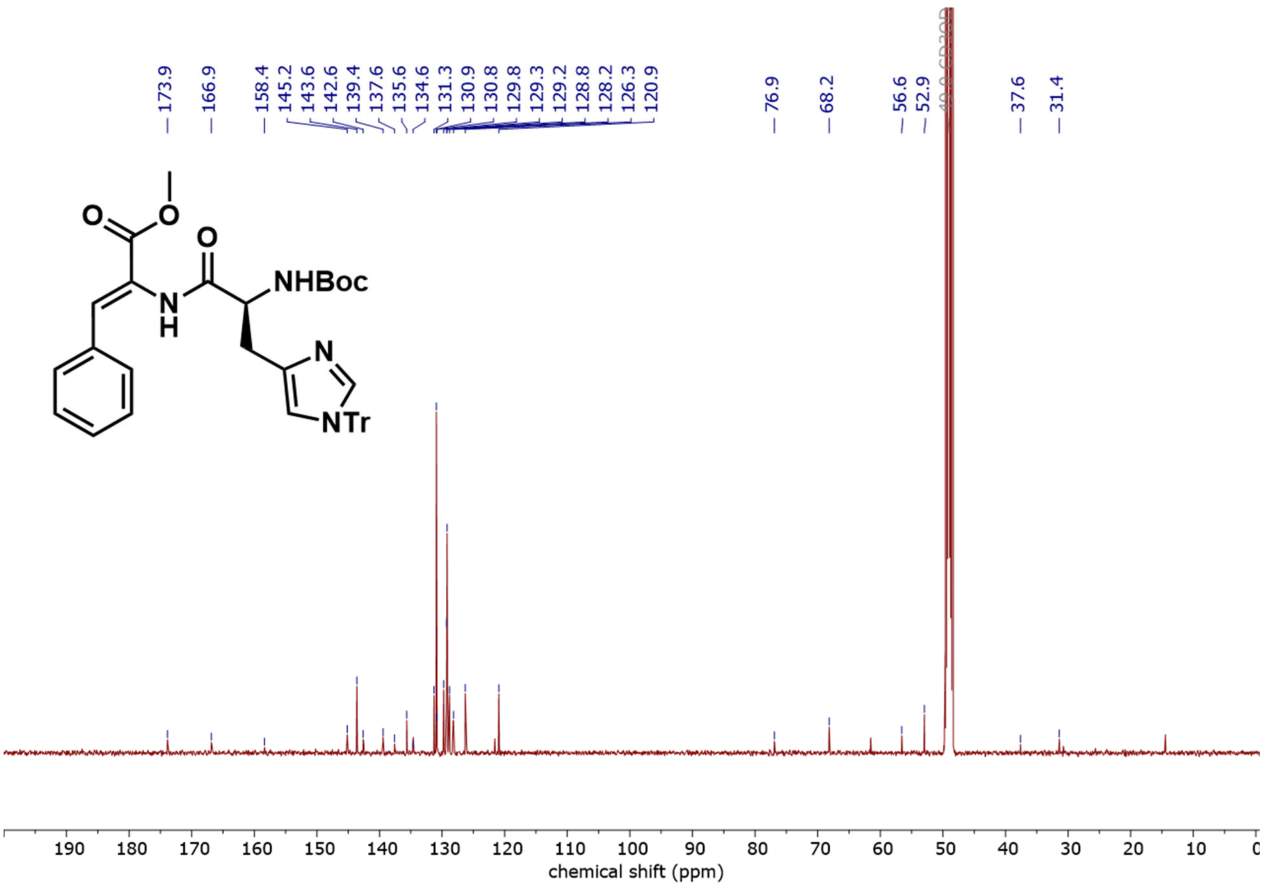

^{1}H NMR of **3ab** (500 MHz) in $CDCl_3$

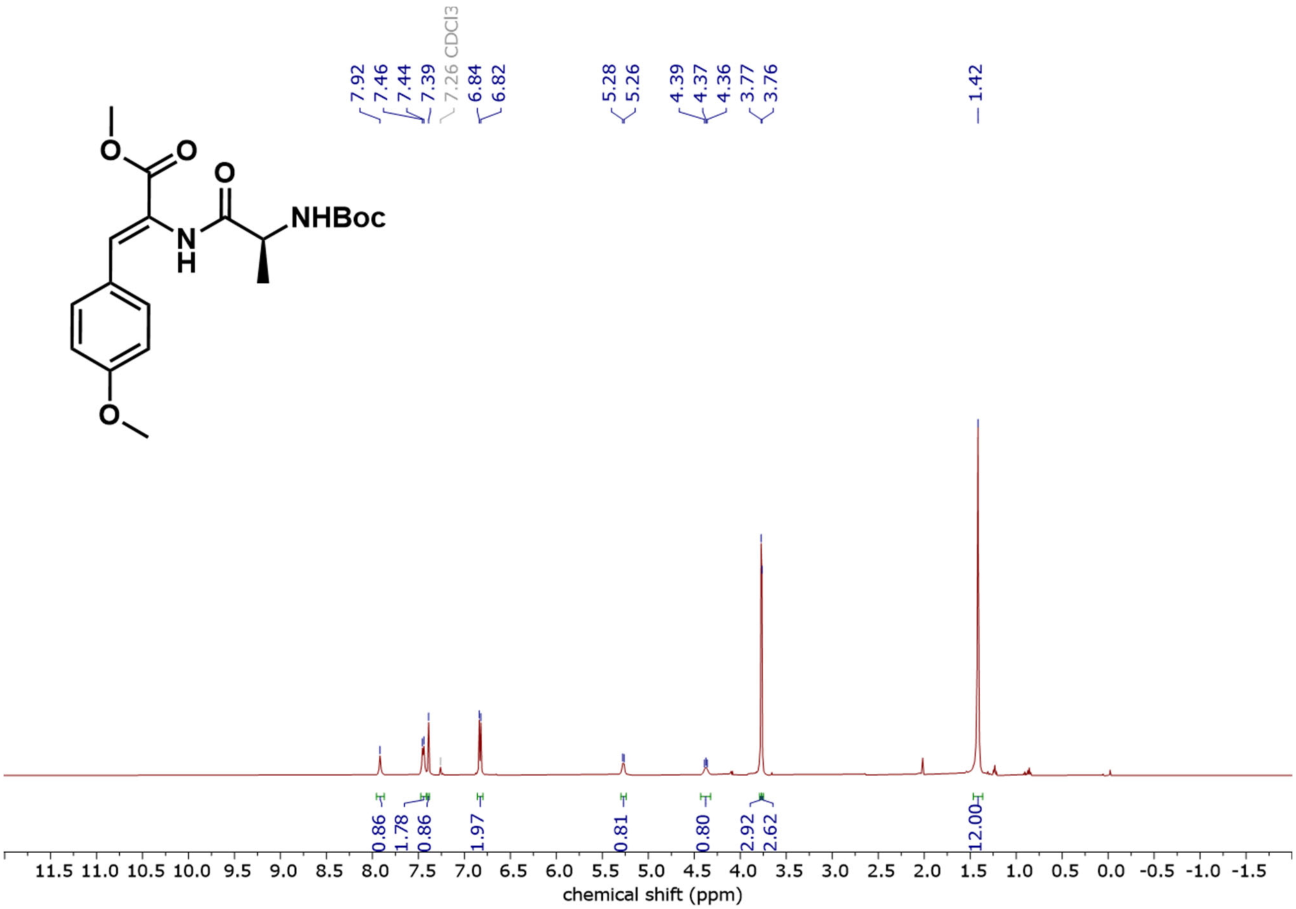

^{13}C NMR of **3ab** (126 MHz) in $CDCl_3$

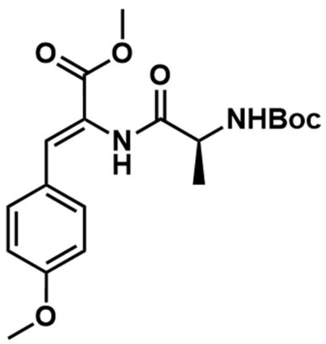

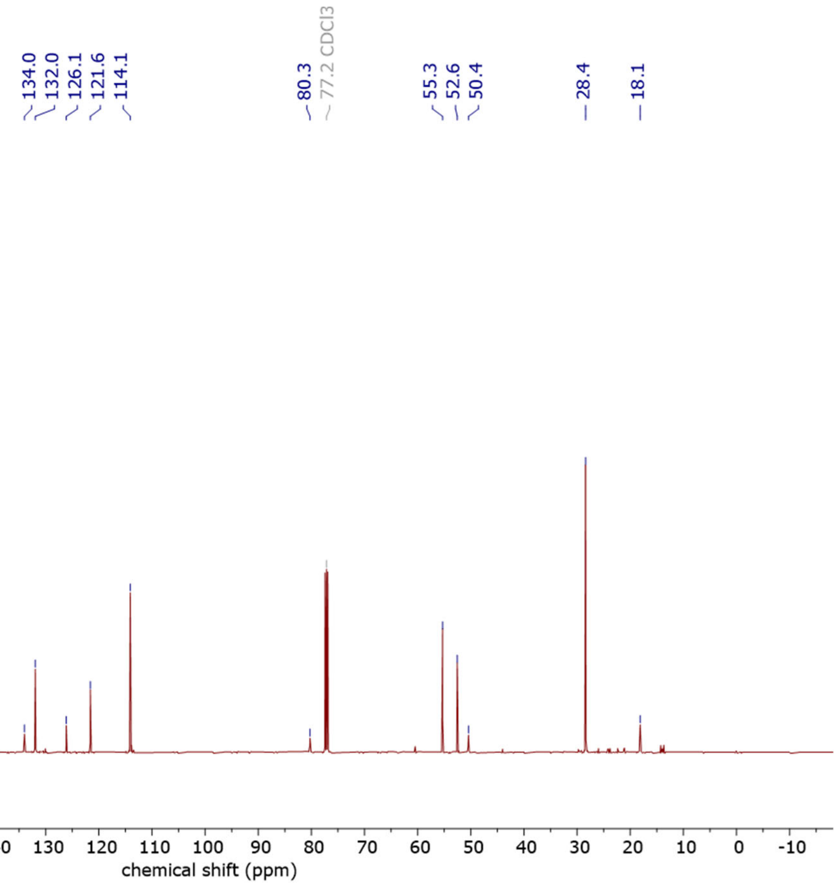

^{1}H NMR of **3ac** (400 MHz) in $CDCl_3$

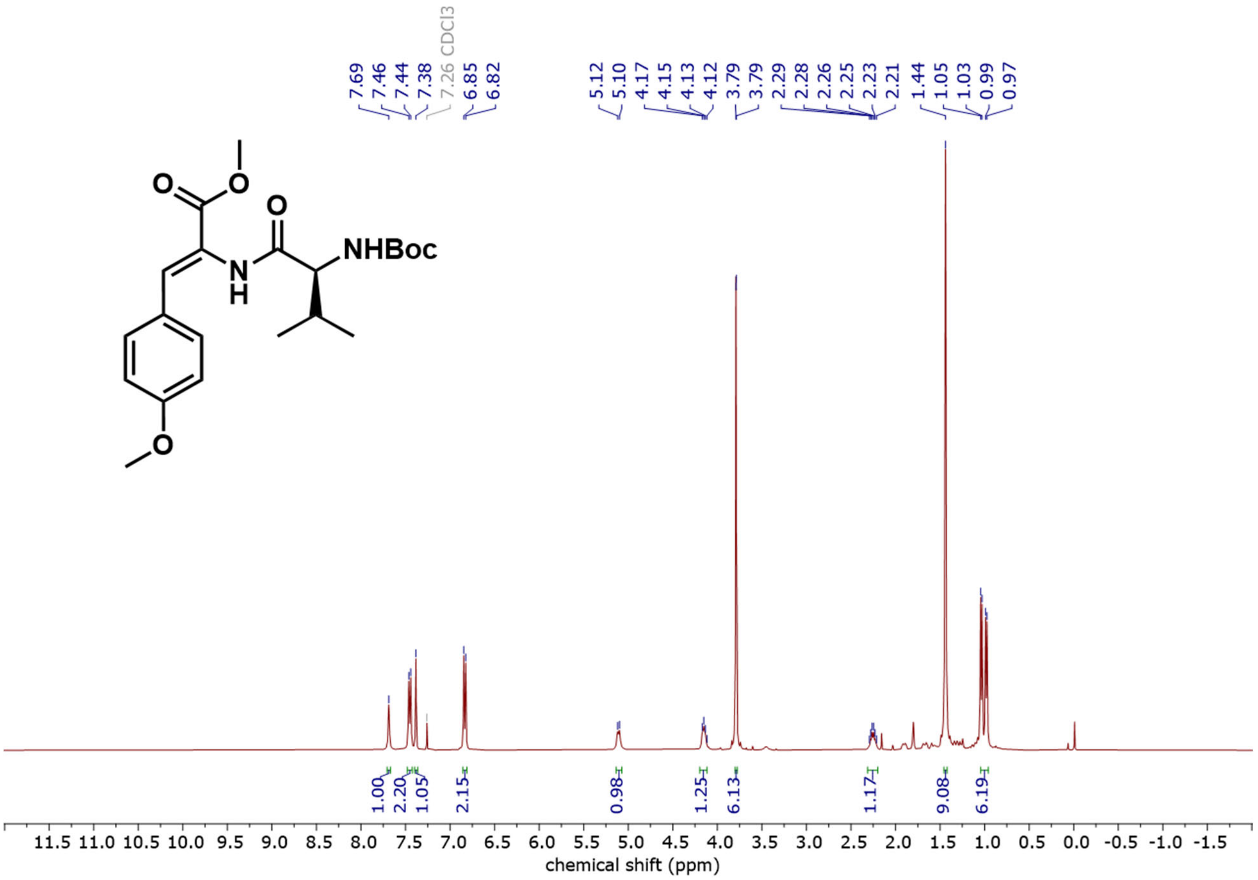

^{13}C NMR of **3ac** (101 MHz) in $CDCl_3$

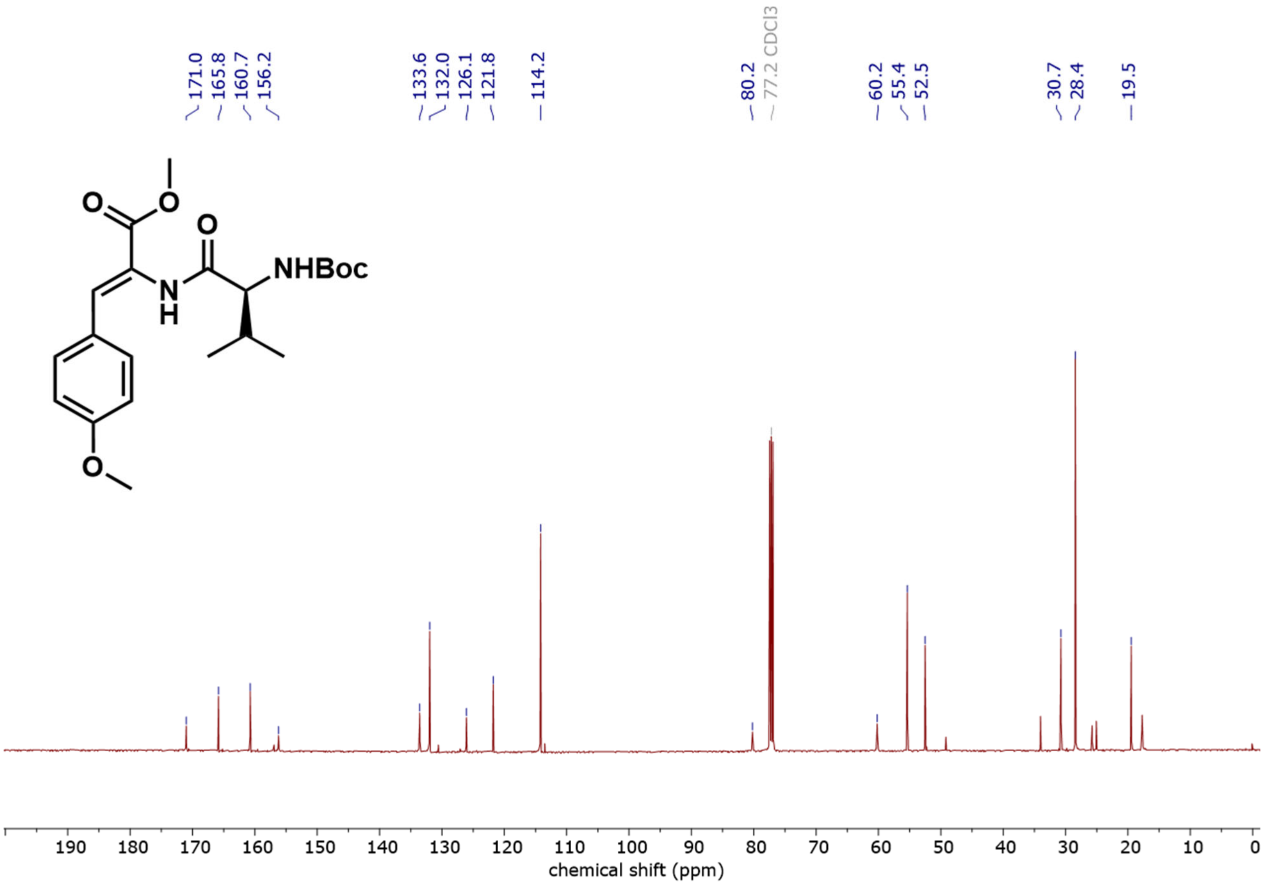

^{1}H NMR of **3ad** (400 MHz) in $CDCl_3$

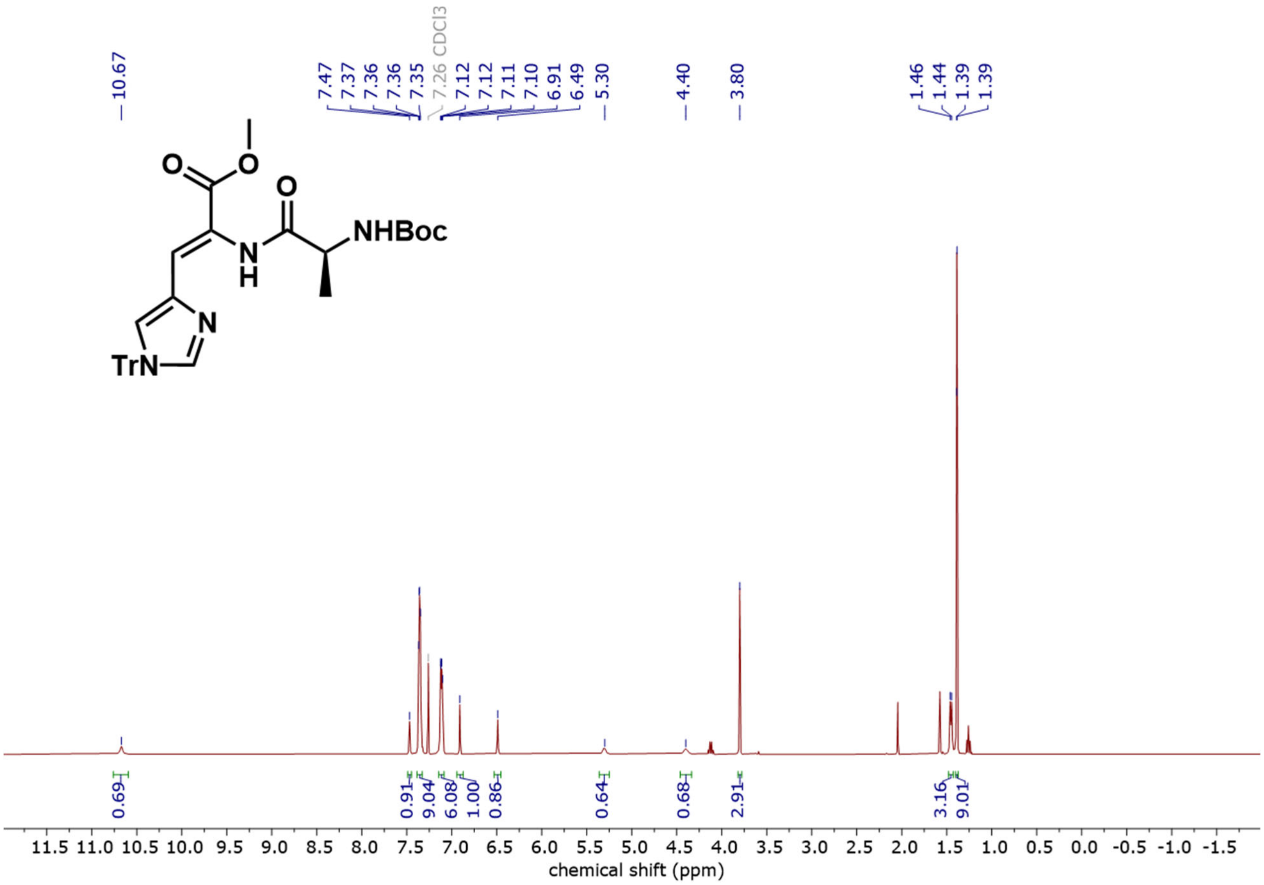

^{13}C NMR of **3ad** (126 MHz) in $CDCl_3$

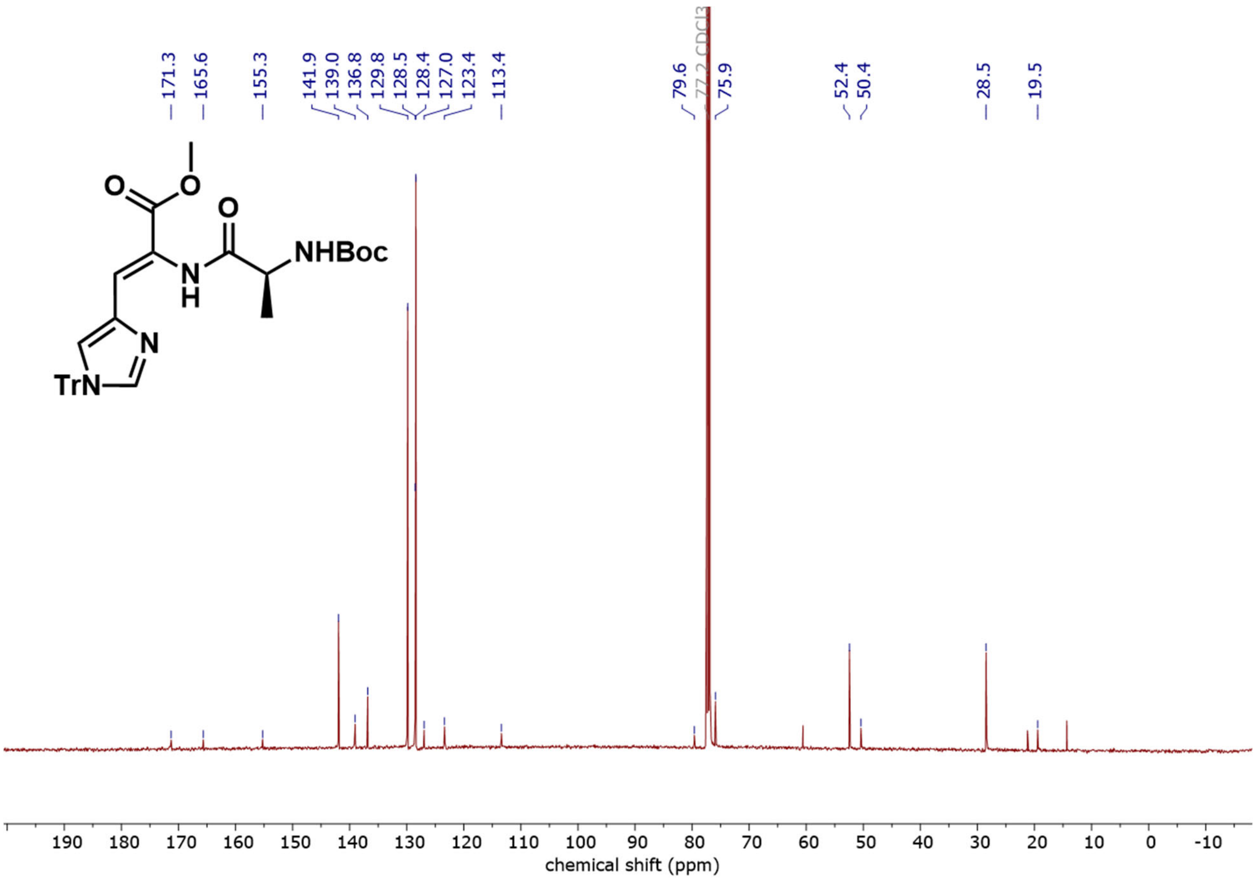

^1H NMR of **3ae** (400 MHz) in $CDCl_3$

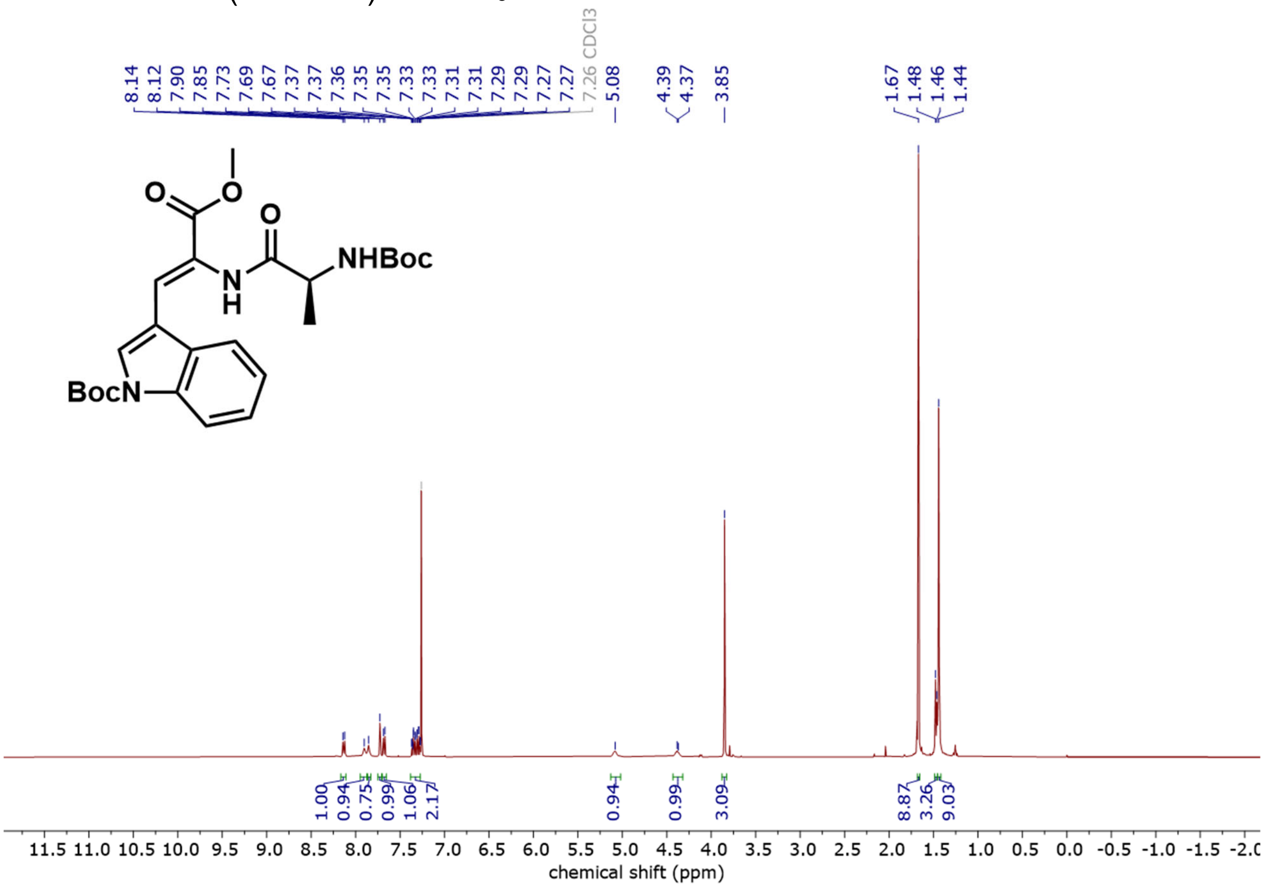

^{13}C NMR of **3ae** (101 MHz) in $CDCl_3$

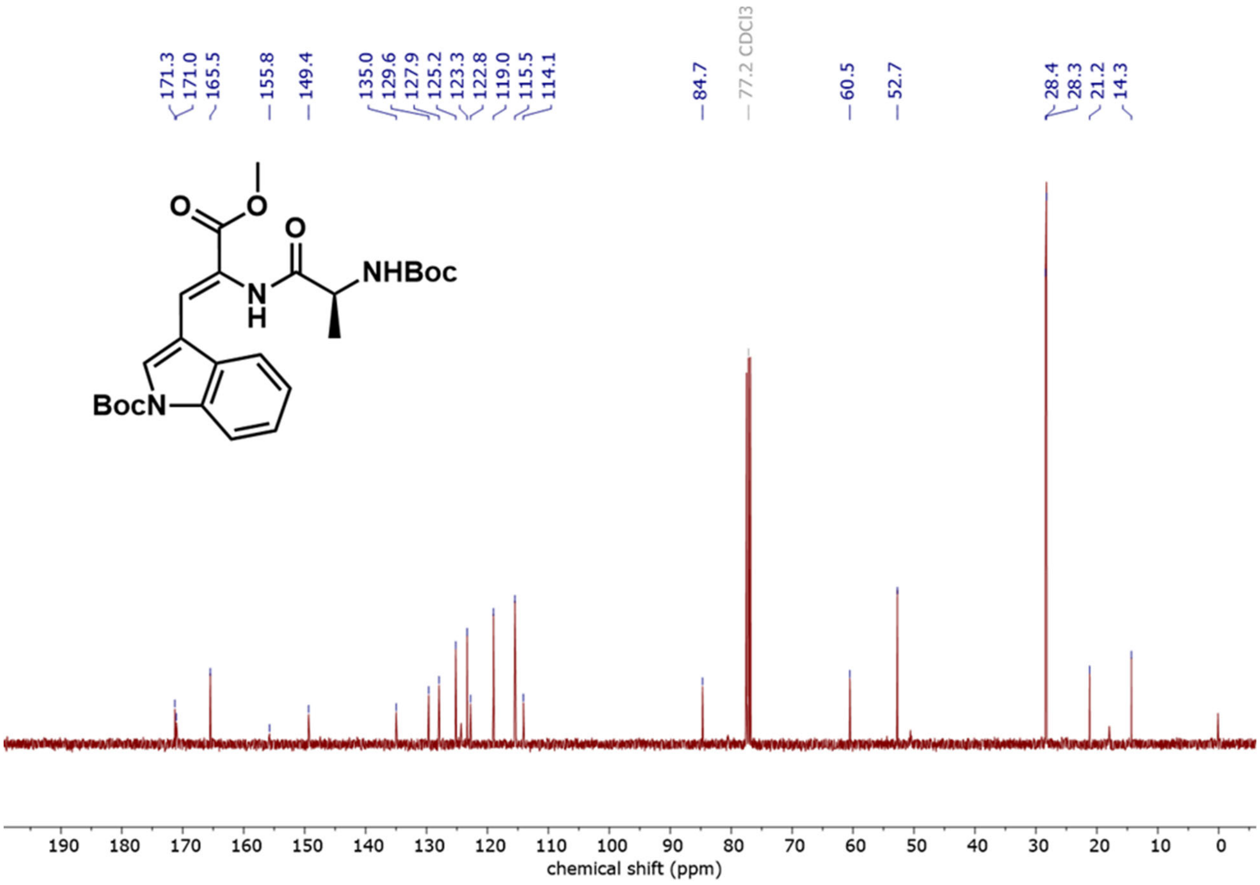

^1H NMR of **4** (400 MHz) in DMSO-d_6

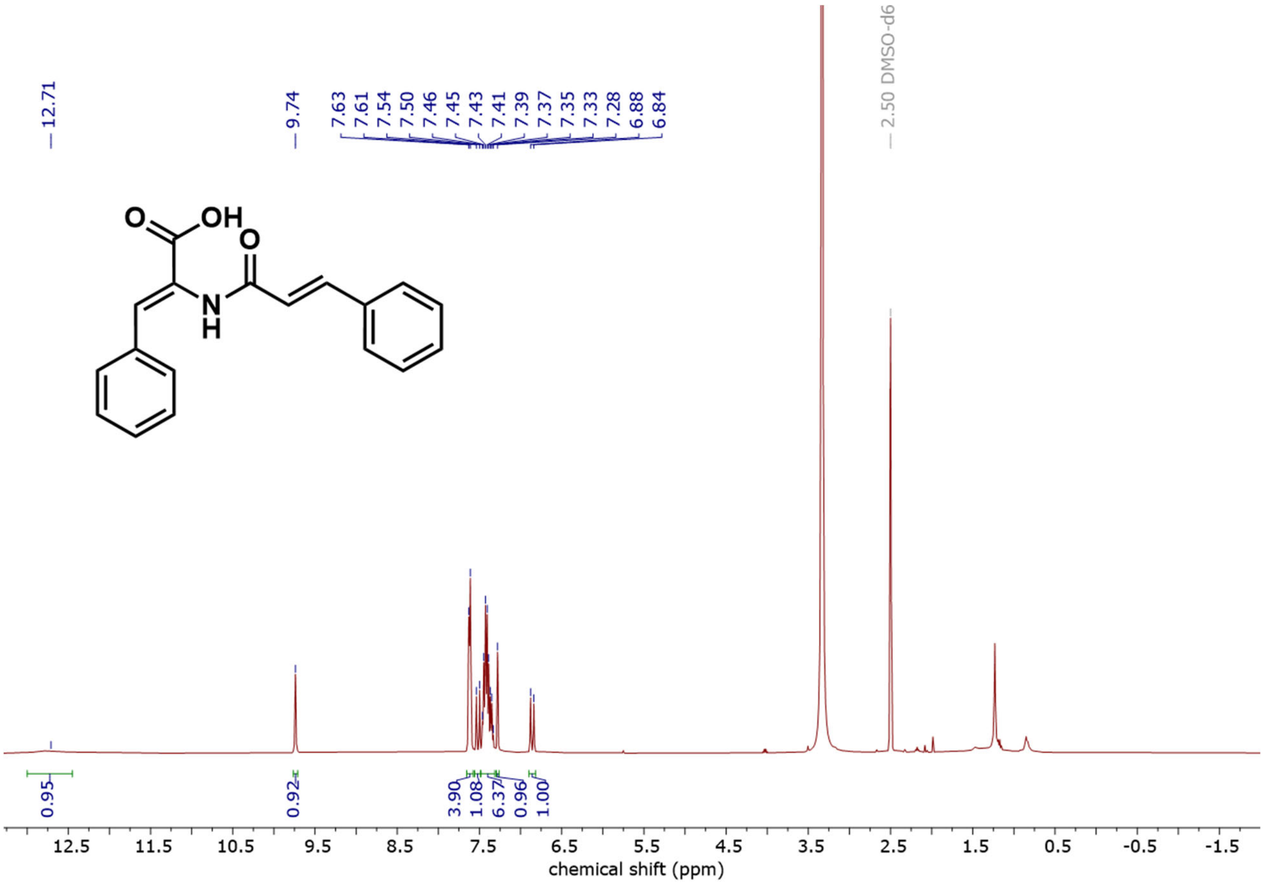

^{13}C NMR of **4** (101 MHz) in DMSO-d$_6$

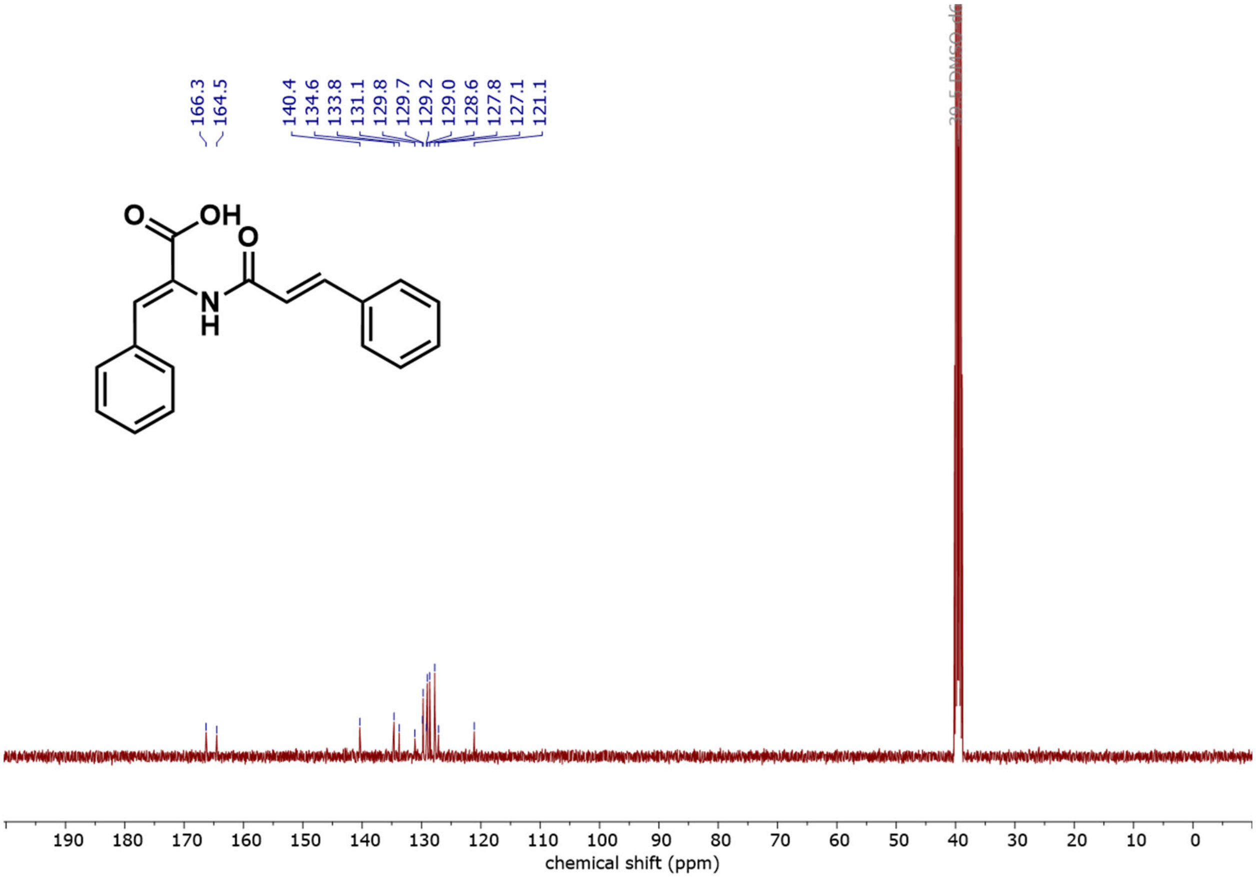

^1H NMR of Scutianene M (400 MHz) in DMSO-d_6

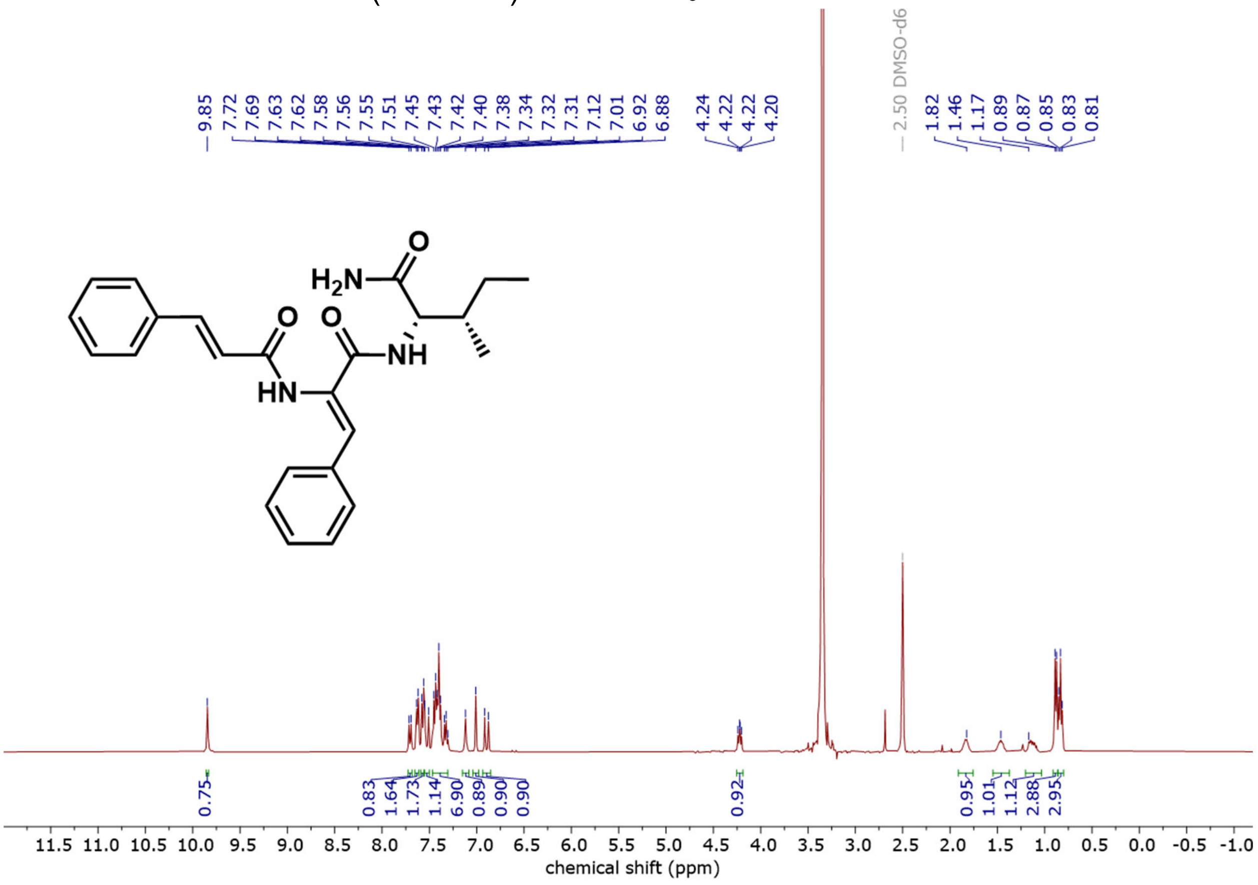

^{13}C NMR of Scutianene M (126 MHz) in DMSO-d_6

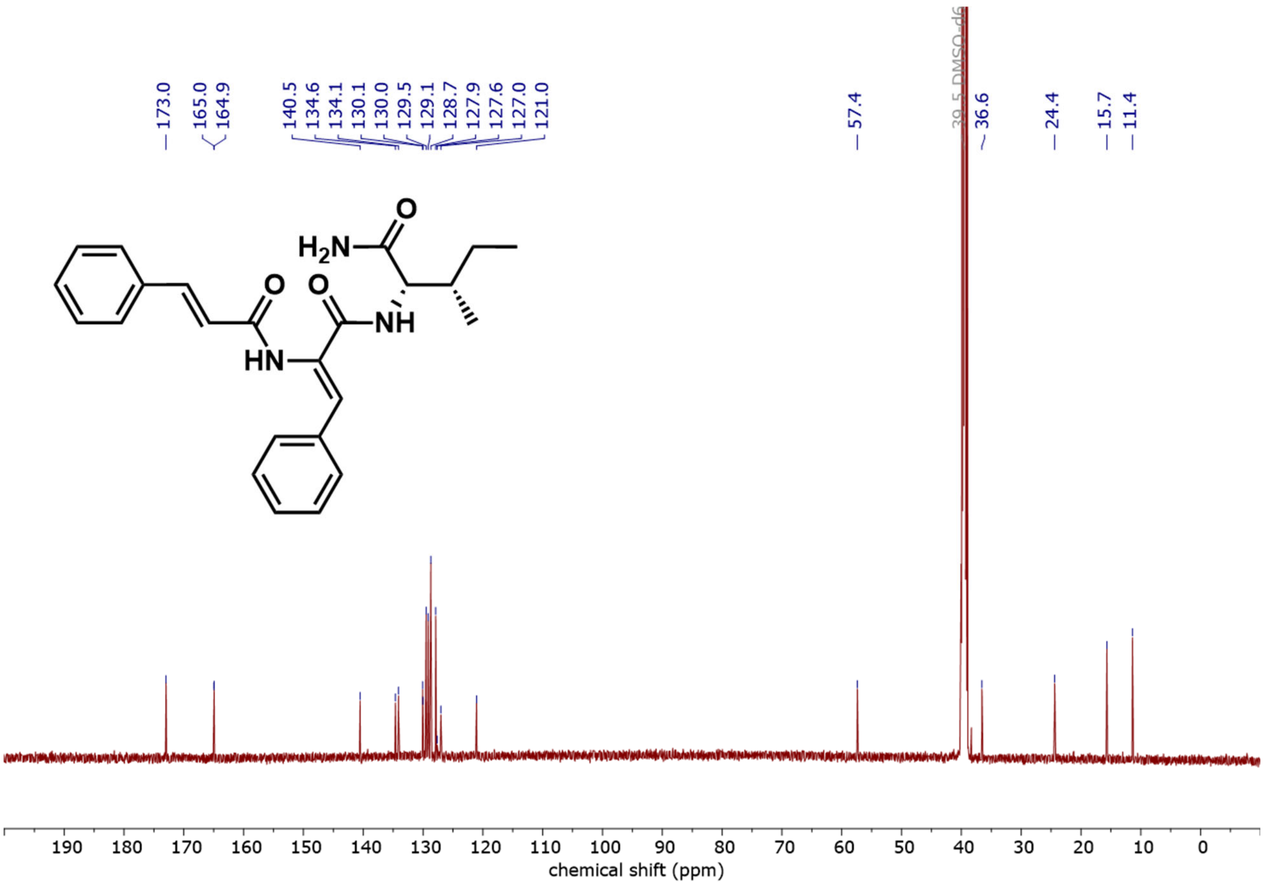

^{1}H NMR of **5** (600 MHz) in DMSO-d$_6$

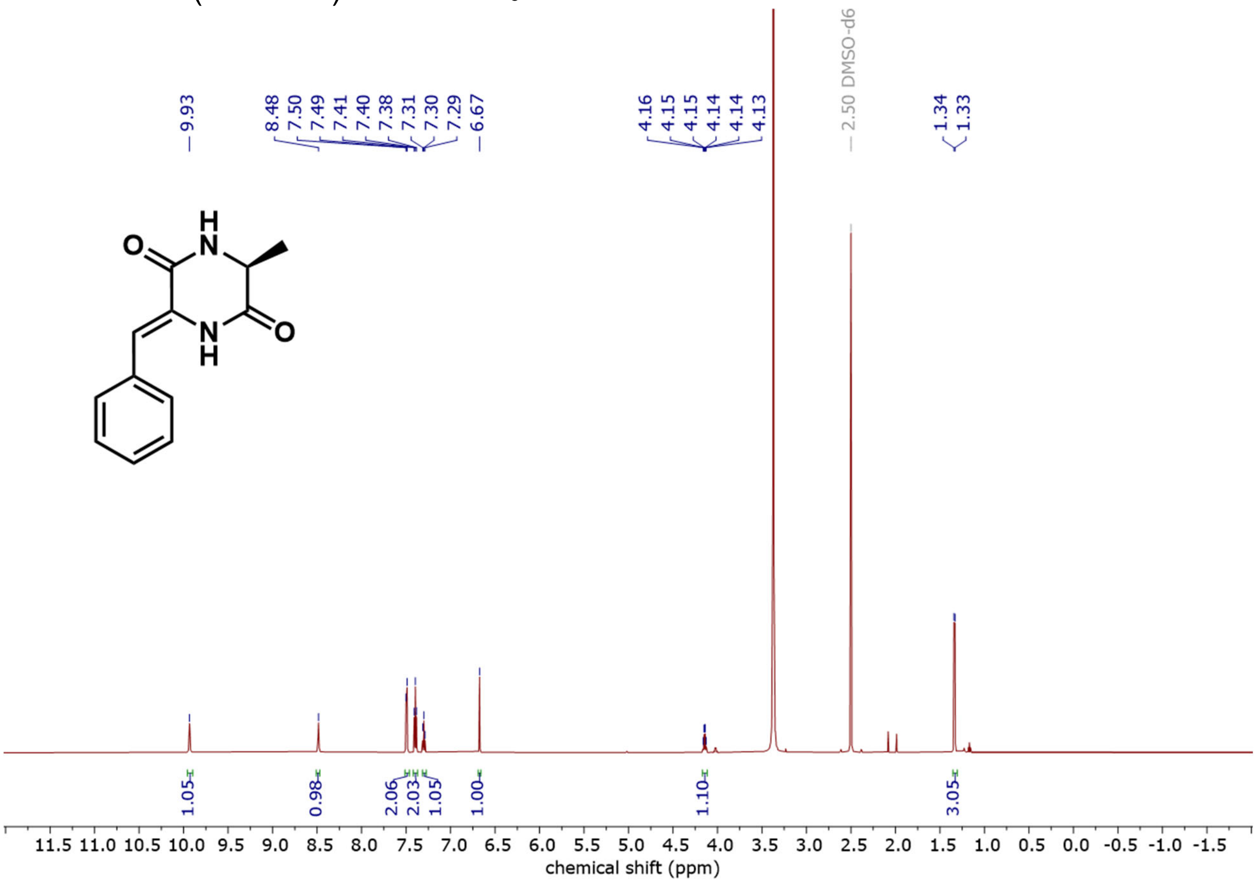

^{13}C NMR of **5** (151 MHz) in DMSO-d$_6$

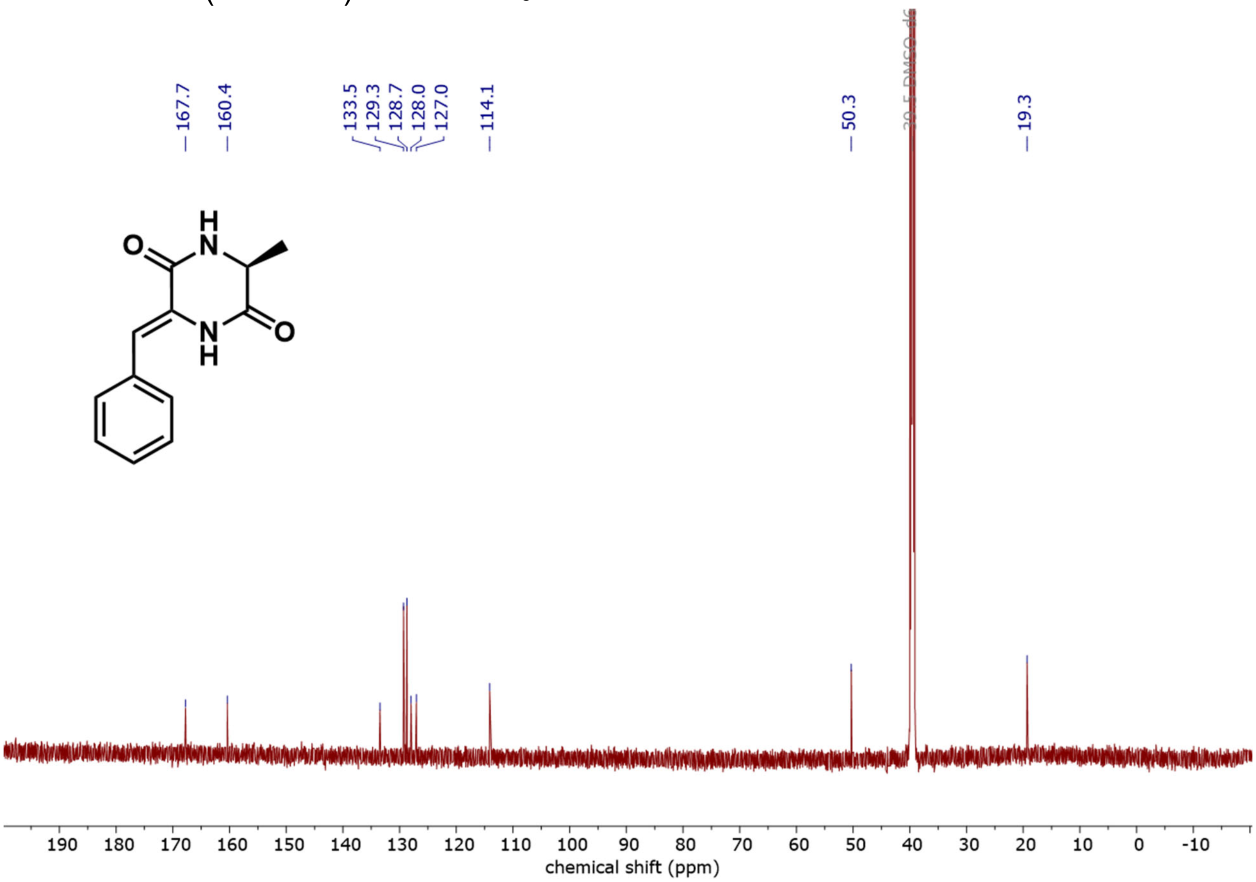

^{1}H NMR of **S5** (400 MHz) in DMSO-d$_6$

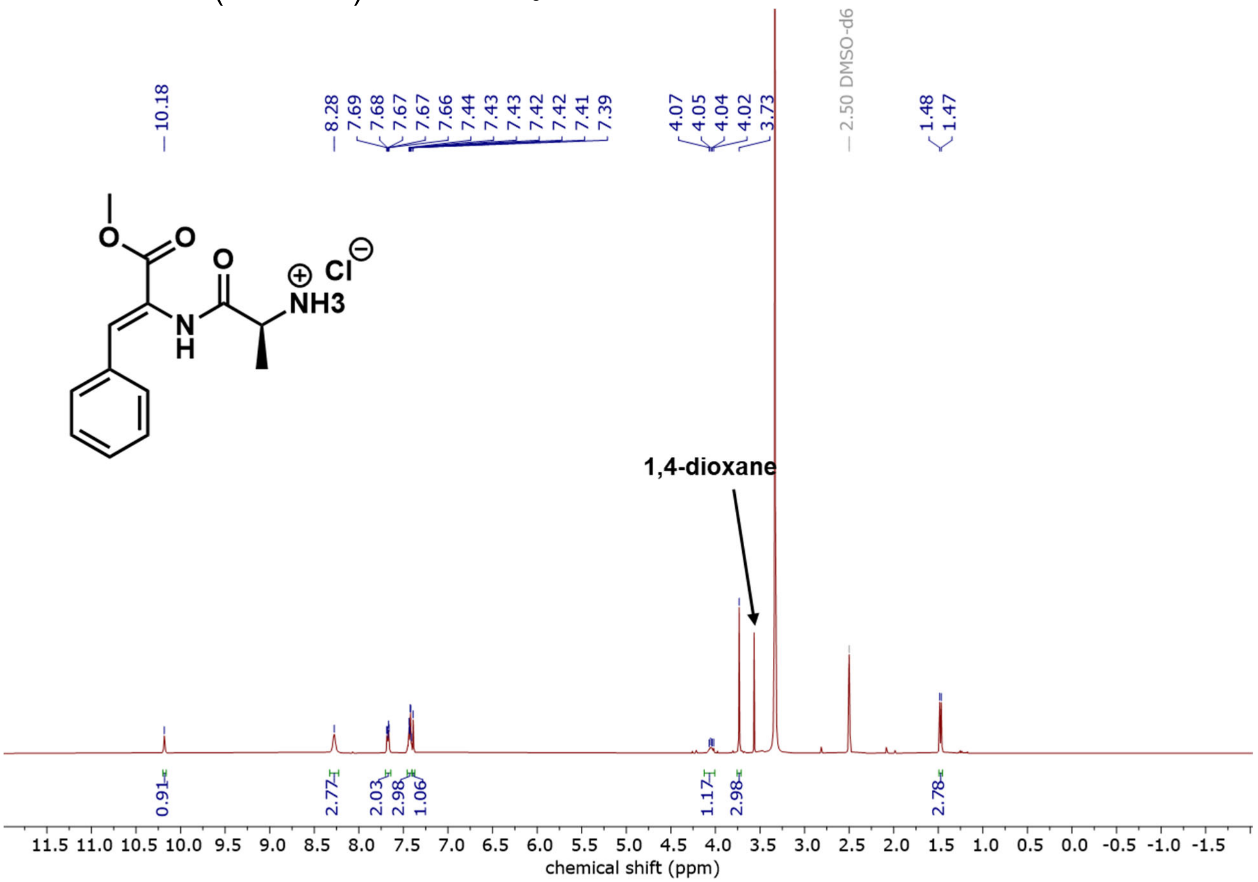

^{13}C NMR of **S5** (126 MHz) in DMSO-d$_6$

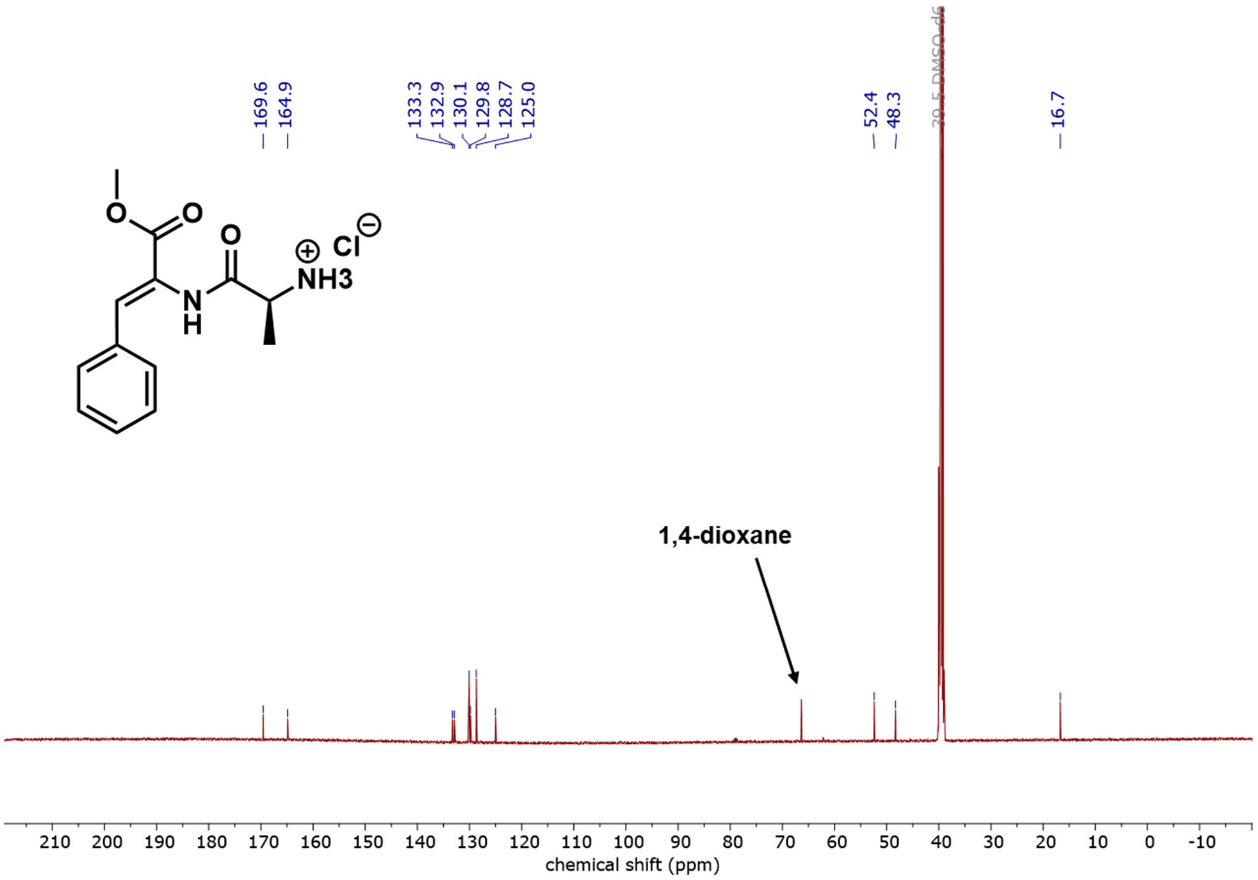

^{1}H NMR of **S6** (400 MHz) in $CDCl_3$

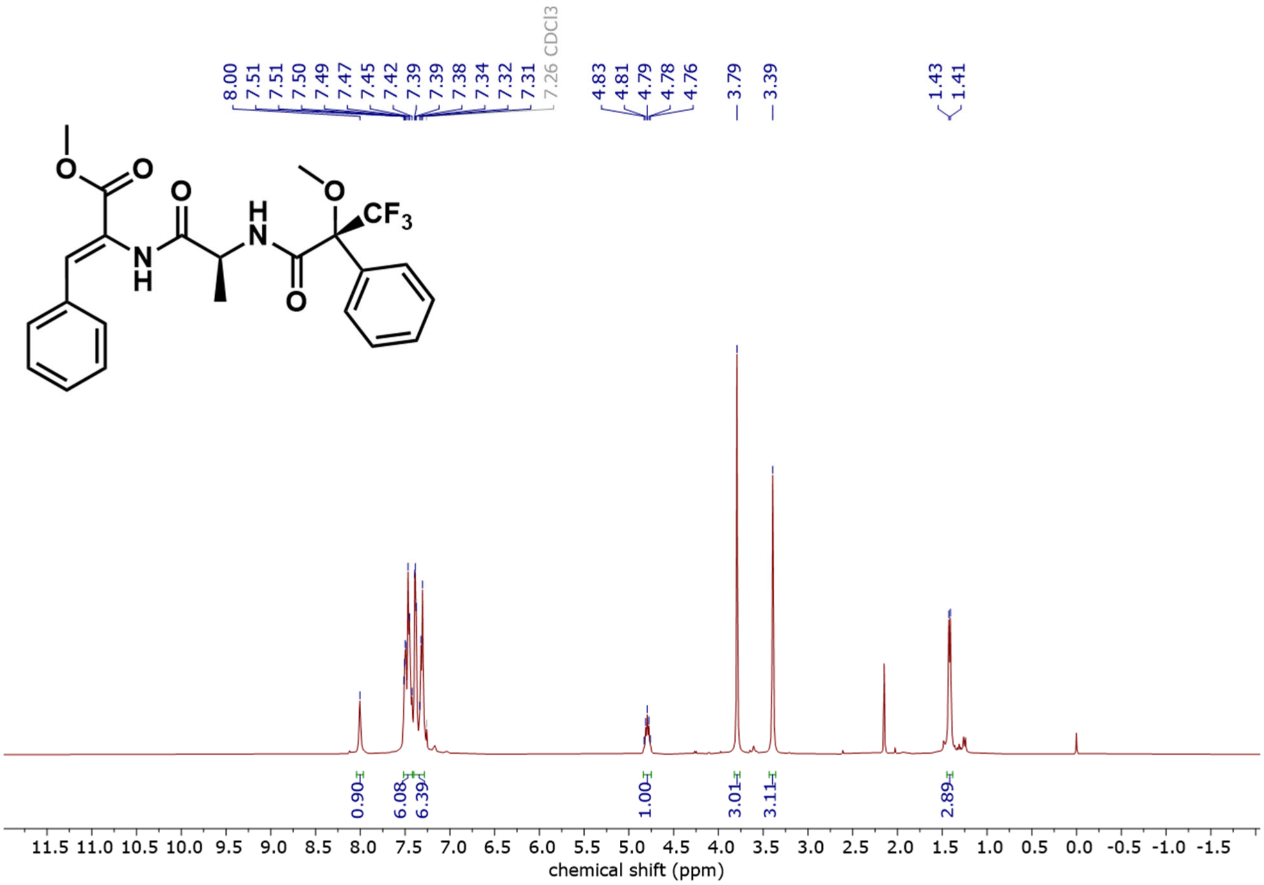

^{13}C NMR of **S6** (126 MHz) in $CDCl_3$

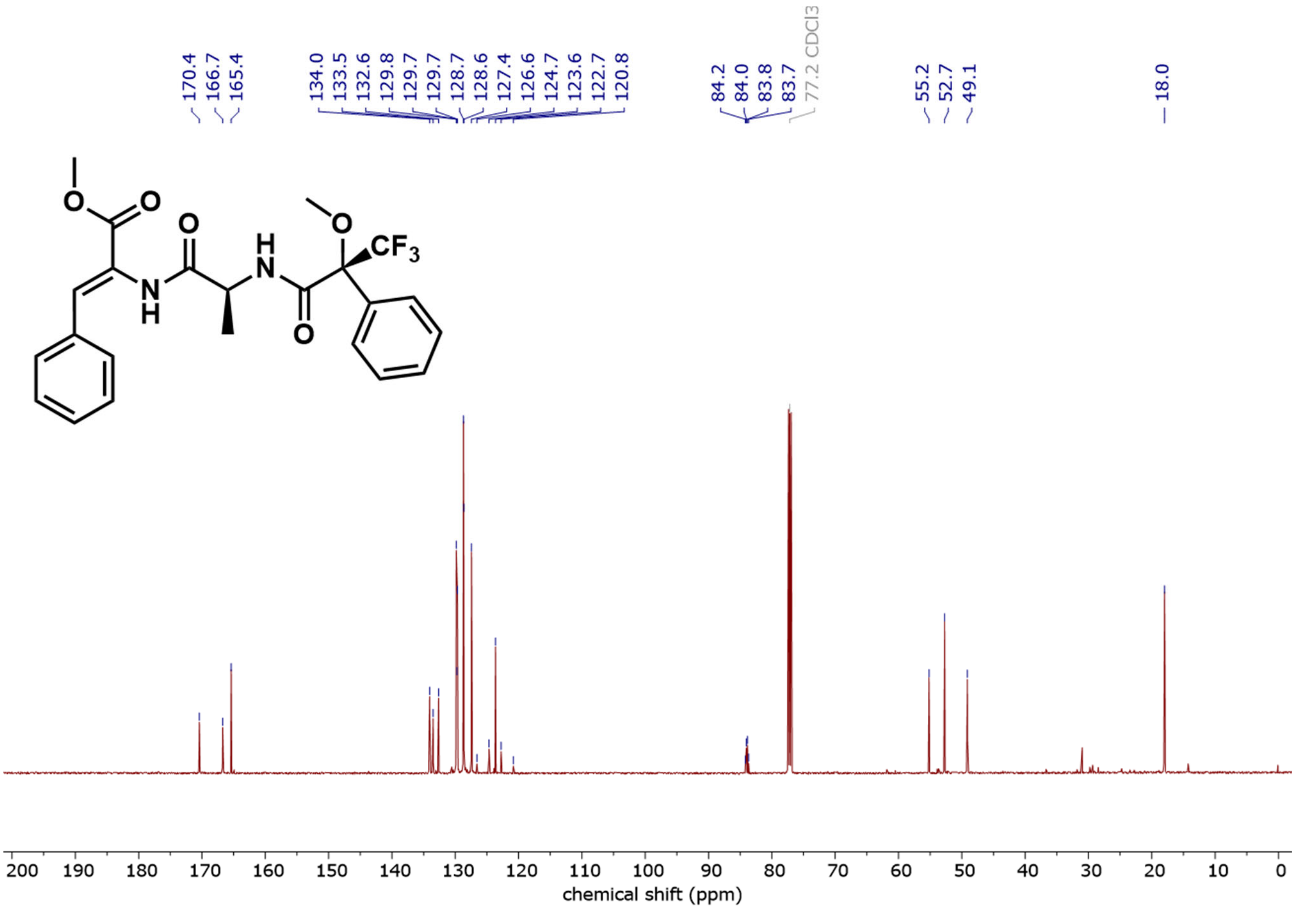

^{19}F NMR of **S6** (376 MHz) in $CDCl_3$

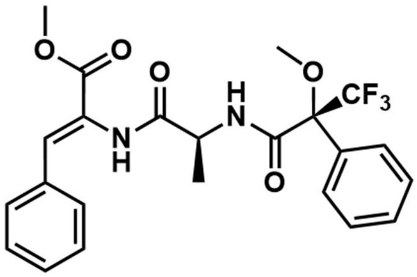

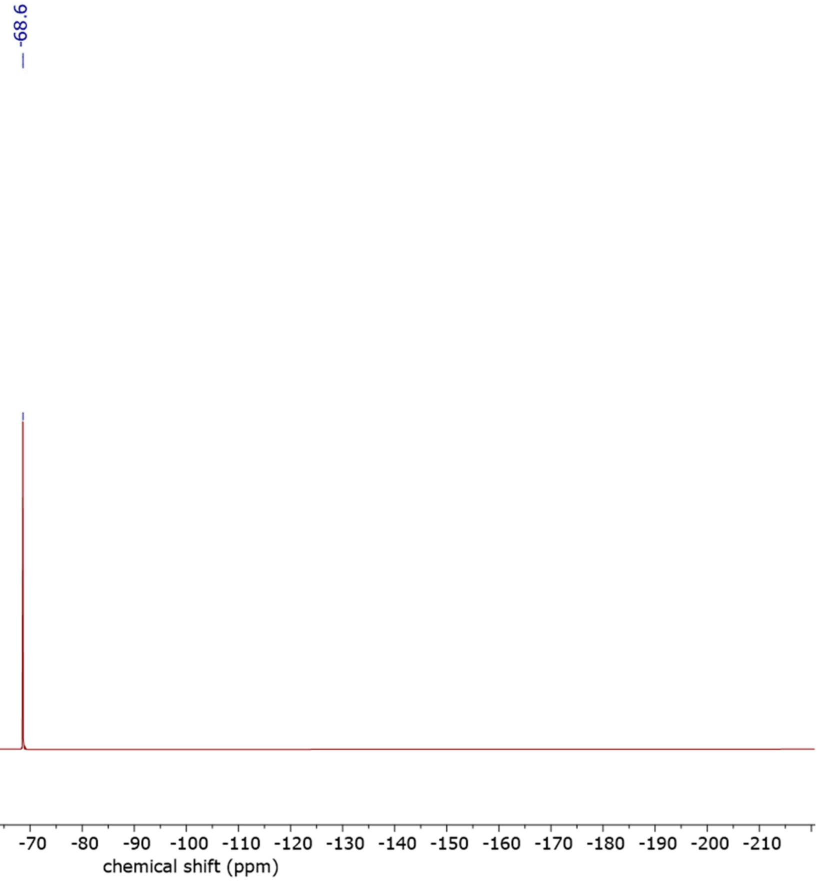

10. References

1. Varongchayakul, C.; Danheiser, R. L., One-pot Synthesis of Alkynes from Esters via a Tandem Reduction-Ohira-Bestmann Reaction. *Org. Synth.* **2024,** *101*.
2. Dolensky, B.; Kirk, K. L., New approaches to side-chain fluorinated bioimidazoles: 4-alkynylimidazoles as substrates for fluorination. *J. Fluorine Chem.* **2003,** *124*, 105-110.
3. Trotta, A. H., Total synthesis of oridamycins A and B. *Org. Lett.* **2015,** *17*, 3358-3361.
4. He, W.; Li, C.; Zhang, L., An efficient [2+ 2+ 1] synthesis of 2, 5-disubstituted oxazoles via gold-catalyzed intermolecular alkyne oxidation. *J. Am. Chem. Soc.* **2011,** *133*, 8482-8485.
5. Wang, W.; Zhou, S.; Li, L.; He, Y.; Dong, X.; Gao, L.; Wang, Q.; Song, Z., 3-Silaazetidine: an unexplored yet versatile organosilane species for ring expansion toward silaazacycles. *J. Am. Chem. Soc.* **2021,** *143*, 11141-11151.
6. Michaelides, I. N.; Darses, B.; Dixon, D. J., Acid-catalyzed synthesis of bicyclo [3. n. 1] alkenediones. *Org. Lett.* **2011,** *13*, 664-667.
7. Smyrnov, O. K.; Melnykov, K. P.; Pashenko, O. Y.; Volochnyuk, D. M.; Ryabukhin, S. V., Stellane at the Forefront: Derivatization and Reactivity Studies of a Promising Saturated Bioisostere of ortho-Substituted Benzenes. *Org. Lett.* **2024,** *26*, 4808-4812.
8. Fritzemeier, R. G.; Nekvinda, J.; Vogels, C. M.; Rosenblum, C. A.; Slebodnick, C.; Westcott, S. A.; Santos, W. L., Organocatalytic trans phosphinoboration of internal alkynes. *Angew. Chem. Int. Ed.* **2020,** *59*, 14358-14362.
9. Fritzemeier, R.; Gates, A.; Guo, X.; Lin, Z.; Santos, W. L., Transition metal-free trans hydroboration of alkynoic acid derivatives: Experimental and theoretical studies. *J. Org. Chem.* **2018,** *83*, 10436-10444.
10. Fujimoto, H.; Kusano, M.; Kodama, T.; Tobisu, M., Three-component coupling of acyl fluorides, silyl enol ethers, and alkynes by P (III)/P (V) catalysis. *J. Am. Chem. Soc.* **2021,** *143*, 18394-18399.
11. Mothes, C.; Lavielle, S.; Karoyan, P., Amino-zinc-ene-enolate cyclization: A short access to cis-3-substituted prolino-homotryptophane derivatives. *J. Org. Chem.* **2008,** *73*, 6706-6710.
12. Patel, M. A.; Kapdi, A. R., Ambient-Temperature, Metal-Free, CDI-Mediated Ex-Situ Conversion of Acids to Amides: A Useful Late-Stage Strategy. *Chem. - Asian J.* **2023,** *18*, e202300672.
13. Inman, M.; Dexter, H. L.; Moody, C. J., Total synthesis of the cyclic dodecapeptides wewakazole and wewakazole B. *Org. Lett.* **2017,** *19*, 3454-3457.
14. Schneider, T. L.; Walsh, C. T.; O'Connor, S. E., Utilization of alternate substrates by the first three modules of the epothilone synthetase assembly line. *J. Am. Chem. Soc.* **2002,** *124*, 11272-11273.
15. Singh, Y.; Sokolenko, N.; Kelso, M. J.; Gahan, L. R.; Abbenante, G.; Fairlie, D. P., Novel cylindrical, conical, and macrocyclic peptides from the cyclooligomerization of functionalized thiazole amino acids. *J. Am. Chem. Soc.* **2001,** *123*, 333-334.

16. Maucort, C.; Vo, D. D.; Aouad, S.; Charrat, C.; Azoulay, S.; Di Giorgio, A.; Duca, M., Design and implementation of synthetic RNA binders for the inhibition of miR-21 biogenesis. *ACS Med. Chem. Lett.* **2021,** *12*, 899-906.
17. Ngo, C.; Fried, W.; Aliyari, S.; Feng, J.; Qin, C.; Zhang, S.; Yang, H.; Shanaa, J.; Feng, P.; Cheng, G., Alkyne as a Latent Warhead to Covalently Target SARS-CoV-2 Main Protease. *J. Med. Chem.* **2023,** *66*, 12237-12248.
18. Zheng, Y.; Li, X.; Ren, C.; Zhang-Negrerie, D.; Du, Y.; Zhao, K., Synthesis of Oxazoles from Enamides via Phenyliodine Diacetate-Mediated Intramolecular Oxidative Cyclization. *J. Org. Chem.* **2012,** *77*, 10353-10361.
19. Bartoccini, F.; Cannas, D. M.; Fini, F.; Piersanti, G., Palladium (II)-catalyzed cross-dehydrogenative coupling (CDC) of N-phthaloyl dehydroalanine esters with simple arenes: stereoselective synthesis of *Z*-dehydrophenylalanine derivatives. *Org. Lett.* **2016,** *18*, 2762-2765.
20. Almaraz-Girón, M. A.; Vázquez, A., Synthesis of 4-benzylidene-oxazol-5 (4H)-imines, structural analogs of PK11195, under Bischler-Napieralski conditions. *Tetrahedron Lett.* **2017,** *58*, 785-788.
21. Shinde, R. S.; Narnawre, A. R.; Karade, N. N., Metal-free intramolecular oxidative cyclization of (Z)-2-benzamido-3-arylacrylates using I_2/DMSO: Synthesis of 2, 4, 5-trisubstituted oxazoles. *Tetrahedron Lett.* **2023,** *117*, 154370.
22. Lukasevics, L.; Cizikovs, A.; Grigorjeva, L., Cobalt-catalyzed C (sp 2)–H bond imination of phenylalanine derivatives. *Chem. Commun.* **2022,** *58*, 9754-9757.
23. Vilaça, H.; Pereira, G.; Castro, T.; Hermenegildo, B.; Shi, J.; Faria, T.; Micaêlo, N.; Brito, R. M. M.; Xu, B.; Castanheira, E., New self-assembled supramolecular hydrogels based on dehydropeptides. *J. Mater. Chem. B.* **2015,** *3*, 6355-6367.
24. Ferreira, P. M.; Monteiro, L. S.; Pereira, G.; Ribeiro, L.; Sacramento, J.; Silva, L., Reactivity of dehydroamino acids and dehydrodipeptides towards N-bromosuccinimide: synthesis of β-bromo-and β, β-dibromodehydroamino acid derivatives and of substituted 4-imidazolidinones. Wiley Online Library **2007**.
25. Mazurkiewicz, R.; Kuźnik, A.; Grymel, M.; Kuźnik, N., ^{1}H NMR spectroscopic criteria for the configuration of N-acyl-α, β-dehydro-α-amino acid esters. *Magn. Res. Chem.* **2005,** *43*, 36-40.
26. CrysAlisPro Software System, Rigaku Oxford Diffraction; Rigaku Corporation: Oxford, UK, 2021.
27.G. M. Sheldrick, *Acta Crystallogr.*, 2015, **71**, 3-8.
28.Sheldrick, G. M. "Crystal structure refinement with SHELXL." Acta Cryst. 2015, C71, 3-8
29. L. J. B. O. V. Dolomanov, R. J. Gildea, J. A. K. Howard and H. Puschmann, *J. Appl. Crystallogr.*, 2009, **42**, 339-341.
30. Persistence of Vision (TM) Raytracer; Persistence of Vision Pty. Ltd.: Williamstown, Victoria, Australia, 2004. http://www.povray.org/.
31. L. J. Farrugia, *J. Appl. Crystallogr.*, 2012, **45**, 849-854.
